# Correction of Distortion in Flattened Representations of the Cortical Surface Allows Prediction of V1-V3 Functional Organization from Anatomy

**DOI:** 10.1371/journal.pcbi.1003538

**Published:** 2014-03-27

**Authors:** Noah C. Benson, Omar H. Butt, David H. Brainard, Geoffrey K. Aguirre

**Affiliations:** 1 Department of Neurology, University of Pennsylvania, Philadelphia, Pennsylvania, United States of America; 2 Department of Psychology, University of Pennsylvania, Philadelphia, Pennsylvania, United States of America; Philipps-University Marburg, Germany

## Abstract

Several domains of neuroscience offer map-like models that link location on the cortical surface to properties of sensory representation. Within cortical visual areas V1, V2, and V3, algebraic transformations can relate position in the visual field to the retinotopic representation on the flattened cortical sheet. A limit to the practical application of this structure-function model is that the cortex, while topologically a two-dimensional surface, is curved. Flattening of the curved surface to a plane unavoidably introduces local geometric distortions that are not accounted for in idealized models. Here, we show that this limitation is overcome by correcting the geometric distortion induced by cortical flattening. We use a mass-spring-damper simulation to create a registration between functional MRI retinotopic mapping data of visual areas V1, V2, and V3 and an algebraic model of retinotopy. This registration is then applied to the flattened cortical surface anatomy to create an anatomical template that is linked to the algebraic retinotopic model. This registered cortical template can be used to accurately predict the location and retinotopic organization of these early visual areas from cortical anatomy alone. Moreover, we show that prediction accuracy remains when extrapolating beyond the range of data used to inform the model, indicating that the registration reflects the retinotopic organization of visual cortex. We provide code for the mass-spring-damper technique, which has general utility for the registration of cortical structure and function beyond the visual cortex.

## Author Summary

A two-dimensional projection of the visual world, termed a retinotopic map, is spread across the striate and extra-striate areas of the human brain. The organization of retinotopic maps has been described with algebraic functions that map position in the visual field to points on the cortical surface. These functions represent the cortical surface as a flat sheet. In fact, the surface of the brain is intrinsically curved. Flattening the cortical surface thus introduces geometric distortions of the cortical sheet that limit the fitting of algebraic functions to actual brain imaging data. We present a technique to fix the problem of geometric distortions. We collected retinotopic mapping data using functional MRI from a group of people. We treated the cortical surface as a mass-spring-damper system and corrected the topology of the cortical surface to register the functional imaging data to an algebraic model of retinotopic organization. From this registration we construct a template that is able to predict the retinotopic organization of cortical visual areas V1, V2, and V3 using only the brain anatomy of a subject. The accuracy of this prediction is comparable to that of functional measurement itself.

## Introduction

The human occipital cortex contains multiple representations of the visual field, starting with primary visual cortex (V1; also called striate cortex). V1 lies primarily within the calcarine sulcus and represents the contralateral visual hemifield. The cortical surface dorsal and ventral to V1 contains the neighboring extrastriate regions V2 and V3, each of which represents a complete visual hemifield that is split into the upper visual quarterfield, ventral to V1, and the lower visual quarterfield, dorsal to V1. These three distinct retinotopic maps are organized on the cortical surface by distance from the fovea (eccentricity) and angle from the vertical meridian (polar angle) [1]. Polar angle sweeps dorsally down and ventrally up from the horizontal meridian in V1 (lying along the calcarine sulcus) around the foveal confluence then reverses direction at the V1/V2 and V2/V3 borders (Fig. 1A). Eccentricity radiates uniformly outward from the foveal confluence in all three visual field maps (Fig. 1B).

**Figure 1:**
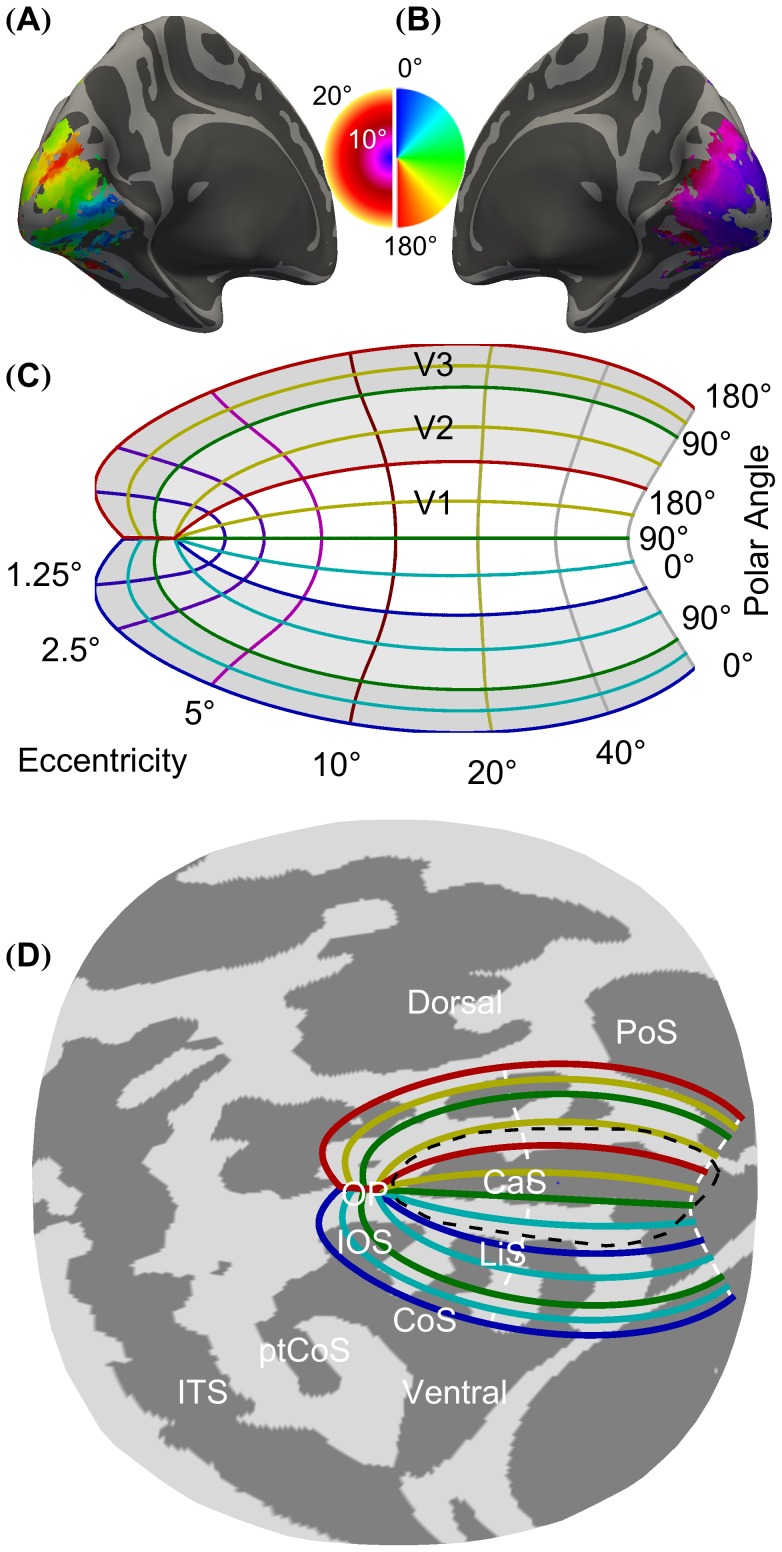
The retinotopic organization of visual cortex as measured and modeled. (**A**) The polar angle map, of a subject from our 10° dataset, shown on an inflated left hemisphere. (**B**) The eccentricity map of the subject shown in part A, shown on an inflated right hemisphere. (**C**) The algebraic model of retinotopic organization. V1, V2, and V3 are colored white, light gray, and dark gray, respectively. (**D**) The cortical surface atlas space (*fsaverage_sym*) from the occipital pole after flattening to the 2D surface. The Hinds V1 border [7] is indicated by the dashed black line, and the algebraic model of retinotopic organization used in registration is plotted with all 0°, 90°, and 180° polar angle lines colored according to the legend and the 10° and 90° eccentricity lines dashed and colored white. Shown are the Calcarine Sulcus (CaS), the Parietal-occipital Sulcus (PoS), the Lingual sulcus (LiS), the Inferior Occipital Sulcus (IOS), the Collateral Sulcus (CoS), the posterior Collateral Sulcus (ptCoS), the Inferior Temporal Sulcus (ITS), and the Occipital Pole (OP).

This visual area organization is readily demonstrated in people by performing retinotopic mapping using functional magnetic resonance imaging (fMRI). When examined in a population of subjects, the qualitative topographical organization of V1–V3 has been found to be consistent [2]. An important advance in the study of retinotopic organization has been the development of software tools for cortical surface registration [3], [4]. The cortical surface is a topological sheet (specifically a sphere), which is thrown into folds (gyri and sulci). The continuous gray matter layer can be identified on an anatomical brain image, represented as a tessellation of vertex points of triangles, and then digitally inflated and flattened to a 2D surface. The pattern of gyral and sulcal curvature is retained and expressed on the flattened cortical sheet. The pattern of cortical surface curvature is then used to drive between-subject registration of brain anatomy on the cortical surface [4]–[6]. When registered in this way, the cortical location of area V1 is found to be consistent across people [7]. More recent work has shown that the size and location of V1, V2, and V3 are also similar across subjects when cortical surface topology is brought into alignment [7], [8].

The functional organization of retinotopic cortex may be captured in an algebraic model. Early algebraic models of V1–V3 used a log-polar transform to relate visual field position to location on the flattened cortical surface [9]. These 2D models were later improved by Schira *et al.* to better capture banding near the foveal confluence and cortical magnification in V2 and V3 [10] (Fig. 1C). Although such models are conceptually useful, the cortical surface as measured in imaging experiments does not respect the details of their idealized geometry. To compare the 2D models to functional measurements, a topological transformation must be applied to the measurements to produce a representation of the data on a flattened surface. Such a transformation, however, necessarily introduces non-trivial geometric distortions that cause the flattened cortical representation to deviate from the idealized, 2D plane in which the algebraic model is defined.

In the limited case of area V1, which resides in a single sulcal fold, we have shown that an algebraic model can be fit to retinotopic mapping data on the flattened cortical surface [11]. In this approach, the fMRI data is brought into alignment across subjects by digital inflation and registration of the cortical surface to a standard anatomic atlas [3]–[5], [12]. Within the 2D, flattened cortical atlas space, we were able to aggregate retinotopic mapping data across subjects and then fit the aggregate data with an algebraic model of retinotopic organization [11]. This linking of algebraic model to the 2D cortical surface atlas then allowed us to accurately predict the functional, retinotopic cortical organization of individual subjects by registering their idiosyncratic brain anatomy to the cortical atlas. The algebraic model provided both a regularization of the data in the presence of noise and generalization of the prediction beyond the boundaries of data itself. Despite the success of this approach within area V1, local geometric distortions of the cortical surface were introduced by the 2D flattening, which in turn distorted the functional prediction of retinotopy (e.g., violation of the equal areal magnification property of retinotopic maps [13], [14]). If we wish to extend this approach to the extrastriate visual areas, we will need to contend with the much greater degree of geometric distortion found in the flattened representation of a larger cortical area that reaches over multiple gyral ridges.

Here we provide a means to link fMRI data from visual areas V1–V3 to an algebraic 2D model of retinotopy in the presence of geometric cortical distortion. One might first consider solving this challenge by modifying the algebraic model to better match the data. Mathematically, however, it is both difficult and poorly descriptive of the fundamental structure of retinotopic organization to tailor a 2D model to the local distortions in geometry present in flattened cortical data. Instead, we propose to register the functional data of the flattened cortical surface to the algebraic model. Such a technique distorts the flattened cortical representation to align the functional data to the algebraic model and is thus flexible enough to correct the geometric distortions introduced by flattening. In this approach, the challenge becomes devising a registration technique that is flexible enough to correct the undesired distortions and adequately align to the algebraic model yet sufficiently constrained so that the resulting, registered anatomy retains its structure enough to support generalization of the algebraic model beyond the extent of the data used in the registration.

Mass-spring-damper (MSD) systems are commonly used in the simulation of the deformation of materials and objects [15]–[17]. These systems approximate surfaces or volumes as a series of point masses connected in a mesh by ideal springs (i.e., a spring whose applied force is proportional only to the displacement of the spring). Because of the simplicity of the forces enacted by an ideal spring, simulation of such systems by means of numerical integration is relatively straightforward.

Similar to our prior work in V1, we obtain across-subject retinotopic mapping data that is then aggregated within a standard cortical surface atlas [4]–[6]. We then represent the cortical atlas surface and aggregate retinotopic mapping data as an MSD system which places two sets of springs in opposition. First, all cortical vertices are treated as point masses connected by ideal springs to their neighbors. This spring set resists warping the anatomy of the cortex. A second set of springs connects each cortical point that has a retinotopic mapping value to a fixed position in an overlying algebraic model of retinotopy. This spring set works to modify the cortex to bring the functional data into best alignment with the algebraic model. The simulation identifies a low-energy state of the system which balances these competing forces.

The result of the MSD simulation maps individual vertices within the cortical surface atlas to a specific visual area and visual field position. We show that this mapping may be used to accurately predict the retinotopic organization of extrastriate cortex in a novel subject who's brain anatomy is brought into register with the cortical surface atlas. Further, because the algebraic model is continuous, we find that the mapping may be used to accurately predict retinotopic data collected from beyond the eccentricity range of data used in the aggregate to derive the mapping.

## Methods

### Ethics statement

This study was approved by the University of Pennsylvania Institutional Review Board, and all subjects provided written consent.

### Subjects and stimuli

A total of 25 subjects (15 female, mean age 24, range 20–42) participated in fMRI scanning experiments. All subjects had normal or corrected-to-normal vision. Experimental data from all subjects have been reported previously [11]. Each subject contributed to only one of two datasets.

The first dataset, D_10°_, contained 19 subjects all of whom were scanned for 27 minutes using a sweeping bar stimulus that extended to 10° of eccentricity within a central 20° aperture. The bar stimulus consisted of a single sweeping 2.5°-thick bar that flickered at 5 Hz [18]. Bars moved 1.25° every 3 s in 4 directions (horizontal, vertical, oblique +45°, oblique −45°) while subjects maintained central fixation.

The second dataset, D_20°_, contained 6 subjects. Subjects fixated on either the left or right edge of the screen for 64 minutes while 16 iterations of standard “ring and wedge” stimuli swept in the periphery [19].

### Magnetic resonance imaging

BOLD fMRI data (TR = 3 s, 3 mm isotropic voxels) and anatomical images (T1-weighted, 1 mm isotropic voxels) were collected at 3 Tesla. The FMRIB Software Library (FSL) toolkit (http://www.fmrib.ox.ac.uk/fsl/) was used to process anatomical images which were then reconstructed and inflated using FreeSurfer (v5.1) (https://surfer.nmr.mgh.harvard.edu/) [3]–[6]. Hemispheres from individual subjects were aligned via surface registration to FreeSurfer's common left-right symmetric pseudo-hemisphere (*fsaverage_sym*) [3], [12].

For subjects in the D_10°_ dataset, a population average hemodynamic response (HRF) [20] was used to model the BOLD signal. For subjects in dataset D_20°_, a subject-specific HRF was derived from a separate blocked visual stimulation scan. Global signal, cardiac and respiratory fluctuations (when available) [21], effects of the scan, and spikes (i.e., instances in which the signal deviates from the mean by ≥2 standard deviations) were modeled as nuisance covariates. Polar angle and eccentricity were either modeled (with receptive field size) using the population receptive field (pRF) method [18] (datasets D_10°_) or by identification of the peak of a Gaussian fit to the weights of a set of finite impulse response covariates (dataset D_20°_) [22].

### Preparation of retinotopic data

Aggregate retinotopic maps of each dataset were produced separately for polar angle and eccentricity by finding the weighted mean polar angle and eccentricity of all subjects at each aligned vertex position. Mean polar angles and eccentricities were weighted by the *F*-statistic of the confidence of each subject's polar angle and eccentricity assignments. A confidence for each vertex in the aggregate was calculated as the sum of squares of the *F*-statistics of all significant vertices divided by the sum of the same *F*-statistics. For a set of subjects **Q**, each of whom have a vertex at position *p* on the cortical surface with a polar angle and eccentricity assignment whose significance is above threshold, the confidence of aggregate vertex *p* is (Σ*_q_*_•**Q**_ *F*(*q*, *p*)^2^)/(Σ*_q_*_•**Q**_ *F*(*q*, *p*)) where *F*(*s*, *x*) is the confidence of the polar angle and eccentricity assignment in subject *s* at vertex position *x*. The assignment of any vertex whose confidence was below a minimum threshold chosen for the dataset (see Supplemental *Mathematica* Notebook, §3.2), was discarded. Because averaging produces bias in the direction of the mean near the borders of a finite stimulus range (*e.g.*, values near 0° and 180° of polar angle tend to attenuate toward 90° in the aggregate), the aggregate polar angle values were corrected and eccentricity was truncated by 1.25°. Polar angle correction was performed by forcing the distribution of polar angles in the corrected aggregate to match the distribution of the union of all significant polar angle values of all subjects. More specifically, the uncorrected aggregate polar angle *θ* of each vertex in the aggregate was changed to a corrected polar angle *θ′* such that *C*(*A*, *θ*) = *C*(*M*, *θ′*) where *C*(*D*, *t*) is the cumulative density function of the distribution *D*, evaluated at *t*, and *A* and *M* are the distributions of the uncorrected aggregate polar angles and union of all significant polar angle values for all subjects, respectively. Eccentricity values below 1.25° and within 1.25° of the outer stimulus border were excluded due to measurement bias near the edge of the stimulus range [23].

All vertices within *π*/3 radians on the inflated spherical hemisphere of the point *p*_0_, defined as the most anterior point on the anatomically defined V1 border [7], were rotated such that *p*_0_ lay at the intersection of the equator of the spherical *fsaverage_sym* brain hemisphere and prime meridian, then flattened via projection onto the plane tangent to the sphere at *p*_0_. A shear transformation, present also in our previous treatment of V1 [11], was applied to the flattened data to render the V1 region more elliptical. These flattened and sheared data formed a “flattened occipital region” on the cortical surface.

### Registration to an algebraic model of retinotopy

Data from D_10°_ were registered to a modified version of the banded double-sech model proposed by Schira *et al.* [10] using a simulated mass-spring-damping system. Each vertex in the flattened occipital region was assigned an initial position identical to its position in the flattened occipital region and a mass of 1 g. Vertex coordinates were measured in radians (rad) according to their angular latitude (*y*-coordinate) and longitude (*x*-coordinate) relative to *p*_0_ (described above) on the *fsaverage_sym* spherical hemisphere. All pairs of vertices whose initial positions were within 0.015 rad of each other were connected by a spring whose ideal length was equal to the initial distance between the vertices and whose stiffness was 1.0 g/s^2^. These “anatomical springs” ensured that warping introduced during the simulation would respect anatomical constraints. Additionally, for each vertex with an above confidence threshold assignment of eccentricity and polar angle in the dataset aggregate, a “model spring” with one fixed and one free end was connected between the vertex (free end) and the position predicted by the algebraic model for the aggregate observed polar angle and eccentricity of the vertex (fixed end). Because there are multiple such points (*i.e.*, in V1, V2, and V3) for each polar angle and eccentricity, the fixed end of the spring was constantly updated throughout the simulation to be positioned at the nearest such point. Model springs were assigned an ideal length of 0 rad and a stiffness of 10 g/s^2^. To prevent vertices distant from the algebraic model but with polar angle and eccentricity assignments nonetheless above our *F*-value threshold from having an overly large influence on the simulation due to their high spring length, the potential function of the vertex attached to a model spring was represented as an inverted Gaussian whose center was the ideal position for the vertex in the algebraic model of retinotopy instead of a parabola with the same center. The choice of a Gaussian potential function for use in aligning retinotopic data on the cortical surface is similar to the energy function proposed by Fischl *et al.* [3] for aligning hemispheres by curvature. Note that because the force acting on the vertex is the gradient of the potential of that vertex, this choice of potential function effectively means that the force acting on a vertex either very close to or very far from its ideal position is near zero. For a spring of length *d* and stiffness *k*, the magnitude of the force acting on the ends of an anatomical spring with ideal length *d*_0_ is *k* |*d* - *d*_0_|; for the endpoint of a model spring, the magnitude of the force is 4*k* |*d* - *d*_0_| exp(−64(*d* - *d*_0_)^2^), which approximately models the force of a parabolic spring at small distances. An additional force was applied to all pairs of vertices not bonded by springs such that any such pair of vertices within a given distance *d*, less than some cutoff *c*, of each other were repelled by (4 *c*/(*d*+*c*)−2) rad g/s^2^; in our simulations, *c* was chosen to be half the average anatomical spring length. This “van der Waals”-like force prevents vertices from passing through each other. The motion of all vertices was dampened by 0.1% after each step (*i.e.*, each vertex's velocity was multiplied by 0.999 after each simulation step). Further details concerning the parameterization of the simulation and the stability of these parameters can be found in the Supplemental Materials.

The algebraic model of retinotopic organization was modified from that of Schira *et al.* [10] by the addition of parameters for translation, rotation, and horizontal and vertical stretch, all of which were necessary to produce an initial fit to the aggregate functional data. The original double-sech model includes parameters *a*, *b*, *k*, and *λ*. We retain *a*, *b*, and *λ*, but replace *k*, the scale parameter, with horizontal and vertical scales. Although this breaks certain features of the original Schira model such as the consistency of areal magnification, we note that this point is essentially moot as we are dealing with distorted data already and are further warping it during registration to the model. Accordingly, we focus on the parameters *a*, *b*, and *λ*, which define the shape of the model and for which we use values 1.5, 60, and 2.5 respectively. This parameterization was found by manipulating parameters “by hand” to align them with the aggregate retinotopy; code for experimenting with this fit is provide in our Supplemental *Mathematica* Notebook (§1.6.5). An additional “V4-like” dorsal and ventral region was added to the model to stabilize vertices in both V3A and hV4 whose retinotopy would otherwise cause them to be attached via model springs to V3. The full parameterization of the algebraic model of retinotopy and source code for calculating and inverting it are provided in the Supplemental *Mathematica* Notebook, §1.6.

Simulation was performed by numerical integration of the system using a time-step size of 5 ms. At each step *t*, acceleration values were calculated for each vertex using Newton's second law of motion. Positions were updated such that **x** *_t+1_* = **x** *_t_*+**v** *_t_ ∂t*+**a** *_t_ ∂t* ^2^/2 and velocities were updated such that **v** *_t_*_+1_ = **v** *_t_*+**a** *_t_ ∂t*, where **x** *_t_*, **v** *_t_*, and **a** *_t_* are the position, velocity, and acceleration vectors of a given vertex at step *t*, and *∂t* is the step size. Vertices were given small random initial velocities such that the net velocity at time 0 was 0 but such that the total KE of the system was 10 rad^2^⋅g/s^2^. Energies were examined every 10 steps and KEs were rescaled whenever the total energy (PE+KE) exceeded the initial energy (PE_0_) by at least 2 rad^2^ g/s^2^ due to numerical drift. Simulations were run with a step size of 2 ms for 5,000 steps (10 s). After simulation, the resulting configuration was minimized by a simple gradient descent search using a gradient step distance of 0.005 for 500 steps or until convergence. Source code for the simulation is provided via a gitHub repository (http://github.com/NoahBenson/SpringRegister/).

By simulating the system until a low PE is achieved, we allow the constraints imposed by both the cortical anatomy and the functional model to relax into a solution that respects both kinds of information. Because the simulation incorporates KE, a nonlocal energy minimum may be found; it is therefore beneficial to use simulated annealing. Four simulations of 10 s (5,000 steps) each were performed such that the final arrangement of vertices in each simulation was used as the starting arrangement for the next simulation; spring ideal lengths were not recalculated, however, and the velocities were re-randomized such that the KE of the system was 10 rad^2^ g/s^2^ at the beginning of each simulation. The final arrangement of the four simulations with the lowest PE was chosen as the arrangement of the corrected topology. Vertices were assigned model polar angle and eccentricity values from their positions in the corrected topology by inverting the algebraic model of retinotopy. In other words, if the algebraic model of retinotopy predicts that a point (*θ*, *ρ*) in the visual field should lie at position (*x*, *y*) on the cortical surface, then a vertex with position (*x*, *y*) in the corrected topology would be assigned a polar angle of *θ* and an eccentricity of *ρ*.

## Results

Retinotopic mapping data was obtained from 19 subjects to an eccentricity of 10° of visual angle (dataset D_10°_) (Fig. 1A, 1B). The brain anatomy from each subject was registered to an atlas of cortical surface topology (*fsaverage_sym*), and the across-subject, confidence-weighted mean aggregate of polar angle and eccentricity obtained. The reversals of polar angle that mark the boundaries of visual areas, and the regular progression of eccentricity from the occipital pole, is readily seen in the aggregate data. The goal of our work is to register the measured retinotopy in the volume with an algebraic model on the flattened cortical surface (Fig. 1C). The registration is performed within a flattened patch of the cortical surface atlas (Fig. 1D).

Registration via MSD simulation brought the aggregate retinotopic mapping values into alignment with the algebraic model by warping the cortex. The magnitude and direction of warping induced by this registration (*i.e.*, the distance and angle between each vertex position in the flattened *fsaverage_sym* atlas space and its position in the corrected topology) is shown in Figs. 2A and 2B. Notably, the greatest displacement of vertices is found around the occipital pole. We presume that the warping of the cortex in our registration is correcting the geometric distortions created during flattening of this region of high curvature. The sulcal folding pattern of the original cortical surface atlas and the corrected topology following MSD simulation are shown in Figs. 2C and 2D respectively, along with the regional assignment (V1, V2, or V3) predicted by applying the algebraic model of retinotopic organization to vertices in the corrected topology.

**Figure 2:**
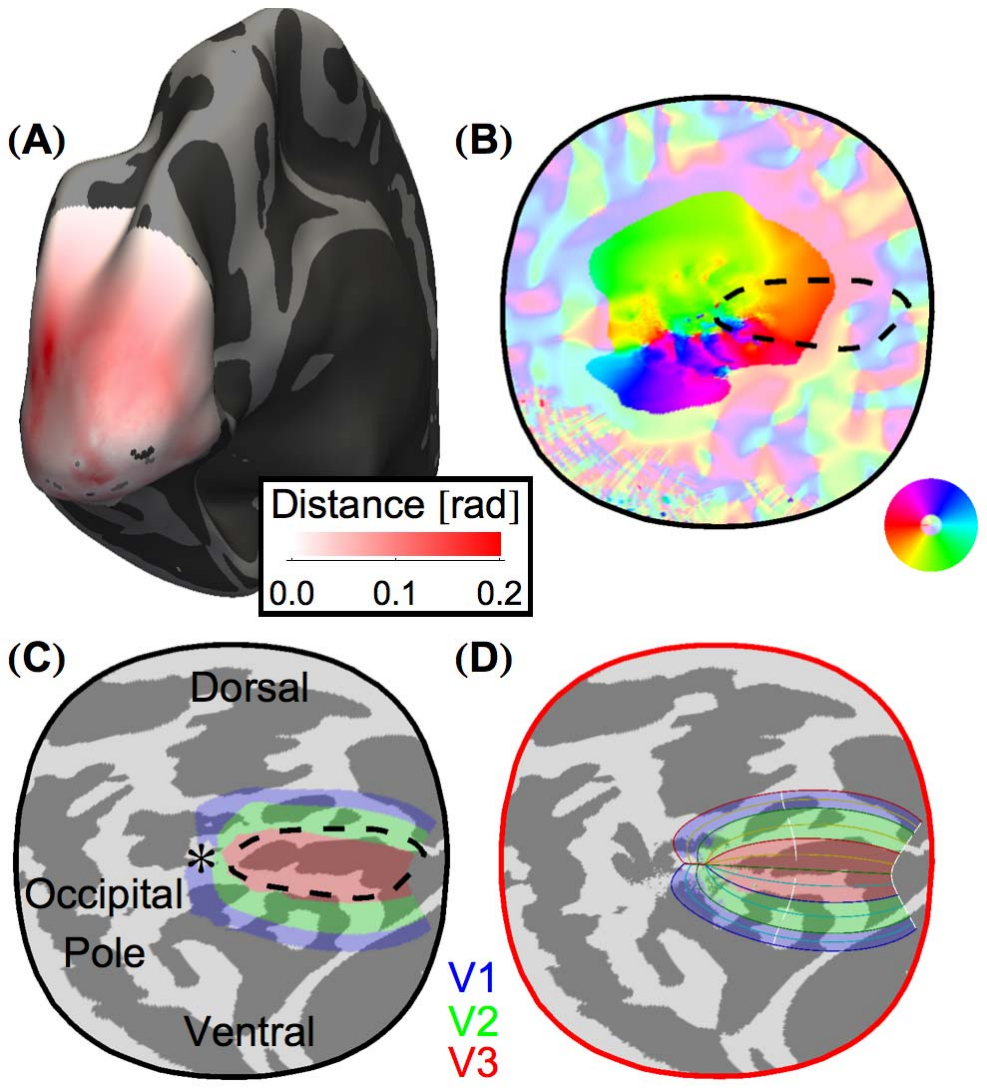
MSD simulation warps the flattened cortical surface to register data to the algebraic model. (**A**) The magnitude of the warping of each vertex on the flattened cortical surface. The Hinds *et al.* V1 border [7] is marked by the dashed black line (throughout). (**B**) The direction of warping induced by MSD simulation upon each vertex from the original cortical surface atlas space (*fsaverage_sym*). Vertices with distortion magnitudes below 0.01 rad are plotted in unsaturated colors. (**C**) The flattened patch of the cortical surface atlas (*fsaverage_sym*) with sulcal curvature shown in light and dark gray. Regions V1, V2, and V3, as predicted by our method in the corrected topology then projected back to surface atlas space, are tinted red, green, and blue respectively. (**D**) The flattened cortical surface of the corrected topology with sulcal curvature shown in light and dark gray. A line plot of the algebraic model (Fig. 1C) to which the MSD simulation registered the functional data is shown. Regions V1, V2, and V3 are tinted as in panel C.

### Prediction of polar angle

When aggregated within the cortical surface atlas, polar angle organization is largely consistent across subjects. A flattened aggregate map of the confidence-weighted mean polar angle of the 19 subjects in our 10° eccentricity dataset D_10°_ is shown in Fig. 3A. Although regional boundaries in the aggregate map are apparent, the iso-angular curves in this organization do not resemble the smooth curves found in the algebraic model of retinotopic organization (Fig. 1C), suggesting an opportunity for registration via simulation to improve the predictive accuracy of the algebraic model. The polar angle organization following the MSD simulation is shown in Fig. 3B. As would be expected, minimization of energy in the MSD simulation has warped the cortex to bring the aggregate polar angle data into better alignment with the algebraic model of retinotopic organization. The algebraic model of retinotopy can then be projected back to the original cortical surface atlas (Fig. 3C). This smooth, continuous map of polar angle organization should resemble the measured polar angle functional data of any subject following the registration of their brain anatomy to the cortical surface atlas. We therefore refer to this representation as an anatomical template of retinotopy.

**Figure 3:**
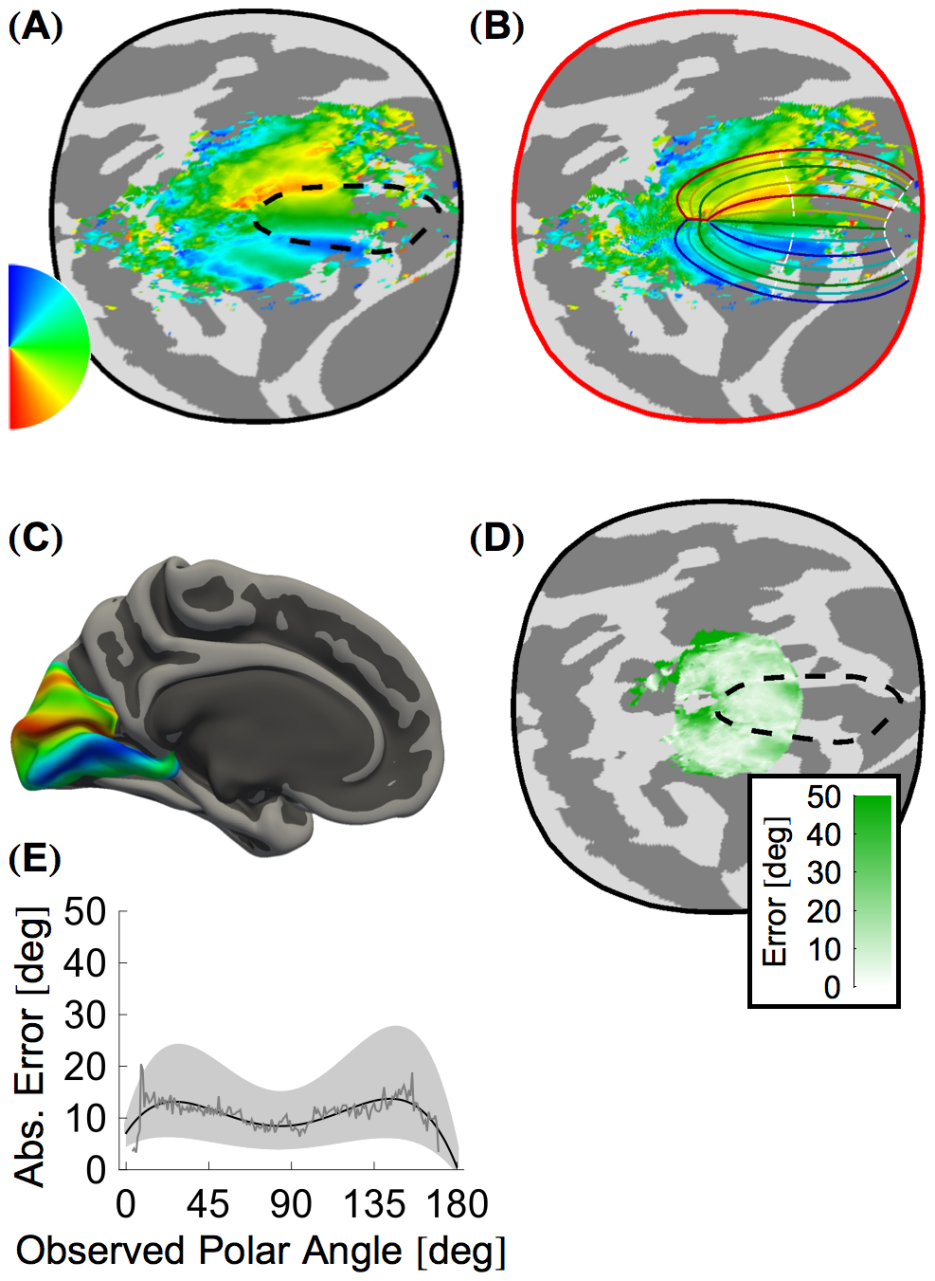
Polar angle organization. (**A**) The mean weighted aggregate polar angle map of all subjects in dataset D_10°_ shown in the cortical surface atlas space. (**B**) The mean weighted aggregate polar angle map from panel A shown in the corrected topology following MSD warping. A line plot of the algebraic model to which the MSD simulation registered the functional data is shown over the functional data. (**C**) The polar angle template plotted on the *fsaverage_sym* pial surface. This template was calculated by converting the prediction of polar angle from the idealized model, as applied to vertices in the corrected topology, back to the *fsaverage_sym* atlas. (**D**) Median absolute leave-one-out polar angle error for all vertices with predicted eccentricties between 1.25° and 8.75° shown in the *fsaverage_sym* atlas space. This error was calculated by comparing the predicted polar angle generated from each subset of 18 of the 19 subjects in the 10° dataset to the observed polar angle of the remaining subject. The median absolute overall leave-one-out error is 10.93° (Tab. 1). The highest errors occur near the foveal confluence and at the dorsal border of V3. (**E**) Absolute leave-one-out error of the polar angle prediction across all regions (V1, V2, and V3), plotted according to the predicted polar angle value. The thin gray line represents the median error while the thick black line shows a best-fit 5th order polynomial to the median error. The dashed lines demarcate similar fits to the upper and lower error quartiles. Error plots for individual regions are given in Fig. S1.

To examine how well our template predicts a subject not previously seen, we calculated leave-one-out errors. To do so, the aggregate polar angle data was obtained from 18 subjects. The cortical surface atlas was then warped by MSD simulation to match the aggregate to the algebraic model of retinotopy. Finally, the algebraic model was projected back to the surface atlas and used to predict the polar angle organization of the left out subject.

Leave-one-out errors in the polar angle prediction were non-uniform across striate and extrastriate cortex (Figs. 3D and 3E). The highest errors are visible near the foveal confluence where all iso-angular lines converge, as well as in the dorsal region of V3 where V3 borders V3A. Although the median absolute leave-one-out error was uniformly low for a given predicted polar angle when aggregated across all three regions (Fig. 3E), errors in V3 were higher than those in V1 or V2, particularly close to the outer borders (Fig. 3D; Fig. S1). Overall, the median absolute and signed leave-one-out errors across all subjects and all vertices between observed and predicted polar angle were 10.93° and −0.48° respectively (Tab. 1). Additional reports and plots of the error in these predictions can be found in our Supplemental *Mathematica* Notebook (§5 and §6.3-6).

**Table 1:** Errors by visual area for dataset D_10°_.

Polar Angle Error^a^					
Area	Absolute^b^	Signed^c^	Aggregate^d^	Unregistered^e^	Split-half^f^
V1	10.48°	2.01°	21.24°	33.20°	13.78°
V2	11.12°	−3.17°	16.15°	37.52°	7.13°
V3	11.73°	3.35°	20.51°	37.34°	9.86°
All	10.94°	0.58°	16.48°	37.28°	7.50°

Eccentricity Error^a^					
Area	Absolute	Signed	Aggregate	Unregistered	Split-half
V1	0.41°	0.26°	0.61°	6.20°	0.68°
V2	0.34°	−0.06°	0.96°	10.38°	0.50°
V3	0.33°	0.10°	1.08°	3.07°	0.63°
All	0.37°	0.15°	0.96°	5.94°	0.50°

a Errors are calculated in a typical leave-one-out fashion in which each subject is compared to the prediction found using all other subjects; all significant vertices between 1.25° and 8.75° of eccentricity are included, and the reported errors represent the median of all vertices from all subjects.

b Median absolute leave-one-out error between expected and observed values of all vertices.

c Median signed leave-one-out error, expected value minus observed value, of all vertices.

d Median absolute leave-one-out error, as calculated by predicting the polar angle and eccentricity of the left-out subject from the confidence-weighted mean of all other subjects.

e Median absolute error between observed values and those predicted by the algebraic model of retinotopy prior to any registration.

f Median absolute error between observed values from two identical 20 minute scans.

Vertices for which the *F*-statistic of the polar angle and eccentricity assignments were below 5 were discarded.

The quality of the polar angle predictions provided by the MSD approach may be compared to the prediction accuracy obtained using only the aggregate retinotopy data (similar to the approach of [8]). We calculated a mean-weighted average polar angle map for each subset of 18 of the 19 subjects in D_10°_ and used each of these maps to predict the polar angle and eccentricity of the excluded subject. The median absolute leave-one-out polar angle error between all significant vertices of all subjects and the appropriate leave-one-out aggregate vertices was 23.27° (Tab. 1), twice as large as was obtained using the MSD approach. This indicates that the MSD approach serves as an informed regularization of noise that is present even in the average retinotopic mapping data from 18 subjects.

Finally, we examined how well the algebraic model of retinotopic organization, prior to spring registration to the aggregate data, predicts retinotopy in individuals. Again, the median absolute polar angle error of 34.12° was much greater than that obtained following MSD warping of the aggregate data to the algebraic model. This indicates that our approach corrects consequential distortions introduced by cortical flattening.

### Prediction of eccentricity

Fig. 4A presents the aggregate, confidence-weighted mean eccentricity of the 19 subjects in D_10°_. As with polar angle, the organization of eccentricity is consistent across subjects combined in the cortical surface atlas space, but sharp bends in the iso-eccentric contours, *e.g.* at the borders of V1 near 8–10° of eccentricity, do not match the properties of the algebraic model. The aggregate eccentricity, when warped to the algebraic model using MSD simulation, now follows the smooth lines of the idealized model (Fig. 4B). As with polar angle, the algebraic model may be projected back to the cortical surface atlas (Fig. 4C), to create an anatomical template of retinotopy which may be used to predict the retinotopic organization of novel subjects.

**Figure 4:**
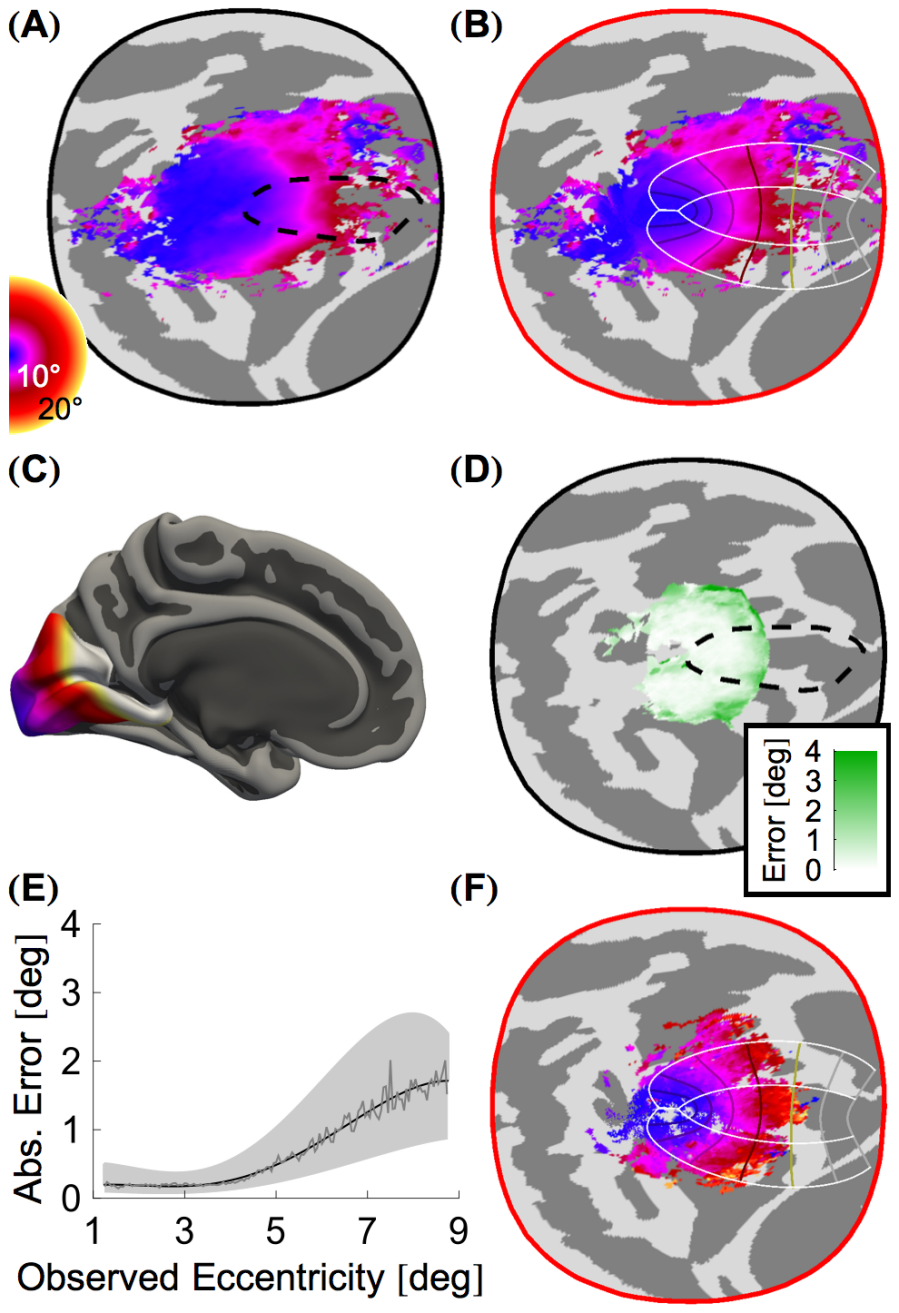
Eccentricity organization. (**A**) The mean weighted aggregate eccentricity map of all subjects in dataset D_10°_ shown in the *fsaverage_sym* cortical atlas space. (**B**) The mean weighted aggregate eccentricity map from panel A shown in the corrected topology following MSD warping. A line plot of the algebraic model to which the MSD simulation registered the functional data is shown. (**C**) The eccentricity template plotted on the *fsaverage_sym* pial surface. This template was calculated by converting the prediction of eccentricity from the algebraic model, as applied to vertices in the corrected topology, back to the *fsaverage_sym* topology. (**D**) Median absolute leave-one-out eccentricity error for all vertices with predicted eccentricties between 1.25° and 8.75° shown in the *fsaverage_sym* atlas space. This error was calculated by comparing the predicted eccentricity generated from each subset of 18 of the 19 subjects in the 10° dataset to the observed eccentricity of the remaining subject. The median absolute overall leave-one-out error is 0.41° (Tab. 1). The highest errors occur near the outer eccentricity border of of our stimulus. (**E**) Absolute leave-one-out error of the eccentricity prediction across all regions (V1, V2, and V3), plotted according to the predicted polar angle value. Error plots for individual regions are given in Fig. S2. (**F**) The mean weighted aggregate eccentricity map of all subjects in dataset D_20°_ shown in the cortical patch corrected by MSD warping to the D_10°_ dataset. Although this dataset includes eccentricities beyond those used to discover the corrected topology, the 20° aggregate data is in good (although not perfect) agreement with the prediction.

Median absolute leave-one-out errors for eccentricity were low across V1–V3 (Fig. 4D), with only slightly higher errors at greater eccentricities (Fig. 4E). Eccentricity error, unlike polar angle error, was uniform in V1, V2, and V3 (Fig. S2). The absolute and signed median leave-one-out errors for all subjects and vertices in D_10°_ were 0.41° and 0.05° respectively (Tab. 1).

As was found for polar angle, simply using the aggregate polar angle data without MSD registration to the algebraic model resulted in substantially worse prediction accuracy for left-out subjects (median absolute error of 1.53°; Tab. 1). This was true as well for the attempt to predict eccentricity using the algebraic model but without MSD driven warping of the cortex (median absolute error of 2.44°).

### Extrapolation of model predictions

The accuracy of polar angle and eccentricity prediction suggests that the algebraic model following MSD warping fits the retinotopic arrangement in regions V1–V3 well. This accuracy of prediction, however, does not necessarily indicate that the algebraic model is a good general representation of retinotopic organization. This is because MSD warping could in principle force the retinotopic data to match any locally smooth model which would then serve to regularize the data in the face of noise and thus improve prediction. While the “anatomical” springs used in the MSD simulation make an extreme warping to a very poor algebraic model implausible, an explicit test of the generalizability of the approach is desirable. If the algebraic model of retinotopy accurately describes the functional arrangement of the visual cortex, our approach should extrapolate to the prediction of eccentricity and polar angle in regions of visual cortex beyond the retinotopic mapping data.

To test the generality of the algebraic model and our template, we compared the anatomical template of retinotopy derived from D_10°_ to the aggregate retinotopic mapping data from D_20°_, which consists of a separate set of subjects whose retinotopic maps were found using different techniques that doubled the mapped eccentricity range to 20° (see Methods).

For polar angle, the median absolute and signed errors between the measured and predicted value were 14.58° and 0.99° respectively. Note that these errors are comparable to those from the D_10°_ leave-one-out analyses despite the fact that the D_20°_ data extends beyond the 10° of eccentricity used to fit the model.

We next examined eccentricity prediction. Fig. 4F presents the median aggregate eccentricity map from D_20°_ in the corrected cortical surface space found using D_10°_ (the D_20°_ aggregate in the original cortical atlas space is presented in the Supplemental *Mathematica* Notebook, §6.4.1). The overall median absolute error between vertices in D_20°_ and the eccentricity template was 0.77°. Notably, this error is lower in the region from 1.25° to 8.75° (median absolute error: 0.59°) and higher in the region from 8.75°–18.75° (median absolute error: 2.33°). This suggests that our ability to fit extended data with our template is good but imperfect.

### Measurement error

Our prediction error incorporates both the imperfections of our template as well as error in the measurement of retinotopy in the individual subject to be predicted. We have previously reported that the error in measured polar angle and eccentricity between two identical 20 minute retinotopic scans is ∼0.75° of eccentricity and ∼7.76° of polar angle in area V1 [11]. Similar statistics, using the new definition of region V1 we have derived here as well as the definition of V2 and V3, are given in Table 1 and plotted in Fig. S3. Measurement error grows from V1 to V3 as does prediction error. Notably, measurement error is actually greater than the prediction error of our anatomical template of retinotopy in all visual areas except for polar angle in V3.

## Discussion

We have described a technique to register functional data on the cortical surface to a 2D algebraic model of cortical organization. This approach allows us to predict the location and organization of visual areas V1–V3 in individual subjects based only upon an anatomical image of their brain. The overall prediction error for V1–V3 (10.93° of polar angle, 0.51° of eccentricity) is actually somewhat lower than the error we observed in our previous V1 template alone (11.43° of polar angle, 0.91° of eccentricity) [11]. We attribute this improvement to the correction of geometric distortions in the cortex introduced by flattening. Overall, we found that the accuracy of anatomically-based prediction of retinotopy in the individual subject is comparable to that provided by functional measurement itself.

In addition to good prediction accuracy, the anatomical template of V1–V3 retinotopy had generally small and uniform prediction bias. An exception to this property was found at the dorsal V3/V3A border, where our template consistently over-predicts the observed polar angle values (Fig. 3E). The error near the dorsal V3 border is substantially higher than that of any other region we studied (Fig. S1). The much smaller error found near the ventral V3/hV4 boundary suggests that the error is not due to a general inability of the approach to fit the outer boundaries of a model. Instead, we find that the dorsal V3 error can be understood as the effect of the V3A region extending into the V3 region during registration. Examination of the aggregate polar angle map (Fig. 3A, B) indicates that the predicted dorsal V3 boundary passes through a region of cortex that should be assigned by the template to V3A. This misalignment results from an attenuated polar angle reversal near the dorsal V3 border as compared to other reversals, which can be observed in Fig. 3A.

One possible explanation of this attenuated reversal is that it is the result of a poor across-subject anatomical registration due to variability in sulcal topology between subjects that could not be aligned. Such a problem would result in poorly aligned vertices and variable values contributing to the aggregate at this location. Examination of the sulcal curvature of individual subjects, however, does not support the idea of greater sulcal variability in this region (Fig. S4A).

An alternate explanation of the error near the dorsal V3 boundary is that individual differences in the mapping between structure and function create an area of relatively poor fit. Indeed, if we assume that the anatomical registrations provided by FreeSurfer are unbiased, there do appear to be significant differences in the location of the V3/V3A boundary between subjects (Fig. S4B). However, Fig. S3 shows a concentration of error in this same dorsal region, as well as near the foveal confluence, for the split-halves (test-retest) measurement error. Retinotopy in this region may simply be more difficult to measure.

It is entirely possible that the dorsal V3 border, and more generally the quality of the entire template, could be improved with modifications of our approach. We presented here a particular algebraic model of retinotopy [10] linked to the cortical surface with a particular deformation technique (MSD simulation). Neither of these choices are integral to the approach we describe. We selected the MSD approach as it provided an explicit means to balance maximizing registration of retinotopic values to the algebraic model against minimizing anatomical warping. Other approaches are certainly possible. In the Supplemental *Mathematica* Notebook we provide an example of an alternative registration method (see §1.8, Delaunay Mesh Registration, for implementation; §5.1.2 and §5.2.2 for error reports; and §6.2.3, §6.5.4-6, and §6.6.4-6 for figures).

More broadly, we consider the key insight of our work to be that geometric distortion of the flattened cortical surface limits the application of idealized models of cortical organization to empirical measurements of cortical function. These distortions, whether introduced by the developmental process of cortical folding or the digital process of cortical flattening, may be corrected by warping the cortical surface to bring function and model into alignment. Here, we demonstrated the practical value of this approach by creating an anatomical template of retinotopic organization. We expect that other early sensory areas such as the sensorimotor and auditory cortex, as well as higher level visual areas such as motion and face sensitive cortex, could be modeled using similar methods. This paper and its supplemental materials are intended as a guide for these kinds of studies.

## Supporting Information

Figure S1 Polar angle prediction error for (A) V1, (B) V2, and (C) V3. In each figure, the gray line shows the median absolute leave-one-out error for vertices based on their predicted polar angle. The black line shows a best-fit fifth order polynomial to the median. The shaded regions show the extent of the upper and lower quartile of the errors. A spike in median absolute error can be seen near 90° of polar angle in both V2 and V3; this spike is due to error in a region near the foveal confluence (Fig. S3).
(TIF)

Figure S2 Eccentricity prediction error for (A) V1, (B) V2, and (C) V3. In each figure, the gray line shows the median absolute leave-one-out error for vertices based on their predicted polar angle. The black line shows a best-fit fifth order polynomial to the median. The shaded regions show the extent of the upper and lower quartile of the errors.
(TIF)

Figure S3 Split-halves test/retest errors for (A) eccentricity and (B) polar angle. Test/retest error at a particular vertex position were calculated as the median absolute difference in measured (*i.e.*, deduced from the BOLD signal only) eccentricity or polar angle between the first and second 20-minute halves of the scans of all subjects in D_10°_. The dashed black line shows the Hinds *et al.* V1 border. Data for vertices with eccentricities between 1.25° and 8.75° are shown. Notably, a patch of error near the foveal confluence and near the dorsal V3/V3A border exists in both eccentricity and polar angle, suggesting that these regions are difficult to measure accurately.
(EPS)

Figure S4 Exploration of the dorsal V3/V3A border. (A) The standard deviation (left) and median (right) sulcal curvature across all subjects and hemispheres. The dashed red outline indicates the region from which plots in B are taken. (B) Contour plots of polar angle for all subjects' dorsal V2/V3/V3A regions. Contour lines are drawn at 0°, 30°, 60°, 90°, 120°, 150°, and 180°. In the lower right corner, the 150° contour lines for all subjects are shown together. Although the V1/V2 border reversal is relatively conserved, much less agreement can be found for the V3/V3A reversal.
(TIF)

File S1 Supplemental *Mathematica* Notebook, rendered as a PDF file, containing source code and tools for the analysis of registered cortical surfaces. A version of this file in *Mathematica*'s native notebook format (.nb file) is available for download from our lab website: https://cfn.upenn.edu/aguirre/wiki/public:data_ploscomputbiol_2014_benson.
(PDF)

Text S1 Supplemental notes regarding the parameterization of the simulation and Schira model. This document provides a brief description of our simulation and model parameterization as well as information about where to find additional materials such as source code.
(DOC)
