# Supplementary figures and images for: Correction of Distortion in Flattened Representations of the Cortical Surface Allows Prediction of V1-V3 Functional Organization from Anatomy
Source: PLoS Comput Biol. 2014 Mar 27;10(3):e1003538. doi: 10.1371/journal.pcbi.1003538 (PMC3967932; doi:10.1371/journal.pcbi.1003538)

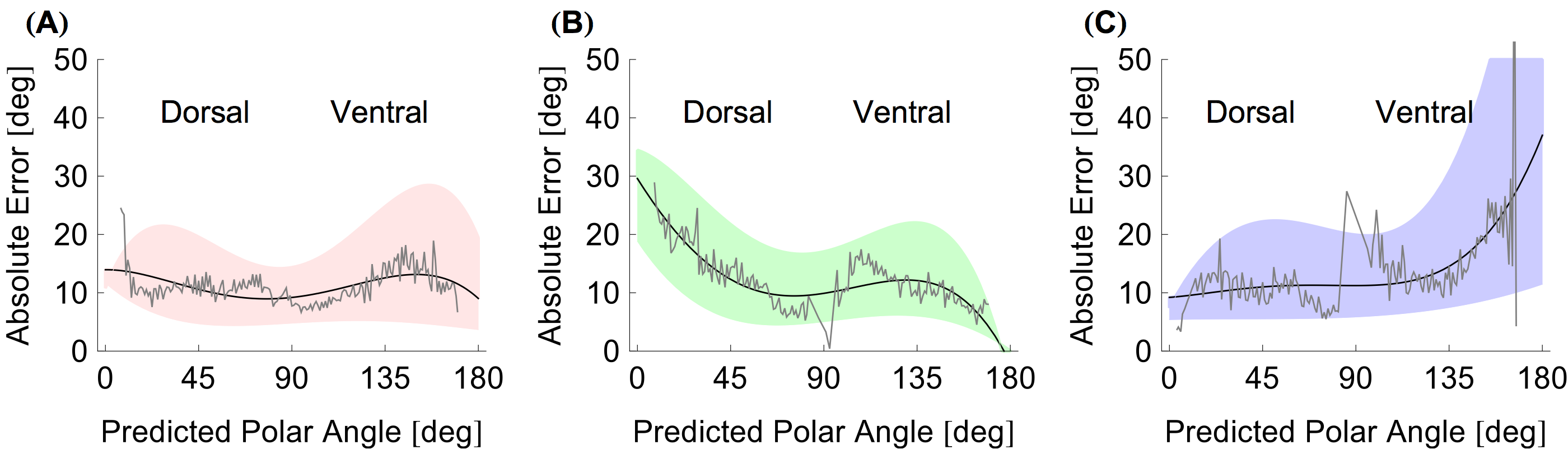

Supplement: Figure S1 — Polar angle prediction error for (A) V1, (B) V2, and (C) V3. In each figure, the gray line shows the median absolute leave-one-out error for vertices based on their predicted polar angle. The black line shows a best-fit fifth order polynomial to the median. The shaded regions show the extent of the upper and lower quartile of the errors. A spike in median absolute error can be seen near 90° of polar angle in both V2 and V3; this spike is due to error in a region near the foveal confluence (Fig. S3). (TIF) [file pcbi.1003538.s001.tif]

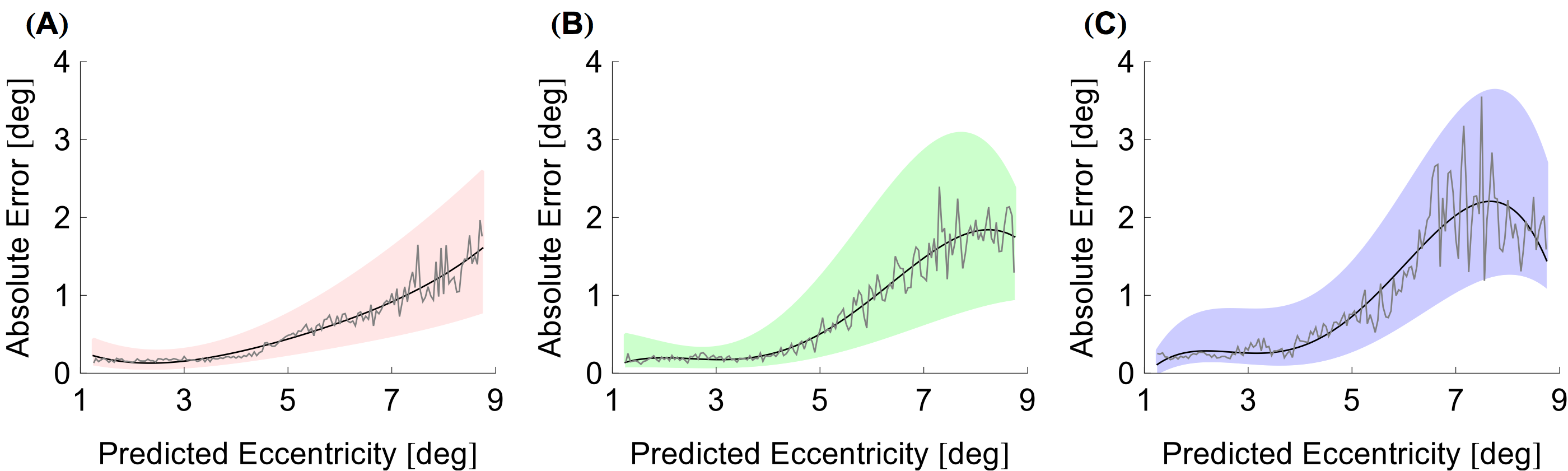

Supplement: Figure S2 — Eccentricity prediction error for (A) V1, (B) V2, and (C) V3. In each figure, the gray line shows the median absolute leave-one-out error for vertices based on their predicted polar angle. The black line shows a best-fit fifth order polynomial to the median. The shaded regions show the extent of the upper and lower quartile of the errors. (TIF) [file pcbi.1003538.s002.tif]

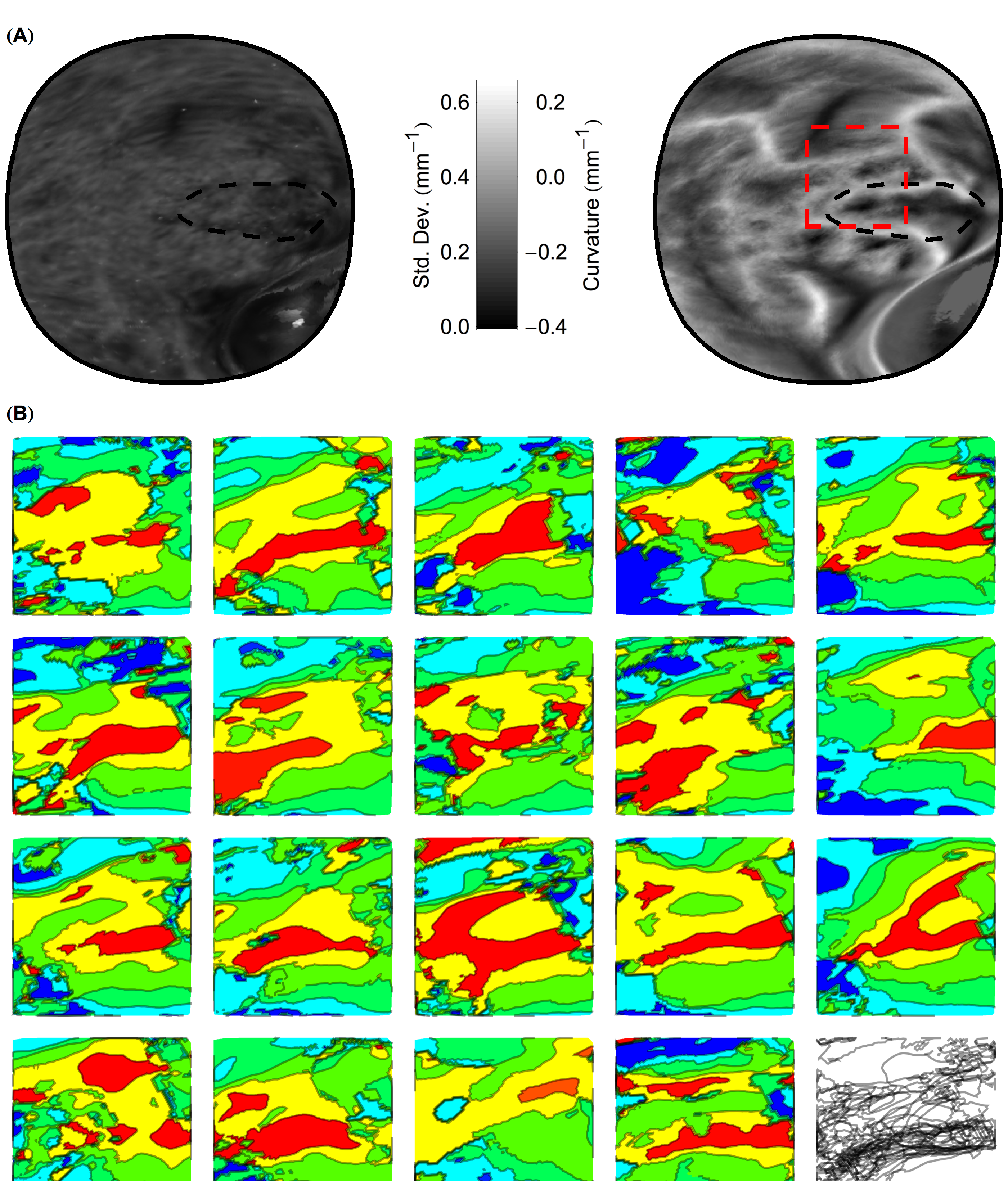

Supplement: Figure S4 — Exploration of the dorsal V3/V3A border. (A) The standard deviation (left) and median (right) sulcal curvature across all subjects and hemispheres. The dashed red outline indicates the region from which plots in B are taken. (B) Contour plots of polar angle for all subjects' dorsal V2/V3/V3A regions. Contour lines are drawn at 0°, 30°, 60°, 90°, 120°, 150°, and 180°. In the lower right corner, the 150° contour lines for all subjects are shown together. Although the V1/V2 border reversal is relatively conserved, much less agreement can be found for the V3/V3A reversal. (TIF) [file pcbi.1003538.s004.tif]
