# Supplementary material for: Correction of Distortion in Flattened Representations of the Cortical Surface Allows Prediction of V1-V3 Functional Organization from Anatomy
Source: PLoS Comput Biol. 2014 Mar 27;10(3):e1003538. doi: 10.1371/journal.pcbi.1003538 (PMC3967932; doi:10.1371/journal.pcbi.1003538)
Supplement: File S1 — Supplemental Mathematica Notebook, rendered as a PDF file, containing source code and tools for the analysis of registered cortical surfaces. A version of this file in Mathematica's native notebook format (.nb file) is available for download from our lab website: https://cfn.upenn.edu/aguirre/wiki/public:data_ploscomputbiol_2014_benson. (PDF) [file pcbi.1003538.s005.pdf]

Extrastriate Cortex Retinotopic Maps

Noah C. Benson[1,2], Omar H. Butt[1], David H. Brainard[2], and Geoffrey K. Aguirre[1]

Departments of (1) Neurology and (2) Psychology
University of Pennsylvania
Philadelphia, PA 19104

A version of this notebook in *Mathematica*'s format (.nb file) is available for download from our lab website:
https://cfn.upenn.edu/aguirre/wiki/public:data_ploscomputbiol _ 2014_benson.
The full source code of our spring embedding program can be found on gitHub:
https://github.com/noahbenson/SpringRegister.

---

1. Introduction

This *Mathematica* notebook is designed to provide a tutorial and analysis tool for studying the organization of retinotopic maps in human V1, V2, and V3. The Introduction section primarily includes code and dependencies that are needed by the rest of the notebook. It should be evaluated entirely before further analysis is performed.
Note that this notebook uses a lazy programming style throughout. This means that the introduction will evaluate quite quickly, despite the fact that it should be loading and processing lots of data. What actually happens is that the introduction provides instructions to *Mathematica* that are saved and evaluated when first needed then cached for all subsequent use. This means that the first time you try to look at data or make a figure after evaluating the Introduction, it will seem to take a long time; the next time you do the same thing, however, it should run much more quickly.
The configuration section of the introduction is the only section that really needs to be edited prior to use of the rest of the notebook.

1.1. Configuration

The $ExtrastriateDirectory variable tells the rest of this notebook where to find data. By default this should point to our website where you can download the data over the Internet. If you would like faster load times, you may download the data tarball from our wiki and unzip it locally then point this directory to the unzipped path.

```
$ExtrastriateDirectory = "http://cfn.upenn.edu/aguirreg/public/ES_template";
```

These do not need to be changed unless you have edited the structure of these directories.

```
$DataDirectory = $ExtrastriateDirectory <> "/data";
$MetadataDirectory = $ExtrastriateDirectory <> "/metadata";
$SubjectDirectory = $ExtrastriateDirectory <> "/data/subjects";
$SubjectFile = $SubjectDirectory <> "/subjects.txt";
$OutputDirectory = $ExtrastriateDirectory <> "/outputs";
```

If you are running your own spring simulations, these may be edited to specify where you store the spring simulations and simulation data. If you are using our gitHub repository to examine our data, then the $SpringsDirectory variable should be set to the gitHub root.

```
$SpringsDirectory = "~/Documents/Extrastriate/springs";
$SpringsJobsDirectory = $SpringsDirectory <> "/jobs";
$SpringsResultsDirectory = $SpringsDirectory <> "/results";
```

If you want to export your figures to a single directory, this directory will specify where figure outputs get written.

```
$FiguresOutputDirectory = "~/Desktop";
```

1.2. Dependencies

These packages are built-in *Mathematica* packages, but they must be declared before used.

```
Needs["ErrorBarPlots`"];
Needs["MultivariateStatistics`"];
Needs["ComputationalGeometry`"];
```

1.3. Utilities

1.3.1. Local Keywords

This is a utility function that allows us to declare constant keyword-like symbols in *Mathematica*. If you are not deeply familiar with *Mathematica*'s parsing language; do not worry too much about this or its use; just don't poke it too much.

```
Clear[DeclareKeyword];
DeclareKeyword::badsym = "Argument `1` is neither a rule nor a symbol";
DeclareKeyword[syms_List] := Scan[
   Function[{arg},
    Which[
     Head[arg] === Symbol, (
      Unprotect[Evaluate[arg]];
      arg = arg;
      Protect[Evaluate[arg]]),
     Head[arg] === Rule && Head[First[arg]] === Symbol, (
      Unprotect[Evaluate[First[arg]]];
      Evaluate[First[arg]] = First[arg];
      ToString[First[arg]] ^= Last[arg];
      Protect[Evaluate[First[arg]]]),
     True, Message[DeclareKeyword::badsym, arg]],
    {HoldFirst}],
   syms];
DeclareKeyword[syms__] := DeclareKeyword[{syms}];
Attributes[DeclareKeyword] = {HoldAll};
```

These are a few keywords used very commonly in this notebook.

```
DeclareKeyword[
  LH, RH,
  Eccentricity, PolarAngle, FStat,
  Hemisphere];
```

1.3.2. Error Handling

This function prints an error message and generates an Abort[] when things don't add up. It can be used to check progress for sanity. The ifTrue and errorMessage arguments are evalueated only when required.

```
SanityCheck[value_, test_, errorMessage_] := If[test[value],
   value,
   (Print[errorMessage]; Abort[])];
```

This function prints an error message (not using *Mathematica*'s Message[] function), then Abort[]'s

```
Die[msg_] := (Print[msg]; Abort[]);
```

1.3.3. Reading VTK Files

Mathematica natively reads VTK file vertex and polygon data, but does not read the field data. This function will do this, and return it as a list of Rules.

```
ReadVTK[filename_String, OptionsPattern[]] := Module[
   {fl = StringToStream[
      (* we do all this instead of a normal
       stream because only import correctly handles URLs *)
      StringJoin @@ Import[
        filename,
        "Character8"]], (* The stream for this particular file *)
    p, n, m, dat, (* used below as temporaries *)
    toSpherical = SanityCheck[OptionValue[ConvertToSpherical],
      # == True || # == False &,
      "ConvertToSpherical option to ReadVTK must be True or False"],
    unmask = SanityCheck[OptionValue[UnmaskData],
      # == True || # == False &,
      "UnmaskData option to ReadVTK must be True or False"]},
   dat = Reap[
      For[p = Find[fl, "FIELD"], ! (p === EndOfFile), p = Find[fl, "Field"],
       {p, n, m, p} = Read[fl, {Word, Number, Number, Word}];
       Sow[Partition[ReadList[fl, Number, n * m], n]]];
      Close[fl]][[2, 1]];
   dat = Thread[
     Import[filename, "VertexData"] → Map[Flatten, Transpose[dat]]];
   (* At this point, dat is the data in the form of a list of {x,y,z}→
    value rules. We may want to clean these up according to
     the options: if the "ConvertToSpherical" option is true,
   we convert everything into spherical coordinates.  If the
    "UnmaskData" option is true, we filter out 0-valued
     data members. *)
   Which[
    (* We want to trim out those elements of dat that have zero values
     and convert the coordinates to spherical coordinates*)
    unmask && toSpherical, Flatten[
     Reap[
       Replace[dat, ({x_, y_, z_} → {value_}) :→
         If[value ≠ 0., Sow[CartesianToSpherical[{x, y, z}]]], {1}]
      ][[2]],
     1],
    (* We want to unmask the
     data but not convert it into spherical coordinates *)
    unmask, Flatten[
     Reap[
       Replace[dat, (coord_ → {value_}) :→ If[value ≠ 0., Sow[coord]], {1}]
      ][[2]],
     1],
    (* We want to convert to spherical
     coordinates but keep the values and not filter *)
    toSpherical, CartesianToSpherical[dat],
    (* or we just leave dat alone and return it *)
    True, dat]];
ReadVTK[__] := Die["Error: ReadVTK[] must be called with a filename"];
(* These options allow ReadVTK to unmask
 data and/or convert it to spherical coordinates *)
Options[ReadVTK] = {ConvertToSpherical → False, UnmaskData → False};
DeclareKeyword[ConvertToSpherical, UnmaskData];
```

1.3.4. Handy General Functions

This function normalizes the given value such that the result is between 0 and 1 and is 0 if the value <= min and is 1 if the value is >= max.

```
ScaleOrTruncate[val_, {min_, max_}] := Which[
   val < min, 0,
   val > max, 1,
   True, (val - min) / (max - min)];
```

This very useful function will take a list of subjects' data (as stored in the datastructures defined below) and transpose across vertices. Ie, Merge[sub1, sub2, sub3] will yield a list whose ith element is {xi,yi}→{{sub1[[3]], sub2[[3]], sub3[[3]]}, {sub1[[4]], sub2[[4]], sub-3[[4]]}, ...}. Any time a subject is not represented at the point (ie, does not have an element matching {xi,yi,__}), None is used in place of that subject's value.

```
Merge[data : {_List ..}, OptionsPattern[]] := With[
   {idf = OptionValue[GatherBy],
    sowf = OptionValue[Sow],
    ϵ = Replace[
      OptionValue[Precision],
      {x_Integer /; x > 0 ⧴ x,
       _ ⧴ Die["Precision must be a positive integer"]}],
    fillf = OptionValue[Filling],
    selectf = OptionValue[Select],
    resolvef = OptionValue[Resolve],
    composef = OptionValue[Compose],
    n = Length@data},
   Last[
    Reap[
     MapIndexed[
      Function[{datum, index},
       If[selectf[datum], Sow[index[[1]] → sowf[datum], {idf[datum]}]]],
      data,
      {2}],
     _,
     Function[{id, sowed},
      Print[{id, sowed}];
      composef[
       id,
       Map[
        {idx} Ì If[idx == 0, None, sowed[[idx, 2]]],
        SparseArray[
         MapIndexed[{rule, idx} Ì Rule[rule[[1]], idx[[1]]], sowed],
         {n},
         0]]]]]]];
Options[Merge] = {
   GatherBy → (#[[1 ;; 2]] &),
   Sow → (#[[3 ;; All]] &),
   Precision → 4,
   Filling → None,
   Select → (True &),
   Resolve → First,
   Compose → Rule};
```

1.4. Geometry Functions

1.4.1. Convert to Spherical Coordinates

These functions will convert 3D coordinates into spherical coordinates assuming that the data lies on a sphere and we don’t need to r/ρ coordinate (as we generally assume in this file with spherical brains). These also automatically handle lists of coord→value rules since we deal with them frequently while reading in VTK files.

```
CartesianToSpherical[dat : {{_, _, _} ..}] := Map[
   {If[#[[1]] == 0. && #[[2]] == 0., 0, ArcTan[#[[1]], #[[2]]]],
     ArcSin[#[[3]] / Norm[#]]} &,
   dat];
CartesianToSpherical[dat : {Rule[{_, _, _}, _] ..}] := Map[
   Rule[
     {If[#[[1, 1]] == 0. && #[[1, 2]] == 0., 0, ArcTan[#[[1, 1]], #[[1, 2]]]],
      ArcSin[#[[1, 3]] / Norm[#[[1]]]]},
     #[[2]]] &,
   dat];
CartesianToSpherical[dat : {_, _, _}] := List[
   If[dat[[1]] == 0. && dat[[2]] == 0.,
    0,
    ArcTan[dat[[1]], dat[[2]]]],
   ArcSin[dat[[3]] / Norm[dat]]];
CartesianToSpherical[dat : Rule[{_, _, _}, _]] := Rule[
   {If[dat[[1, 1]] == 0. && dat[[1, 2]] == 0., 0, ArcTan[dat[[1, 1]], dat[[1, 2]]]],
    ArcSin[dat[[1, 3]] / Norm[dat]]},
   dat[[2]]];
CartesianToSpherical[dat : {Rule[{_, _, _}, _] ..}] := Map[
   Rule[
     {If[#[[1, 1]] == 0. && #[[1, 2]] == 0., 0, ArcTan[#[[1, 1]], #[[1, 2]]]],
      ArcSin[#[[1, 3]] / Norm[#[[1]]]]},
     #[[2]]] &,
   dat];
```

1.4.2. V1 Region

There must be a definition of V1 in order for this file to work properly. V1 should be an n x 2 matrix in which each row is the coordinate of a vertex in V1.

```
$V1Filename = $ExtrastriateDirectory <> "/metadata/V1-predict.mh.vtk";
```

The definition for V1; there is an untransformed and a transformed version. Both should be lists containing only the points in V1, but the untransformed one will be .

```
V1Untransformed := Set[
   V1Untransformed,
   ReadVTK[
    $V1Filename,
    UnmaskData → True,
    ConvertToSpherical → False]];
V1 := (V1 = V1Transform[V1Untransformed]);
```

```
$OccipitalPole := Set[
   $OccipitalPole,
   {-V1EllipseA, 0}];
```

Frequently we also want to plot the V1 convex hull (outline); this can be done with this variable...

```
V1Hull := (V1Hull = V1[[Append[#, First@#]]] &[ConvexHull[V1]]);
```

The Expanded V1 is similar to V1 but should include points that surround V1; we use the OP Hinds et al. probabilistic V1 definition for this. It is assumed that V1Expanded is

loaded by the same method V1, but this does not have to be true.

```
$V1ExpandedFilename = $MetadataDirectory <> "/V1-prob.mh.vtk";
V1ExpandedUntransformed := Set[
   V1ExpandedUntransformed,
   ReadVTK[
    $V1ExpandedFilename,
    UnmaskData → True,
    ConvertToSpherical → False]];
V1Expanded := (V1Expanded = V1Transform[V1ExpandedUntransformed]);
```

1.4.3. FSAverage Conversion to a 2D Map

Freesurfer's FSAverage spherical brain has some quirks when it comes to the anatomical shape of V1; we correct for some of this here using a shear transformation. Note that you won't want to use this (e.g., in your data-loading functions) if you are not using the FSAverage sphere.

```
$FSAverageShearMatrix = {{1, 0.65}, {0, 1}};
```

The full transformation matrix for converting (ie, shearing then rotating to (0,0)) a point on the fsaverage sphereical surface to a point on a 2D map.

```
$FSAverageTransformMatrix = $FSAverageShearMatrix;
```

Finally, we need a transform that rotates the Cartesian coordinates such that the point we want is at the (0,0) point on the sphere, which we take to be (1,0,0) in Cartesian coordinates.

```
$CartesianSphereRotationMatrix := Set[
   $CartesianSphereRotationMatrix,
   RotationMatrix[
    {Mean[V1Untransformed] / Norm[Mean[V1Untransformed]],
     {1, 0, 0}}]];
```

This function implements the above transform

```
FSSphereToMap[dat : {_List ..}] := Map[
   Function[
    Join[
     Dot[
      $FSAverageTransformMatrix,
      CartesianToSpherical[$CartesianSphereRotationMatrix.#[[1 ;; 3]]]],
     #[[4 ;; All]]]],
   dat];
FSSphereToMap[dat : {Rule[_List, _List] ..}] := Map[
   Function[
    Join[
     Dot[
      $FSAverageTransformMatrix,
      CartesianToSpherical[$CartesianSphereRotationMatrix.#[[1]]]],
     #[[2]]]],
   dat];
FSSphereToMap[dat : {Rule[_List, _?NumberQ] ..}] := Map[
   Function[
    Append[
     Dot[
      $FSAverageTransformMatrix,
      CartesianToSpherical[$CartesianSphereRotationMatrix.#[[1]]]],
     #[[2]]]],
   dat];
```

This is just a handy function for calculating distance along the surface of the sphere

```
SphericalDistance[{θ1_, ϕ1_}, {θ2_, ϕ2_}] :=
  ArcCos[Sin[ϕ1] * Sin[ϕ2] + Cos[ϕ1] * Cos[ϕ2] * Cos[θ1 - θ2]];
```

Sometimes, we want to look at just the region of the brain relatively close to the occipital pole. Here, we define a function that will select only the vertices close to the OP.

```
Options[SelectNearbyVertices] := {
   DistanceCutoff → (Pi / 3),
   OP → Automatic};
DeclareKeyword[DistanceCutoff, OP];
SelectNearbyVertices[data_List, OptionsPattern[]] := With[
   {op = Replace[
      OptionValue[OP],
      {coord : {x_?NumberQ, y_?NumberQ} :→ coord,
       Automatic :→ $OccipitalPole,
       _ :→ Die["Invalid OP argument"]}],
    dcutoff = Replace[
      OptionValue[DistanceCutoff],
      {x_ /; And[NumberQ[N@x], x > 0] :→ x,
       x_ :→ Die["DistanceCutoff must be a positive number: " <> ToString[x]]}]},
   With[
    {sϕ = Sin[op[[2]]],
     cϕ = Cos[op[[2]]],
     θ = op[[1]]},
    Select[
     data,
     {tp} ↦
      (ArcCos[sϕ * Sin[tp[[2]]] + cϕ * Cos[tp[[2]]] * Cos[θ - tp[[1]]]] < dcutoff)]]];
```

1.4.4. The “Local” Region and Anatomical Data

Here, we define the variables containing anatomical data for the FSAverage-sym hemisphere.

```
$AnatomyFilename = $MetadataDirectory <> "/anatomy/fs-average-template.vtk";
FullAnatomy := Set[
   FullAnatomy,
   V1Transform[
    Map[
     {rule} Ì Apply[Join, rule],
     ReadVTK[
      $AnatomyFilename,
      UnmaskData → False,
      ConvertToSpherical → False]]]];
```

The LocalAnatomy variable is the same as the FullAnatomy variable, but it is within the “Local” region only; this is defined as the region within Pi/3 rad of the occipital pole (OP).

```
LocalAnatomy := Set[
   LocalAnatomy,
   SelectNearbyVertices[FullAnatomy]];
```

The LocalHull is just a polygon that contains all the points in the “local” region (points near the OP).

```
LocalHull := Set[
   LocalHull,
   With[{hull = ConvexHull[LocalAnatomy[[All, 1 ;; 2]]]},
    LocalAnatomy[[Append[hull, First@hull], 1 ;; 2]]]];
```

NormAnatomy is a normalized version of LocalAnatomy.

```
NormAnatomy := Set[
   NormAnatomy,
   With[
    {mn = Min[LocalAnatomy[[All, 3]]],
     mx = Max[LocalAnatomy[[All, 3]]]},
    Map[
     {#[[1]], #[[2]], 1 - 2 * (#[[3]] - mn) / (mx - mn)} &,
     LocalAnatomy]]];
```

1.4.5. Coordinate Axes and V1 Ellipse

Once data has been loaded, we want to be able to define a coordinate system that places the V1 ellipse around the origin with the major axis as the x-axis and the minor axis as the y-axis. To do this, we want to first fit the V1 ellipse to the V1 data then rotate and translate all of the imported data to coincide with this. In order to do this, we must first convert the V1 data into surface spherical coordinates--we would like to rotate it around so that the center of V1 is at (0°,0°) to assure that there is minimal spherical distortion in our projection. We then find a fit for the V1 ellipse that minimizes the number of points not in V1 inside the ellipse and maximizes the number of points in V1 inside the ellipse. We can do this by fitting an elliptical 2D Plateau to a rotated/transposed V1 and calling the ellipse

border the curve where plateau(x,y) = 0.5.
For a plateau function, we use a modified (stretched/rotated) form of the sigmoid function 1/(1+exp(-t)) that has been rotated around the origin; we use a fixed gain (a high gain means the sigmoid has a very high derivative at 0).

```
V1SigmoidModel := Set[
   V1SigmoidModel,
   Block[{θ, x0, y0, σx, σy, x, y},
    With[
     {region = Join[
        FSSphereToMap[Append[#, 1.0] & /@ V1Untransformed],
        FSSphereToMap[
         Append[#, 0.0] & /@ Complement[
           V1ExpandedUntransformed,
           V1Untransformed]]],
      plateau = Function[{pt},
        1 - 1 / (1 +
            Exp[-20 *
              (Sqrt[σx * ((pt[[1]] - x0) * Cos[θ] + (pt[[2]] - y0) * Sin[-θ]) ^2 +
                  σy * ((pt[[1]] - x0) * Sin[θ] + (pt[[2]] - y0) * Cos[θ]) ^2] - 2)])]},
     (* Note that form uses an inverse rotation;
     this way θ will end up being the proper value for rotating the points
      in V1 rather than for rotating the Gaussian to the points.  We
      rotate/translate the Plateau during fitting in order to speed things up,
     but in the end we want to rotate/translate the points themselves. *)
     NonlinearModelFit[
      region,
      {1 - 1 / (1 + Exp[-20 * (Sqrt[σx * ((x - x0) * Cos[θ] + (y - y0) * Sin[-θ]) ^2 +
                 σy * ((x - x0) * Sin[θ] + (y - y0) * Cos[θ]) ^2] - 2)]),
       σy > 0 && σx > 0},
      {{x0, Mean[region[[All, 1]]]},
       {y0, Mean[region[[All, 2]]]},
       {θ, -Pi / 8},
       {σx, 5},
       {σy, 20}},
      {x, y},
      AccuracyGoal → 4]]]];
```

If we wish, we can inspect the V1SigmoidModel to see what kind of fit we found. This forces evaluation of the fit, so in the interest of good lazy programming, it is commented out. The values that were fit when I ran it were:
{x0→-0.00712698,y0→-0.0013869,θ→-0.293164,σx→18.1387,σy→148.343}

```
(*V1SigmoidModel["BestFitParameters"]*)
```

```
V1SigmoidPlot := Show[
   DensityPlot[
    V1SigmoidModel[x, y],
    {x, -1, 1},
    {y, -1, 1},
    ColorFunction → (Blend[{Blue, Cyan, Green, Yellow, Red}, #] &),
    PlotRange → Full,
    Frame → False],
   ListPlot[
    Part[
     FSSphereToMap[V1Untransformed],
     Append[#, First@#] &@ConvexHull[FSSphereToMap@V1Untransformed]],
    PlotStyle → {Black, Dashed, Thick},
    Joined → True,
    ImageSize → 3.25 * 72,
    Frame → False]];

(*V1SigmoidPlot*)
```

Now that we have the model for the sigmoid, we can untranslate and unrotate all of the points in V1 by building a transformation (at which point V1 and all sets of spherical points already loaded will automatically translate themselves to our coordinate system).

The V1 transform must translate a point by (x0,y0) then must rotate a point by θ degrees. If this seems wrong, it’s because we built the plateau with the wrong rotation formula, so it actually rotates -θ.

```
V1SigmoidModelX0 := Set[
   V1SigmoidModelX0,
   Block[{x0, y0}, {x0, y0} /. V1SigmoidModel["BestFitParameters"]]];
V1SigmoidModelRotationMatrix := Set[
   V1SigmoidModelRotationMatrix,
   Block[{θ}, RotationMatrix[θ /. V1SigmoidModel["BestFitParameters"]]]];

V1Transform[dat_List] := SortBy[
   Map[
    Join[
      Dot[
       V1SigmoidModelRotationMatrix,
       #[[1 ;; 2]] - V1SigmoidModelX0],
      #[[3 ;; All]]] &,
    FSSphereToMap[dat]],
   #[[1 ;; 2]] &];
```

Now, we would like the elliptical form of this model; we can solve the equation for values of 0.5 to find this particular equation.
Notably, since we want to find the ellipse parameters for the data post-rotation, what we really need at this point is to drop the x0, y0, and θ parameters, so this becomes much simpler.

```
(* Notably, we can use substitution to first show that Sqrt[σx*x^2+σy*y^2]==2;
this is our ellipse equation *)
V1EllipseEquation := Block[{σx, σy, x, y, u},
   V1EllipseEquation = Equal[
     σx * x^2 + σy * y^2 /. V1SigmoidModel["BestFitParameters"],
     u /. FindRoot[
       1 - 1 / (1 + Exp[-20 * (Sqrt[u] - 2)]) == 0.5,
       {u, 2}]]];

(* A similar technique gives us the Dialated
 ellipse (V1 ellipse with twice the radius) *)
V1DialatedEllipseEquation := Block[{σx, σy, x, y, u},
   V1DialatedEllipseEquation = Equal[
     σx * x^2 + σy * y^2 /. V1SigmoidModel["BestFitParameters"],
     2 * u /. FindRoot[
       1 - 1 / (1 + Exp[-20 * (Sqrt[u] - 2)]) == 0.5,
       {u, 2}]]];
```

Again, we can examine this if we wish to know the equation, but this will force evaluation. When I ran it, the result was:

$18.1387 x^2 + 148.343 y^2 == 4.$

```
(*V1EllipseEquation*)
```

1.4.6. Test if a Point is in V1 or Other Regions

Using the equations and variables defined above, we can declare the V1 ellipse/test functions and inequalities...

```
InV1EllipseEquation := Block[{x, y},
   InV1EllipseEquation = Apply[LessEqual, V1EllipseEquation]];

InV1Q[a : {_, _, __}] :=
  (InV1Q[{x_, y_, rest__}] := Evaluate[InV1EllipseEquation]; InV1Q[a]);
InV1Q[a : {_, _}] := (InV1Q[{x_, y_}] := Evaluate[InV1EllipseEquation]; InV1Q[a]);
InV1Q[x0_, y0_] := (InV1Q[x_, y_] := Evaluate[InV1EllipseEquation]; InV1Q[x0, y0]);
```

We can also setup a similar set of equations/functions for the dialated V1 regions (see previous section; this is an ellipse that has twice the radius as the V1 ellipse)

```
InV1DialatedEllipseEquation := Module[
   {v1a = Block[{x, y}, FindRoot[V1EllipseEquation /.y → 0, {x, 1.0}][[1, 2]]],
    v1b = Block[{x, y}, FindRoot[V1EllipseEquation /.x → 0, {y, 1.0}][[1, 2]]]},
   Block[{x, y},
    InV1DialatedEllipseEquation = ((x / v1a)^2 + (y / v1b)^2 ≤ 2)]];
InV1DialatedQ[a : {_, _, __}] := (InV1DialatedQ[{x_, y_, rest__}] :=
    Evaluate[InV1DialatedEllipseEquation]; InV1DialatedQ[a]);
InV1DialatedQ[a : {_, _}] := (InV1DialatedQ[{x_, y_}] :=
    Evaluate[InV1DialatedEllipseEquation]; InV1DialatedQ[a]);
InV1DialatedQ[x0_, y0_] := (InV1DialatedQ[x_, y_] :=
    Evaluate[InV1DialatedEllipseEquation]; InV1DialatedQ[x0, y0]);
```

Finally, we want some access to the parameters of the ellipse; most of these are pretty simple, but they are useful nonetheless

```
V1EllipseA := (V1EllipseA =
    Block[{x, y}, FindRoot[V1EllipseEquation /.y → 0, {x, 1.0}][[1, 2]]]);
V1EllipseB := (V1EllipseB = Block[{x, y},
     FindRoot[V1EllipseEquation /.x → 0, {y, 1.0}][[1, 2]]]);
V1EllipseX0 = 0;
V1Ellipsey0 = 0;
V1Ellipseθ = 0;
```

```
V1DialatedEllipseA := (V1DialatedEllipseA =
    Block[{x, y}, FindRoot[V1DialatedEllipseEquation /.y → 0, {x, 1.0}][[1, 2]]]);
V1DialatedEllipseB := (V1DialatedEllipseB =
    Block[{x, y}, FindRoot[V1DialatedEllipseEquation /.x → 0, {y, 1.0}][[1, 2]]]);
V1DialatedEllipseX0 = 0;
V1DialatedEllipsey0 = 0;
V1DialatedEllipseθ = 0;
```

1.4.7. Testing to see if a point is in a polygon

```
With[{ϵ = 2.0 * $MachineEpsilon},
  PointInPolygonQ[{x_, y_}, polygon_, OptionsPattern[]] := Condition[
    With[
     {pgon = Map[
        (# - {x, y}) &,
        N[If[Last[polygon] == First[polygon],
          polygon,
          Append[polygon, First[polygon]]]]],
      incl = OptionValue[BoundaryStyle]},
     Switch[
      Apply[
       Times,
       MapThread[
        (* we're looking for intercepts on the ray that is the -y axis *)
        Function[{a, b},
         With[{intercept = a[[2]] - a[[1]] * (a[[2]] - b[[2]]) / (a[[1]] - b[[1]])},
          Which[
           Abs[a[[1]] - b[[1]]] < ϵ,
           If[Abs[a[[1]]] > ϵ || Sign[a[[2]]] == Sign[b[[2]]], 1, 0],
           Abs[intercept] < ϵ, 0,
           Sign[a[[1]]] == Sign[b[[1]]], 1,
           True, Sign[intercept]]]],
        {Most[pgon], Rest[pgon]}]],
      (* Outside the polygon (even number of crossings) *)
      _?Positive, False,
      (* Inside the polygon (odd number of crossings) *)
      _?Negative, True,
      (* point is a vertex or on a boundary *)
      _, incl]],
    And[NumberQ[x], NumberQ[y], ListQ[polygon], MatchQ[Dimensions[polygon],
      {_, 2}], Count[polygon, _?NumberQ, 2] == 2 * Length[polygon]]];
  Options[PointInPolygonQ] = {BoundaryStyle → Indeterminate}];
```

1.5. Analysis and Plotting-Related Functions

1.5.1. Color Scale with Truncation

TruncatedColorScale is a function that yields a function for passing as a ColorFunction argument.

```
TruncatedColorScale[val_, {min_, max_},
   colors_: Automatic, OptionsPattern[]] := With[
   {clrs = Which[
      colors === Automatic, {Blue, Cyan, Green, Yellow, Red},
      colors === GrayLevel, {Black, White},
      ! ListQ[colors], Die["Bad colors argument to TruncateColorScale"],
      True, colors]},
   Which[
    val < min, OptionValue[BelowColor] /. Automatic → First[clrs],
    val > max, OptionValue[AboveColor] /. Automatic → Last[clrs],
    True, Blend[clrs, (val - min) / (max - min)]]];
Options[TruncatedColorScale] = {BelowColor → LightGray, AboveColor → LightGray};
DeclareKeyword[BelowColor, AboveColor];
```

1.5.2. Aggregate the Errors from Observed Data versus a Model Prediction

This function takes a dataset token (like data10), a simulation prediction (like sim10[S-moothPrediction]), and an eccentricity range; it yields the errors in that eccentricity range between the predicted model and the dataset’s aggregate.

```
AggregateErrors[data_, simmdl_, {minEc_, maxEc_}, minF_] := With[
   {dat = Sort[data[Aggregate], OrderedQ[{#1[[1 ;; 2]], #2[[1 ;; 2]]}] &],
    mdl = Map[
      Function[{x},
       Map[{#[[1]], #[[2]], x[[2]][#[[3]]]} &,
        Sort[x[[1]], OrderedQ[{#1[[1 ;; 2]], #2[[1 ;; 2]]}] &]]],
      {MergeScales[simmdl[[All, 1 ;; 3]] → (# &), LocalAnatomy → (False &)],
       MergeScales[simmdl[[All, {1, 2, 4}]] → (# &), LocalAnatomy → (False &)],
       MergeScales[simmdl[[All, {1, 2, 5}]] → (# &), LocalAnatomy → (False &)]}]},
   Reap[
     MapThread[
      If[#1[[5]] > minF && minEc ≤ #1[[4]] ≤ maxEc && NumberQ[#2[[3]]],
        Sow[#1[[1 ;; 4]] → Append[#2, #3[[3]]], {0, Round[#4[[3]]]}]] &,
      {dat, mdl[[1]], mdl[[2]], mdl[[3]]}],
     {0, 1, 2, 3},
     Apply[Sequence, #2] &
    ][[2]]];
```

This makes a report out of the errors gathered by the above function.

```
ReportAggregateErrors[errors_] := With[
   {pa = Transpose /@
      Map[{#[[1, 3]] - #[[2, 3]], Abs[#[[1, 3]] - #[[2, 3]]]} &, errors, {2}],
    ec = Transpose /@ Map[{#[[1, 4]] - #[[2, 4]], Abs[#[[1, 4]] - #[[2, 4]]]} &,
       errors, {2}]},
   {PolarAngle → MapIndexed[(If[#2[[1]] == 1, All, #2[[1]] - 1] → Median /@ #1) &, pa],
    Eccentricity →
     MapIndexed[(If[#2[[1]] == 1, All, #2[[1]] - 1] → Median /@ #1) &, ec]}];
```

1.5.3. Collect Leave-One-Out Error Data for Analysis

This is just a self-memoizing function; ie, if you refer to it with a particular argument set, it will hash itself and not require calculation after the first reference. The data argument should be a dataset, like data10, and the looSims should be a list of leave-one-out sim predictions, such as (#[SmoothPrediction]&/@simsLOO).

```
Clear[CollectLOOErrors];
CollectLOOErrors[data_, looSims_List, {eccenMin_, eccenMax_}] := With[
    {fStatMin = data[FStatRange][[1]]},
    Reap[
      MapThread[
       Function[{sp, sub},
        With[
         {dat = SortBy[sub, #[[1 ;; 2]] &],
          temp = Map[
            Function[{x},
             {x[[2]], SortBy[x[[1]], #[[1 ;; 2]] &][[All, 3]]}],
            {MergeScales[sp[[All, 1 ;; 3]] → (# &), LocalAnatomy → (False &)],
             MergeScales[sp[[All, {1, 2, 4}]] → (# &), LocalAnatomy → (False &)],
             MergeScales[sp[[All, {1, 2, 5}]] → (# &), LocalAnatomy → (False &)]}]},
         With[
          {fns = temp[[All, 1]],
           tmp = temp[[All, 2]]},
          MapThread[
           Function[{pa, ec, area, full},
            If[
             And[
              NumberQ[pa], NumberQ[ec], NumberQ[area], NumberQ[full[[5]]],
              full[[5]] ≥ fStatMin,
              0 < Abs[area] < 4, eccenMin < ec ≤ eccenMax, 0 < pa ≤ 180.0],
             Sow[
              {{pa, full[[3]]}, {ec, full[[4]]}, area},
              {full[[1 ;; 2]]}]]]],
           Append[
            MapThread[
             Function[{d, fn}, fn /@ d],
             {tmp, fns}],
            dat]]]]],
       {looSims, data[Data, All]}],
      _,
      Rule
     ][[2]]
   ] /; And[eccenMin ≥ 0, eccenMax > eccenMin];
```

This is a bit of trickery that allows the results of this function to be explored and cached in association to a dataset key; its's essentially a method injection, but for all intents and purposes, you can, because of this block of code, call things like:
data10[CollectLOOErrors, simsLOO, SmoothPredictionVoronoi, {1.25,8.75}]
and have it memoize the results.

```
CollectLOOErrors /: data_[CollectLOOErrors, sims_List, {eccMn_, eccMx_}] := Set[
   data[CollectLOOErrors, sims, {eccMn, eccMx}],
   CollectLOOErrors[data, sims, {eccMn, eccMx}]];
```

1.5.4. Report on Collected Leave-One-Out Error Data

This funciton generates an easily readable matrix of the LOO errors collected via the functions in 1.5.3.

```
ReportLOOErrors[data_, sims_List, {eccMn_, eccMx_}] := With[
   {looErr = data[CollectLOOErrors, sims, {eccMn, eccMx}]},
   With[
    {eccerrTmp = Reap[
        Scan[
         Sow[#[[1]], {0, Round@#[[2]]}] &,
         Flatten[looErr[[All, 2, All, 2 ;; 3]], 1]],
        {0, 1, 2, 3},
        Apply[Sequence, #2] &
       ][[2]],
     polerrTmp = Reap[
        Scan[
         Sow[#[[1]], {0, Round@#[[2]]}] &,
         Flatten[looErr[[All, 2, All, {1, 3}]], 1]],
        {0, 1, 2, 3},
        Apply[Sequence, #2] &
       ][[2]]},
    MatrixForm[
     Prepend[
      MapThread[
       {#1,
         Median[#2[[All, 1]] - #2[[All, 2]]],
         Median[Abs[#2[[All, 1]] - #2[[All, 2]]]],
         Median[#3[[All, 1]] - #3[[All, 2]]], Median[
          Abs[#3[[All, 1]] - #3[[All, 2]]]]} &,
       {{"V1", "V2", "V3", "All"},
        RotateLeft[eccerrTmp],
        RotateLeft[polerrTmp]}],
      {"Area", "ϵρ", "|ϵρ|", "ϵθ", "|ϵθ|"}]]]];
```

1.6. Schira Model

Here, the default parameters for the Schira model (ie, those that will be used in fitting via spring simulations)

1.6.1. Default Parameters

```
$DefaultSchiraA = 1.5;
$DefaultSchiraB = 60;
$DefaultSchiraΛ = 2.5;
$DefaultSchiraΨ = -0.05;
$DefaultSchiraV1Size = 0.99;
$DefaultSchiraV2Size = 0.79;
$DefaultSchiraV3Size = 0.38;
$DefaultSchiraHV4Size = 0.3;
$DefaultSchiraFC := {-0.9, 0};
$DefaultSchiraScale := {0.4, -0.25};
$DefaultSchiraShear = {{1, 0}, {0, 1}};
```

```
DeclareKeyword[A, B, Λ, Ψ, V1Size, V2Size,
  V3Size, HV4Size, FC → "FovealConfluence", Shear];
$SchiraParams := List[
   A :→ $DefaultSchiraA,
   B :→ $DefaultSchiraB,
   Λ :→ $DefaultSchiraΛ,
   Ψ :→ $DefaultSchiraΨ,
   V1Size :→ $DefaultSchiraV1Size,
   V2Size :→ $DefaultSchiraV2Size,
   V3Size :→ $DefaultSchiraV3Size,
   HV4Size :→ $DefaultSchiraHV4Size,
   FC :→ $DefaultSchiraFC,
   Scale :→ $DefaultSchiraScale,
   Shear :→ $DefaultSchiraShear];
```

1.6.2. Functions for Calculating and Inverting the Schira model

These functions are highly optimized for speed and are considered ‘low-level’ functions. They implement the forward and backward versions of the Schira model.

```
With[{dsech1 = 0.1821, dsech2 = 0.76},

  Clear[SchiraDoubleSechTransform];
  SchiraDoubleSechTransform = Compile[{{z, _Complex}, {a, _Real}, {λ, _Real}},
    Piecewise[
     {{Abs[z + λ] * Exp[I * Arg[z + λ] *
          (Sech[Arg[z + λ]]^(dsech1 * Sech[dsech2 * Log[Abs[z + λ] / a]]))],
       Re[z] ≥ 0}, {Abs[z + 2 * λ * (1 - Abs[Arg[z]] / Pi)] *
        Exp[I * Arg[z + 2 * λ * (1.0 - Abs[Arg[z]] / Pi)] *
          (Sech[Arg[z + 2 * λ * (1.0 - Abs[Arg[z]] / Pi)]]^
            (dsech1 * Sech[dsech2 * Log[Abs[z + 2 * λ * (1 - Abs[Arg[z]] / Pi)] / a]]))],
       Re[z] < 0}},
     0],
    "RuntimeOptions" → {"Speed", "EvaluateSymbolically" → False}];

  Clear[SchiraLogPolarTransform];
  SchiraLogPolarTransform =
   Compile[{{z, _Complex}, {a, _Real}, {b, _Real}, {λ, _Real}},
    Log[Divide[(SchiraDoubleSechTransform[z, a, λ] + a),
      (SchiraDoubleSechTransform[z, b, λ] + b)]],
```

```
  {{SchiraDoubleSechTransform[_Complex, _Integer, _Real], _Complex}},
  "RuntimeOptions" → {"Speed", "EvaluateSymbolically" → False}];

Clear[SchiraLayerTransform];
SchiraLayerTransform::badarea = "Area must be 1, 2, 3, or 4 (given: `1`)";
SchiraLayerTransform = Compile[
  {{z, _Complex}, {v1b, _Real},
   {v2b, _Real}, {v3b, _Real}, {hv4b, _Real}, {area, _Integer}},
  Abs[z] * Exp[
    Times[I,
     Piecewise[
      {{Arg[z] * 2 / Pi * v1b, area == 1},
       {(Sign[Arg[z]] - Arg[z] * 2 / Pi) * v2b + Sign[Arg[z]] * v1b, area == 2},
       {Arg[z] * 2 / Pi * v3b + Sign[Arg[z]] * (v1b + v2b), area == 3},
       {(Sign[Arg[z]] - Arg[z] * 2 / Pi) * hv4b +
         Sign[Arg[z]] * (v1b + v2b + v3b), area == 4}},
      (Message[SchiraLayerTransform::badarea, area]; 0)]]],
  "RuntimeOptions" → {"Speed", "EvaluateSymbolically" → False}];

Clear[SchiraTransform];
SchiraTransform = Compile[
  {{z, _Complex},
   {a, _Real}, {b, _Real}, {λ, _Real},
   {v1b, _Real}, {v2b, _Real}, {v3b, _Real}, {hv4b, _Real},
   {area, _Integer}},
  SchiraLogPolarTransform[
   SchiraLayerTransform[z, v1b, v2b, v3b, hv4b, area], a, b, λ],
  {{SchiraLogPolarTransform[_Complex, _Real, _Real, _Real], _Complex},
   {SchiraLayerTransform[_Complex,
     _Real, _Real, _Real, _Real, _Integer], _Complex}},
  "RuntimeOptions" → {"Speed", "EvaluateSymbolically" → False}];

Clear[SchiraLayerless];
SchiraLayerless = Compile[
  {{z, _Complex},
   {a, _Real}, {b, _Real}, {λ, _Real}, {ψ, _Real},
   {shearx, _Real}, {sheary, _Real},
   {scalex, _Real}, {scaley, _Real},
   {fcx, _Real}, {fcy, _Real}},
  With[{ww = SchiraLogPolarTransform[z, a, b, λ]},
   With[
    {w =
      (Re[ww] - Log[a / b]) * scalex * (1 + shearx * I) + Im[ww] * scaley * (I + sheary)},
    Re[w] * (Cos[ψ] + I * Sin[ψ]) + Im[w] * (I * Cos[ψ] - Sin[ψ]) + fcx + I * fcy]],
  {{SchiraLogPolarTransform[_Complex, _Real, _Real, _Real], _Complex}},
  "RuntimeOptions" → {"Speed", "EvaluateSymbolically" → False}];

Clear[Schira];
Schira = Compile[
  {{z, _Complex},
   {a, _Real}, {b, _Real}, {λ, _Real},
   {v1b, _Real}, {v2b, _Real}, {v3b, _Real}, {hv4b, _Real}, {area, _Integer},
   {ψ, _Real}, {shearx, _Real}, {sheary, _Real},
```

```
    {scalex, _Real}, {scaley, _Real},
    {fcx, _Real}, {fcy, _Real}},
   With[{ww = SchiraTransform[z, a, b, λ, v1b, v2b, v3b, hv4b, area]},
    With[{w =
       (Re[ww] - Log[a / b]) * scalex * (1 + shearx * I) + Im[ww] * scaley * (I + sheary)},
     Re[w] * (Cos[ψ] + I * Sin[ψ]) + Im[w] * (I * Cos[ψ] - Sin[ψ]) + fcx + I * fcy]],
   {{SchiraTransform[_Complex, _Real,
      _Real, _Real, _Real, _Real, _Real, _Real, _Integer],
     _Complex}},
   "RuntimeOptions" → {"Speed", "EvaluateSymbolically" → False}];

With[
 {compInvFn = Compile[{{zz, _Complex}, {a, _Real}, {b, _Real}, {λ, _Real}},
     (SchiraDoubleSechTransform[zz, a, λ] + a) /
      (SchiraDoubleSechTransform[zz, b, λ] + b),
     {{SchiraDoubleSechTransform[_Complex, _Integer, _Real], _Complex}},
     "RuntimeOptions" → {"Speed", "EvaluateSymbolically" → False}],
  compStartFn = Compile[
     {{z, _Complex},
      {a, _Real}, {b, _Real}, {λ, _Real},
      {v1b, _Real}, {v2b, _Real}, {v3b, _Real}, {hv4b, _Real},
      {ψ, _Real}, {shearx, _Real}, {sheary, _Real},
      {scalex, _Real}, {scaley, _Real},
      {fcx, _Real}, {fcy, _Real}},
     Exp[
      Log[a / b] + Dot[
        {1.0 / scalex, I / scaley},
        Dot[
         Inverse[{{1.0, sheary}, {shearx, 1.0}}],
         RotationMatrix[-ψ],
         {Re[z] - fcx, Im[z] - fcy}]]],
     {{RotationMatrix[_Real], _Real, 2}},
     "RuntimeOptions" → {"Speed", "EvaluateSymbolically" → False}]},

 Clear[InverseSchira];
 InverseSchira[z : (_Real | _Complex), a_Real, b_Real, λ_Real, v1b_Real,
   v2b_Real, v3b_Real, hv4b_Real, ψ_Real, shearx_Real, sheary_Real,
   scalex_Real, scaley_Real, fcx_Real, fcy_Real, undef_: Undefined] := With[
   {ww = compStartFn[z, a, b, λ, v1b, v2b, v3b, hv4b, ψ,
      shearx, sheary, scalex, scaley, fcx, fcy]},
   With[
    {w = FindRoot[
        ww == compInvFn[zz, a, b, λ],
        {zz, 0},
        AccuracyGoal → 5][[1, 2]]},
    Which[
     Abs[Arg[w]] ≤ v1b,
     Abs[w] * Exp[I * Arg[w] / v1b * Pi / 2],
     Abs[Arg[w]] - v1b ≤ v2b,
     Abs[w] *
      Exp[I * (Sign[Arg[w]] * Pi / 2 - (Arg[w] - Sign[Arg[w]] * v1b) / v2b * Pi / 2)],
     Abs[Arg[w]] - v1b - v2b ≤ v3b,
     Abs[w] * Exp[I * (Arg[w] - Sign[Arg[w]] * (v1b + v2b)) / v3b * Pi / 2],
     Arg[w] ≤ 0 && Arg[w] + v1b + v2b + v3b ≥ -hv4b,
```

```
      Abs[w] * Exp[I * (- (Arg[w] + v1b + v2b + v3b + hv4b / 2.0)) / hv4b * Pi],
      True, undef]]];

  Clear[InverseSchiraArea];
  InverseSchiraArea[z_, a_, b_, λ_, v1b_, v2b_, v3b_,
    hv4b_, ψ_, shearx_, sheary_, scalex_, scaley_, fcx_, fcy_] := With[
    {ww = compStartFn[z, a, b, λ, v1b, v2b, v3b, hv4b, ψ,
       shearx, sheary, scalex, scaley, fcx, fcy]},
    With[
     {w = FindRoot[
         ww == compInvFn[zz, a, b, λ],
         {zz, 0},
         AccuracyGoal → 5][[1, 2]]},
     Which[
      Arg[w] < 0 && Abs[Arg[w]] ≤ v1b, -1,
      Arg[w] ≥ 0 && Abs[Arg[w]] ≤ v1b, 1,
      Arg[w] < 0 && Abs[Arg[w]] - v1b ≤ v2b, -2,
      Arg[w] ≥ 0 && Abs[Arg[w]] - v1b ≤ v2b, 2,
      Arg[w] < 0 && Abs[Arg[w]] - v1b - v2b ≤ v3b, -3,
      Arg[w] ≥ 0 && Abs[Arg[w]] - v1b - v2b ≤ v3b, 3,
      Arg[w] < 0 && Abs[Arg[w]] - v1b - v2b - v3b ≤ hv4b, -4,
      Arg[w] ≥ 0 && Abs[Arg[w]] - v1b - v2b - v3b ≤ hv4b, 4,
      True, Undefined]]];

  Clear[InverseSchiraBoth];
  InverseSchiraBoth[z_, a_, b_, λ_, v1b_, v2b_, v3b_,
    hv4b_, ψ_, shearx_, sheary_, scalex_, scaley_, fcx_, fcy_] := With[
    {ww = compStartFn[z, a, b, λ, v1b, v2b, v3b, hv4b, ψ,
       shearx, sheary, scalex, scaley, fcx, fcy]},
    With[
     {w = FindRoot[
         ww == compInvFn[zz, a, b, λ],
         {zz, 0},
         AccuracyGoal → 5][[1, 2]]},
     Which[
      Abs[Arg[w]] ≤ v1b, {
       Abs[w] * Exp[I * Arg[w] / v1b * Pi / 2],
       If[Arg[w] < 0, -1, 1]},
      Abs[Arg[w]] - v1b ≤ v2b, {
       Abs[w] *
        Exp[I * (Sign[Arg[w]] * Pi / 2 - (Arg[w] - Sign[Arg[w]] * v1b) / v2b * Pi / 2)],
       If[Arg[w] < 0, -2, 2]},
      Abs[Arg[w]] - v1b - v2b ≤ v3b, {
       Abs[w] * Exp[I * (Arg[w] - Sign[Arg[w]] * (v1b + v2b)) / v3b * Pi / 2],
       If[Arg[w] < 0, -3, 3]},
      Abs[Arg[w]] - v1b - v2b - v3b ≤ hv4b, {
       Abs[w] * Exp[I * (- (Arg[w] + v1b + v2b + v3b + hv4b / 2.0)) / hv4b * Pi],
       If[Arg[w] < 0, -4, 4]},
      True, Undefined]]];
 ]];

Clear[InvertSchiraData];
InvertSchiraData[dat_List, OptionsPattern[]] := Module[
  {aa = OptionValue[A] /. Automatic → $DefaultSchiraA,
   bb = OptionValue[B] /. Automatic → $DefaultSchiraB,
```

```
      lambda = OptionValue[Λ] /. Automatic → $DefaultSchiraΛ,
      psi = OptionValue[Ψ] /. Automatic → $DefaultSchiraΨ,
      v1b = OptionValue[V1Size] /. Automatic → $DefaultSchiraV1Size,
      v2b = OptionValue[V2Size] /. Automatic → $DefaultSchiraV2Size,
      v3b = OptionValue[V3Size] /. Automatic → $DefaultSchiraV3Size,
      hv4b = OptionValue[HV4Size] /. Automatic → $DefaultSchiraHV4Size,
      fc = OptionValue[FC] /. Automatic → $DefaultSchiraFC,
      scale = OptionValue[Scale] /. Automatic → $DefaultSchiraScale,
      shear = OptionValue[Shear] /. Automatic → $DefaultSchiraShear},
     Quiet[
      Map[
       InverseSchiraBoth[
         #1[[1]] + I * #1[[2]],
         aa, bb, lambda, v1b, v2b, v3b, hv4b, psi,
         shear[[1, 2]], shear[[2, 1]], scale[[1]], scale[[2]], fc[[1]], fc[[2]]] &,
       dat]]];
Attributes[InvertSchiraData] = {HoldRest};
Options[InvertSchiraData] = List[
     A → Automatic, B → Automatic, Λ → Automatic, Ψ → Automatic,
     V1Size → Automatic, V2Size → Automatic, V3Size → Automatic, HV4Size → Automatic,
     FC → Automatic, Scale → Automatic, Shear → Automatic];

Clear[SchiraModel];
SchiraModel[z_, area_, OptionsPattern[]] := Module[
     {aa = OptionValue[A] /. Automatic → $DefaultSchiraA,
      bb = OptionValue[B] /. Automatic → $DefaultSchiraB,
      lambda = OptionValue[Λ] /. Automatic → $DefaultSchiraΛ,
      psi = OptionValue[Ψ] /. Automatic → $DefaultSchiraΨ,
      v1b = OptionValue[V1Size] /. Automatic → $DefaultSchiraV1Size,
      v2b = OptionValue[V2Size] /. Automatic → $DefaultSchiraV2Size,
      v3b = OptionValue[V3Size] /. Automatic → $DefaultSchiraV3Size,
      hv4b = OptionValue[HV4Size] /. Automatic → $DefaultSchiraHV4Size,
      fc = OptionValue[FC] /. Automatic → $DefaultSchiraFC,
      scale = OptionValue[Scale] /. Automatic → $DefaultSchiraScale,
      shear = OptionValue[Shear] /. Automatic → $DefaultSchiraShear},
     Schira[
      z,
      aa, bb, lambda, v1b, v2b, v3b, hv4b, area, psi,
      shear[[1, 2]], shear[[2, 1]], scale[[1]], scale[[2]], fc[[1]], fc[[2]]]];
Options[SchiraModel] = List[
     A → Automatic, B → Automatic, Λ → Automatic, Ψ → Automatic,
     V1Size → Automatic, V2Size → Automatic, V3Size → Automatic, HV4Size → Automatic,
     FC → Automatic, Scale → Automatic, Shear → Automatic];

Clear[ExportSchiraParameters];
ExportSchiraParameters[filename_String, OptionsPattern[]] := With[
     {aa = OptionValue[A] /. Automatic → $DefaultSchiraA,
      bb = OptionValue[B] /. Automatic → $DefaultSchiraB,
      lambda = OptionValue[Λ] /. Automatic → $DefaultSchiraΛ,
      psi = OptionValue[Ψ] /. Automatic → $DefaultSchiraΨ,
      v1b = OptionValue[V1Size] /. Automatic → $DefaultSchiraV1Size,
      v2b = OptionValue[V2Size] /. Automatic → $DefaultSchiraV2Size,
      v3b = OptionValue[V3Size] /. Automatic → $DefaultSchiraV3Size,
      hv4b = OptionValue[HV4Size] /. Automatic → $DefaultSchiraHV4Size,
      fc = OptionValue[FC] /. Automatic → $DefaultSchiraFC,
```

```
    scale = OptionValue[Scale] /. Automatic → $DefaultSchiraScale,
    shear = OptionValue[Shear] /. Automatic → $DefaultSchiraShear},
   Export[
    filename,
    Apply[
     StringJoin,
     Map[
      {#[[1]], "\t", ToString[#[[2]]], "\n"} &,
      {{"a", aa},
       {"b", bb},
       {"psi", psi},
       {"FCx", fc[[1]]},
       {"FCy", fc[[2]]},
       {"Scalex", scale[[1]]},
       {"Scaley", scale[[2]]},
       {"Shearx", shear[[1, 2]]},
       {"Sheary", shear[[2, 1]]},
       {"V1Size", v1b},
       {"V2Size", v2b},
       {"V3Size", v3b},
       {"HV4Size", hv4b},
       {"lambda", lambda}}]],
    "Text"]];
ExportSchiraParameters[filename_String, mdl_SchiraModelObject] := Apply[
   ExportSchiraParameters,
   Prepend[List @@ mdl, filename]];
Options[ExportSchiraParameters] = List[
   A → Automatic, B → Automatic, Λ → Automatic, Ψ → Automatic,
   V1Size → Automatic, V2Size → Automatic, V3Size → Automatic, HV4Size → Automatic,
   FC → Automatic, Scale → Automatic, Shear → Automatic];
```

```
Clear[UsingSchiraParams];
UsingSchiraParams[filename_String, block_] := Module[
   {A, B, lambda, psi, v1b, v2b, v3b, hv4b,
    fcx, fcy, scalex, scaley, shearx, sheary},
   ReplaceAll[
    Partition[
     Import[filename, "Table"],
     2],
    {{"a", a_} :> (A = a),
     {"b", b_} :> (B = b),
     {"psi", ψ_} :> (psi = ψ),
     {"FCx", x_} :> (fcx = x),
     {"FCy", y_} :> (fcy = y),
     {"Scalex", x_} :> (scalex = x),
     {"Scaley", y_} :> (scaley = y),
     {"Shearx", x_} :> (shearx = x),
     {"Sheary", y_} :> (sheary = y),
     {"V1Size", v_} :> (v1b = v),
     {"V2Size", v_} :> (v2b = v),
     {"V3Size", v_} :> (v3b = v),
     {"HV4Size", v_} :> (hv4b = v),
     {"lambda", l_} :> (lambda = l)}];
   Block[
    {$DefaultSchiraA = A,
     $DefaultSchiraB = B,
     $DefaultSchiraΛ = lambda,
     $DefaultSchiraΨ = psi,
     $DefaultSchiraV1Size = v1b,
     $DefaultSchiraV2Size = v2b,
     $DefaultSchiraV3Size = v3b,
     $DefaultSchiraHV4Size = hv4b,
     $DefaultSchiraFC = {fcx, fcy},
     $DefaultSchiraScale = {scalex, scaley},
     $DefaultSchiraShear = {{1, shearx}, {sheary, 1}}},
    block]];
Attributes[UsingSchiraParams] = {HoldRest};
```

1.6.3. Schira Model Object

Schira Models are basically parameter sets that know how to plot/draw/calculate values

```
Unprotect[SchiraModelObject];
Clear[SchiraModel];
SchiraModel::badarg = "Bad argument(s); all arguments must be rules: `1`";
SchiraModel[
   opts : Evaluate[
     Repeated[
      (Rule | RuleDelayed)[
       Apply[Alternatives, First /@ $SchiraParams],
       _]]]
  ] := Apply[
   SchiraModelObject,
   ReplaceAll[
    $SchiraParams,
    Map[
     Function[{rule},
      Rule[
       (First[rule] :> _),
       Head[rule][First[rule], Last[rule]]]],
     {opts}]]];
SchiraModel[] := Apply[SchiraModelObject, $SchiraParams];
SchiraModel[
   mdl_SchiraModelObject,
   opts : Evaluate[
     Repeated[
      (Rule | RuleDelayed)[
       Apply[Alternatives, First /@ $SchiraParams],
       _]]]
  ] := Apply[
   SchiraModelObject,
   ReplaceAll[
    List @@ mdl,
    Map[
     Function[{rule},
      Rule[
       (First[rule] :> _),
       Head[rule][First[rule], Last[rule]]]],
     {opts}]]];
Module[{schiraPositions},
  MapThread[
   Function[{sym, idx},
    schiraPositions[sym] = idx],
   {$SchiraParams[[All, 1]],
    Range[Length@$SchiraParams]}];
  (* ask a model for parameter values *)
  SchiraModelObject[opts__][
    x : Evaluate[Apply[Alternatives, $SchiraParams[[All, 1]]]]] :=
   Replace[x, {opts}[[schiraPositions[x]]]]];
```

SchiraFunction[] asks a schira model for its function; it returns a fun that takes th (in visual field degrees), r, and the area (or a list of areas: {1,2,3,4}) in which to calculate the Schira model value.

```
(* Ask a model for its equation *)
Unprotect[SchiraFunction];
Clear[SchiraFunction];
SchiraFunction::badarg = "Area argument must be 1, 2, 3, or 4";
SchiraFunction::insufarg = "Schira function requres θ, r, and an area argument";
SchiraFunction[mdl_SchiraModelObject] := With[
   {a = mdl[A],
    b = mdl[B],
    lambda = mdl[Λ],
    v1b = mdl[V1Size],
    v2b = mdl[V2Size],
    v3b = mdl[V3Size],
    v4b = mdl[HV4Size],
    psi = mdl[Ψ],
    shearx = mdl[Shear][[1, 2]],
    sheary = mdl[Shear][[2, 1]],
    scalex = mdl[Scale][[1]],
    scaley = mdl[Scale][[2]],
    fcx = mdl[FC][[1]],
    fcy = mdl[FC][[2]]},
   Compile[{{θ, _Real}, {r, _Real}, {area, _Integer}},
    If[0 < area ≤ 5,
     Schira[
      r * Exp[(Pi / 2 - θ / 180 * Pi) * I],
      a, b, lambda, v1b, v2b, v3b, v4b, area, psi,
      shearx, sheary, scalex, scaley, fcx, fcy],
     (Message[SchiraFunction::badarg]; $Failed)],
    {{Schira[__], _Complex}},
    "RuntimeOptions" → {"Speed", "EvaluateSymbolically" → False}]];
Protect[SchiraFunction];
```

```
(* Ask a model for its equation *)
Unprotect[InverseSchiraFunction];
Clear[InverseSchiraFunction];
InverseSchiraFunction::badarg =
  "Point (`1`,`2`) is not predicted by Schira function";
InverseSchiraFunction[mdl_SchiraModelObject] := With[
   {a = mdl[A],
    b = mdl[B],
    lambda = mdl[Λ],
    v1b = mdl[V1Size],
    v2b = mdl[V2Size],
    v3b = mdl[V3Size],
    hv4b = mdl[HV4Size],
    psi = mdl[Ψ],
    shearx = mdl[Shear][[1, 2]],
    sheary = mdl[Shear][[2, 1]],
    scalex = mdl[Scale][[1]],
    scaley = mdl[Scale][[2]],
    fcx = mdl[FC][[1]],
    fcy = mdl[FC][[2]]},
   With[
    {lab = Log[a / b],
     srot = (Inverse[{{1.0, sheary}, {shearx, 1.0}}].RotationMatrix[-psi]) *
       {1.0 / scalex, I / scaley}},
```

```
(* we make a function that analytically inverts what it can... *)
With[
 {startFn = Compile[{{xx, _Real}, {yy, _Real}},
    Exp[lab + Total[srot.{xx - fcx, yy - fcy}]],
    "RuntimeOptions" → {"Speed", "EvaluateSymbolically" → False}],
  searchFn = Compile[{{zz, _Complex}},
    Divide[
     (SchiraDoubleSechTransform[zz, a, lambda] + a),
     (SchiraDoubleSechTransform[zz, b, lambda] + b)],
    {{SchiraDoubleSechTransform[_Complex, _Real, _Real], _Complex}},
    "RuntimeOptions" → {"Speed", "EvaluateSymbolically" → False}]},
 Function[{x, y},
  With[{ww = startFn[x, y]},
   With[
    {w = FindRoot[
        ww == searchFn[zz],
        {zz, 0},
        AccuracyGoal → 5
       ][[1, 2]]},
    With[
     {absargw = Abs[Arg[w]]},
     Which[
      absargw ≤ v1b, {90.0 - 180.0 / Pi * Arg[w] / v1b * Pi / 2, Abs[w], 1},
      absargw - v1b ≤ v2b, {
       90.0 - 180.0 / Pi *
         (Sign[Arg[w]] * Pi / 2 - (Arg[w] - Sign[Arg[w]] * v1b) / v2b * Pi / 2),
       Abs[w], 2},
      absargw - v1b - v2b ≤ v3b, {
       90.0 - 180.0 / Pi * (Arg[w] - Sign[Arg[w]] * (v1b + v2b)) / v3b * Pi / 2,
       Abs[w], 3},
      Arg[w] ≤ 0 && Arg[w] + v1b + v2b + v3b ≥ -hv4b, {
       90.0 - 180.0 / Pi * (- (Arg[w] + v1b + v2b + v3b + hv4b / 2.0)) / hv4b * Pi,
       Abs[w], 4},
      True, Undefined]]]]]]];
Protect[InverseSchiraFunction];
```

1.6.4. Schira Model Plotting

```
SchiraDensityPlot[sobj_, type : PolarAngle | Eccentricity,
   opts : OptionsPattern[ListDensityPlot]] := Module[
   {fun = SchiraFunction[sobj],
    conv = If[type === PolarAngle, {Re@#1, Im@#1, #2} &, {Re@#1, Im@#1, #3} &]},
   ListDensityPlot[
    Flatten[
     Table[
      conv[fun[θ, r, a], θ, r],
      {θ, 0, 180, 2},
      {r, 0, 20, 1},
      {a, 1, 4}],
     2],
    opts]];
```

```
DeclareKeyword[
  EccentricityStyleFunction,
  PolarAngleStyleFunction,
```

```
    EccentricityLines,
    PolarAngleLines];
Clear[SchiraLinePlot];
SchiraLinePlot[sobj_, opts : OptionsPattern[]] := Block[
   {θ, r, u},
   Module[
    {fun = SchiraFunction[sobj], a,
     θMax = 89.9999999,
     rMax = 90.0,
     ps,
     esf = Replace[
       OptionValue[EccentricityStyleFunction],
       x : (Automatic | Thick | Thin | Dotted | Dashed) :→ (If[
             x === Automatic, #, {x, #}] &@Which[
            # > 90, Black,
            # < 0, LightGray,
            True, Blend[{LightGray, Black}, #1 / 90.0]] &)],
     psf = Replace[
       OptionValue[PolarAngleStyleFunction],
       x : (Automatic | Thick | Thin | Dotted | Dashed) :→ (If[
             x === Automatic, #, {x, #}] &@Which[
            # < 0, Blue,
            # > 180, Red,
            True, Hue[2. / 3. * (1 - #1 / 180.0)]] &)],
     eclines = Replace[
       OptionValue[EccentricityLines],
       {None → {},
        x_?NumberQ :→ {x},
        Automatic → {1.25, 2.5, 5.0, 10.0, 20.0, 40.0, 90.0}}],
     palines = ReplaceAll[
       Replace[
        OptionValue[PolarAngleLines],
        {x_?NumberQ :→ {x},
         Automatic → {0.0, 45.0, 90.0, 135.0, 180.0}}],
       None → {}]},
    {{a}, {ps}} = Quiet[
      Last[
       Reap[
        Do[
         (Sow[{Re@#, Im@#} &[fun[If[q, θMax * u, 180 - θMax * u], r, area]], Data];
          Sow[esf[r, a], PlotStyle]),
         {r, Evaluate[eclines]},
         {area, 1, 3, 1},
         {q, {True, False}}];
        If[
         Length@palines == 3 && And @@ (ListQ /@ palines),
         Do[
          Which[
           (a == 2) && (θ == 90 || θ == 90.0), (
            Sow[{Re@#, Im@#} &[fun[θMax, u * rMax, a]], Data];
            Sow[{Re@#, Im@#} &[fun[180.0 - θMax, u * rMax, a]], Data];
            Sow[psf[θMax, a], PlotStyle];
            Sow[psf[180.0 - θMax, a], PlotStyle]),
           (a ≠ 3 || (θ ≠ 90 && θ ≠ 90.0)), (
            Sow[{Re@#, Im@#} &[fun[θ, u * rMax, a]], Data];
```

```
            Sow[psf[θ, a], PlotStyle])],
          {a, 1, 3, 1},
          {θ, Evaluate[palines[[a]]]}],
         Do[
          Which[
           (a == 2) && (θ == 90 || θ == 90.0), (
            Sow[{Re@#, Im@#} &[fun[θMax, u * rMax, a]], Data];
            Sow[{Re@#, Im@#} &[fun[180.0 - θMax, u * rMax, a]], Data];
            Sow[psf[θMax, a], PlotStyle];
            Sow[psf[180.0 - θMax, a], PlotStyle]),
           (a ≠ 3 || (θ ≠ 90 && θ ≠ 90.0)), (
            Sow[{Re@#, Im@#} &[fun[θ, u * rMax, a]], Data];
            Sow[psf[θ, a], PlotStyle])],
          {θ, Evaluate[palines]},
          {a, 1, 3, 1}]],
        {Data, PlotStyle}]]];
    ParametricPlot[
     a,
     {u, 0, 1},
     PlotStyle → ps,
     Evaluate[FilterRules[{opts}, Options[ParametricPlot]]]]]]];
Options[SchiraLinePlot] = Join[
   FilterRules[Options[ParametricPlot], Except[PlotStyle]],
   {EccentricityStyleFunction → Automatic,
    PolarAngleStyleFunction → Automatic,
    EccentricityLines → Automatic,
    PolarAngleLines → Automatic}];
```

1.6.5. Choosing a Schira Model for Use in Registration

Here we have code for examining the Schira Model via a manipulate function (see *Mathematica*'s documentation on the Manipulate function). This defines the function, but does not execute it; if you would like to explore the Schira model space, you just have to evaluate SchiraModelExplore[] or change the cell below containing that expression from a text to an input cell and evaluate it; we have left it as a text cell to avoid side-effects when evaluating the entire introduction section.
It is, by the way, best to run this exploration function after setting the data10 thresholds (below, in section 3.2). Additionally, be forewarned that manipulating the Schira function in this fashion is a slow and difficult rendering for *Mathematica*, and will not be smooth unless you are operating on a very powerful computer.

```
SchiraModelExplore[] := With[
   {paFig = CorticalPlot[
      SelectPolarAngle[data10[Aggregate], 0],
      ColorFunction → $PolarAngleColorFn,
      ImageSize → 3 * 72],
    ecFig = CorticalPlot[
      SelectEccentricity[data10[Aggregate], 0],
      ColorFunction → $EccentricityColorFn,
      ImageSize → 3 * 72]},
   Manipulate[
    With[
```

```
{mdl = SchiraModel[
   A → a,
   B → b,
   Λ → λ,
   Ψ → ψ,
   Scale → {scaleX, -scaleY},
   FC → {FCx, FCy}]},
 Graphics[
  {Inset[
    Show[
     {paFig,
      SchiraLinePlot[
       mdl,
       PolarAngleStyleFunction →
        ({Thickness[0.006], Darker[$PolarAngleColorFn[#]]} &),
       EccentricityStyleFunction → ({Thickness[0.004], Dashed, White} &),
       EccentricityLines → {10, 90},
       Axes → None,
       Frame → False]}],
    ImageScaled[{0, 0.5}],
    ImageScaled[{0, 0.5}]],
   Inset[
    Show[
     {ecFig,
      SchiraLinePlot[
       mdl,
       EccentricityStyleFunction →
        ({Thickness[0.006], Darker[$EccentricityColorFn[#]]} &),
       PolarAngleStyleFunction → ({Thickness[0.004], White} &),
       PolarAngleLines → {{0, 180}, None, {0, 180}},
       Axes → None,
       Frame → False]}],
    ImageScaled[{1, 0.5}],
    ImageScaled[{1, 0.5}]]},
  ImageSize → {6.5, 3.5} * 72,
  ImagePadding → 2000]],
{{a, 0.7}, 0.001, 10},
{{b, 90.0}, 1, 120},
{{λ, 0.1}, 0, Pi},
{{ψ, 0}, -Pi, Pi},
{{scaleX, 0.225}, 0.1, 6.0},
{{scaleY, 0.225}, 0.1, 6.0},
{{FCx, -0.6}, -1, 1},
{{FCy, 0}, -1, 1}]];
```

Here we define the model that we use in this notebook and for simulations. It is also the Schira model that uses all of the default parameters.

SchiraModelExplore[]

```
OurSchiraModel := (OurSchiraModel = SchiraModel[]);
```

1.7. Loading and Filtering Subject Data

This function load’s a single subject’s VTK files; it assumes a particular directory and file structure found in our data directories. The option RHInverted tells the function that the

right hemisphere data should be inverted (it should not be for fsaverage-sym hemispheres). The option FStatSeparated tells the function that the FStat values for the subject are stored in two files (one for polar angle and one for eccentricity) instead of just one file for both coordinates.

```
LoadSubjectVTK[sub_String, hemi : (LH | RH), OptionsPattern[]] := With[
   {ecc = ReadVTK[
      $SubjectDirectory <> "/Eccen/" <> sub <> "-" <> ToString[hemi] <> ".vtk"],
    pol = ReadVTK[
      $SubjectDirectory <> "/Angle/" <> sub <> "-" <> ToString[hemi] <> ".vtk"],
    curv = With[
      {curvfl =
        $SubjectDirectory <> "/Curv/" <> sub <> "-" <> ToString[hemi] <> ".vtk"},
      Replace[
       OptionValue[Curvature],
       {Except[Automatic | True | False] :>
         (Die["Curvature option must be Automatic, True, or False"]),
        False → {},
        True :> Check[
          ReadVTK[curvfl],
          Die["Could not find curvature file"]],
        Automatic :> Check[ReadVTK[curvfl], {}]}]],
    precision = 10.0^(-OptionValue[Precision]),
    data = Unique["data"]},
   Which[
    OptionValue[FStatSeparated] === False, With[
     {fst = ReadVTK[
        $SubjectDirectory <> "/Fstat/" <> sub <> "-" <> ToString[hemi] <> ".vtk"]},
     With[
      {ec = Round[ecc[[All, 1]], precision],
       pa = Round[pol[[All, 1]], precision],
       fs = Round[fst[[All, 1]], precision],
       cu = Round[If[curv == {}, ecc, curv][[All, 1]], precision]},
      If[! (ec == pa == fs == cu),
       Die["Error: Subject " <> sub <> " does not have congruent data"]];
      Select[
       V1Transform[
        MapThread[
         If[hemi === RH,
          Join[#1[[1]], -#1[[2]], #2[[2]], #3[[2]], #4[[2]]] &,
          Join[#1[[1]], #1[[2]], #2[[2]], #3[[2]], #4[[2]]] &],
         {pol, ecc, fst,
          If[Length@curv > 0, curv, Table[{0, {}}, {Length@pol}]]}]],
       OptionValue[Select]]]],
    OptionValue[FStatSeparated] === True, With[
     {fst = List[
        ReadVTK[
         $SubjectDirectory <>
          "/Fstat/" <> sub <> "-angle-" <> ToString[hemi] <> ".vtk"],
        ReadVTK[
         $SubjectDirectory <>
          "/Fstat/" <> sub <> "-eccen-" <> ToString[hemi] <> ".vtk"]]},
     With[
      {ec = Round[ecc[[All, 1]], precision],
```

```
          pa = Round[pol[[All, 1]], precision],
          f1 = Round[fst[[1, All, 1]], precision],
          f2 = Round[fst[[2, All, 1]], precision],
          cu = Round[If[curv == {}, ecc, curv][[All, 1]], precision]},
         If[! (ec == pa == f1 == f2 == cu),
          Die["Error: Subject " <> sub <> " does not have congruent data"]];
         Select[
          V1Transform[
           MapThread[
            If[hemi === RH && OptionValue[RHInverted],
             Join[#1[[1]], -#1[[2]],
               #2[[2]], {Min[{#3[[2, 1]], #4[[2, 1]]}]}, #5[[2]]] &,
             Join[#1[[1]], #1[[2]], #2[[2]], {Min[{#3[[2, 1]], #4[[2, 1]]}]},
               #5[[2]]] &],
            {pol, ecc, fst[[1]], fst[[2]], If[Length@curv > 0, curv,
              Table[{0, {}}, {Length@pol}]]}]],
          OptionValue[Select]]]],
     True, Die["FStatSeparated must be True or False"]]];
Options[LoadSubjectVTK] = {
   Select → (#[[5]] > 0 &),
   RHInverted → True,
   FStatSeparated → False,
   Curvature → Automatic,
   Precision → 5};
DeclareKeyword[FStatSeparated, RHInverted, Curvature];
```

Used for filtering subjects; with a subject, we define an extra value, q, that may be used in the filter; q is the quantile of the point’s f-stat.

```
Clear[FilterSubject];
DeclareKeyword[SubjectFilter];
Options[FilterSubject] = {
   SubjectFilter → Automatic,
   Hemisphere → All};
Attributes[FilterSubject] = {HoldRest};
FilterSubject[dataset_, subject_, OptionsPattern[]] := With[
   {hemi = OptionValue[Hemisphere],
    filt = Block[{x, y, θ, r, f, q},
      Replace[
       OptionValue[SubjectFilter],
       Automatic → dataset[SubjectFilter]]]},
   With[
    {sub =
      If[hemi === All, dataset[Data, subject], {dataset[Data, hemi, subject]}]},
    With[
     {dists = Map[
        hem Ì EmpiricalDistribution[Select[hem[[All, 5]], # > 0 &]],
        sub]},
     With[
      {res = MapThread[
         {hem, dist} Ì Flatten[
           Last[
            Reap[
             Scan[
              row Ì Block[
                {x = row[[1]], y = row[[2]],
                 θ = row[[3]], r = row[[4]],
                 f = row[[5]], q = CDF[dist, row[[5]]]},
                If[filt, Sow[row]]],
              hem]]],
           1],
         {sub, dists}]},
      If[Length@res == 1, First@res, res]]]]];
```

1.8. Delaunay Mesh Registration

The VoronoiRegister function performs an interative registration of the given data to the given function by first calculating (or being given) a Delaunay Triangulation, from which the Voronoi edges are inferred. Without changing this triangulation, vertices are, each step, moved halfway from their current position toward the intersection of the line from their position to their ideal position with the edge of the Delaunay tesselation polygon containing them (whose vertices never change despite changes in the geometry of the points).
This function is made to be used with SchiraFunction and the aggregate of a dataset.

Note that you can't actually pass a SchiraFunction in to this function because it's made to be more versatile than to only work with Schira models; accordingly, there is a Schira-ToVoronoiFunction for conversion.

```
Clear[VoronoiRegister];
DeclareKeyword[Epsilon, MaximumDistance, Steps];
Options[VoronoiRegister] = {
```

```
    DelaunayTriangulation → Automatic,
    FStat → {20.0, 30.0},
    Epsilon → 0.00001,
    MaximumDistance → 0.2,
    Steps → 25,
    While → Steps};
VoronoiRegister[data_, fn_, OptionsPattern[]] := With[
    {XY = data[[All, 1 ;; 2]],
     ZS = data[[All, 3 ;; All]],
     hullIdcs = ConvexHull[data[[All, 1 ;; 2]]],
     fRng = With[
       {f = OptionValue[FStat]},
       Which[
        MatchQ[f, {mn_?NumberQ, mx_?NumberQ} /; 0 ≤ mn < mx], f,
        f === None, {0, ∞},
        True, Die["FStat must be of the form {min,max} or None"]]],
     ϵ = N@Replace[
        OptionValue[Epsilon],
        {x_ /; (! NumberQ[x] || x < 0) :→ Die["Invalid Epsilon Value"],
         0 → $MachineEpsilon}],
     dMin = Replace[
       OptionValue[MaximumDistance],
       x_ /; (! NumberQ[x] || x ≤ 0) :→ Die["Invalid MaximumDistance Value"]],
     steps = OptionValue[Steps]},
    With[
     {restIdcs = Complement[Range[Length@data], hullIdcs],
      inf = Max[Abs[Flatten[XY]]] - Min[Abs[Flatten[XY]]] + 1.0,
      endCond = Replace[
        OptionValue[While],
        Steps → Function[{step, fx, ffx},
          And[
           step < steps,
           Length[Select[Norm /@ (fx - ffx), # > 0.00001 &]] > 0]]]},
     With[
      {order = Join[hullIdcs, restIdcs]},
      With[
       {rorder = Ordering[order],
        xy = XY[[order]],
        zs = ZS[[order]],
        dat = data[[order]]},
       With[
        {neighbors = Replace[
           OptionValue[DelaunayTriangulation],
           {(*Except[Automatic|_?(DelaunayTriangulationQ[xy,#]&)]:→
             Die["Invalud Delaunay Triangulation"],*)
            Automatic :→ DelaunayTriangulation[xy][[All, 2]],
            (dt : {{_Integer, _List} ..}) :→ Map[
              rorder[[#]] &,
              dt[[order, 2]]],
            (dt : {_List ..}) :> Map[
              rorder[[#]] &,
              dt[[order]]],
            _ :→ Die["Bad form for DelaunayTriangulation option"]}]},
        With[
```

```
{sincs = Map[
   row ↦ If[row[[5]] > fRng[[1]],
     With[
      {s = fn[row[[3 ;; -1]]],
       pt = row[[1 ;; 2]]},
      If[Min[Norm[# - pt] & /@ s] > dMin, {{}}, s]],
     {{}}],
   dat]},
With[
 {updateSingleVertex = (*Compile[
    {{neighbors,_Real,2},{sincs,_Real,2}},*)
   Function[{neighbors, sincs},
    With[
     {x = Mean[neighbors]},
     (* Find closest sinc *)
     With[
      {sinc = sincs[[First@Ordering[Norm[# - x] & /@ sincs, 1]]]},
      With[
       {u = Normalize[sinc - x],
        d = Norm[sinc - x]},
       If[d < ϵ,
        x,
        With[
         {dwall = Min[
             MapThread[
              With[
                {v = Normalize[#2 - #1],
                 y = #1},
                If[
                 Or[
                  Round[v, ϵ] == Round[u, ϵ],
                  Round[-v, ϵ] == Round[u, ϵ]],
                 inf,
                 With[
                  {dd = Plus[
                       y[[1]] - x[[1]],
                       Times[
                        v[[1]],
                        Divide[
                         (u[[2]] * x[[1]] + u[[1]] * y[[2]] -
                            u[[1]] * x[[2]] - u[[2]] * y[[1]]),
                         (u[[2]] * v[[1]] - v[[2]] * u[[1]])]]
                      ] / u[[1]]},
                  If[dd < 0, inf, dd]]]
               ] &,
              {Most@neighbors, Rest@neighbors}]]},
         If[dwall ≥ inf || dwall < 0,
          x,
          x + 0.5 * (u * If[d < dwall, d, dwall])]]]]]]]},
 With[
  {which = Table[
     If[Length[Flatten[sincs[[k]]]] > 0, k, -k],
     {k, 1, Length@sincs}]},
  With[
   {singleStep = Function[{X},
```

```
              Join[
               X[[1 ;; Length@hullIdcs]],
               Map[
                Function[{k},
                 If[k > 0,
                  updateSingleVertex[
                   X[[#]] & /@ neighbors[[k]],
                   sincs[[k]]],
                  Mean[X[[neighbors[[-k]]]]]]]],
                which[[(Length[hullIdcs] + 1) ;; All]]]]]},
           (* now run steps... *)
           NestWhile[
             {#[[1]] + 1, singleStep[#[[2]]], #[[2]]} &,
             {0, xy, xy + 1.0},
             endCond[#[[1]], #[[2]], #[[3]]] &
            ][[2, rorder]]]]]]]]]]];
```

This variable can be used later to assert that the topology of whatever we give the VoronoiRegister function should actually match the topology of the LocalAnatomy

```
SchiraToVoronoiFunction[fn_] := Compile[{{dat, _Real, 1}},
   Table[
    With[{z = fn[dat[[1]], dat[[2]], k]}, {Re@z, Im@z}],
    {k, 1, 4}],
   {{fn[_, _, _], _Complex}}];
```

```
LocalDelaunayTriangulation := Set[
   LocalDelaunayTriangulation,
   DelaunayTriangulation[LocalAnatomy[[All, 1 ;; 2]]]];
```

1.9. Combining/Outputting/Manipulating Subject Data

1.9.1. Aggregate two or more maps across vertices

```
MedianJointMap[subs_List, filt_: True] :=
  Map[
   Function[{pt},
    Module[
     {dat = Select[
        pt,
        Block[{x = #[[1]], y = #[[2]], θ = #[[3]], r = #[[4]], f = #[[5]]}, filt] &]},
     If[Length@dat == 0,
      {pt[[1, 1]], pt[[1, 2]], 0, 0, 0},
      {pt[[1, 1]], pt[[1, 2]],
       Median[dat[[All, 3]]],
       Median[dat[[All, 4]]],
       Median[dat[[All, 5]]]}]]],
   Transpose[subs]];
Attributes[MedianJointMap] = {HoldRest};
```

```
Clear[MeanJointMap];
Attributes[MeanJointMap] = {HoldRest};
MeanJointMap[subs_List, filt_: True, weight_: 1] := With[
   {fixedSubjects = With[
      {allpts = Union[Flatten[subs[[All, All, 1 ;; 2]], 1]]},
      Map[
       sub Ì Map[
         pt Ì Which[
            Length@pt == 1, Join[pt[[1]], {0, 0, 0}],
            Length@pt[[1]] > Length@pt[[2]], pt[[1]],
            True, pt[[2]]],
         SplitBy[SortBy[Join[sub, allpts], #[[1 ;; 2]] &], #[[1 ;; 2]] &]],
       subs]]},
   Map[
    Function[{subsAtVertex},
     With[
      {dat = Select[
         subsAtVertex,
         Block[{x = #[[1]], y = #[[2]], θ = #[[3]], r = #[[4]], f = #[[5]]}, filt] &]},
      If[Length@dat == 0,
       {subsAtVertex[[1, 1]], subsAtVertex[[1, 2]], 0, 0, 0},
       With[
        {w = Map[
           Block[
             {x = #[[1]], y = #[[2]], θ = #[[3]], r = #[[4]], f = #[[5]]}, weight] &,
           dat]},
        Join[
         subsAtVertex[[1, 1 ;; 2]],
         If[Total[w] > 0,
          {Dot[dat[[All, 3]], w] / Total[w],
           Dot[dat[[All, 4]], w] / Total[w],
           Dot[dat[[All, 5]], w] / Total[w]},
          {0, 0, 0}]]]]]],
    Transpose[fixedSubjects]]];

Clear[LocalAnatomyFill];
LocalAnatomyFill[agg_] := With[
   {zeros = Table[0, {Dimensions[agg][[2]]}]},
   Map[
    pt Ì Which[
      Length[pt] == 1 && Length[pt[[1]]] == 2, Join[pt[[1]], zeros],
      Length[pt] == 1, pt[[1]],
      Length[pt[[1]]] == 2, pt[[2]],
      True, pt[[1]]],
    SplitBy[
     SortBy[Join[agg, LocalAnatomy[[All, 1 ;; 2]]], #[[1 ;; 2]] &],
     #[[1 ;; 2]] &]]];
```

1.9.2. A distribution matcher

```
MatchDistribution[distRule_Rule] := With[
   {a = EmpiricalDistribution[distRule[[1]]],
    b = EmpiricalDistribution[distRule[[2]]]},
   Map[
    InverseCDF[b, CDF[a, #]] &,
    distRule[[1]]]];
```

```
MatchPolarAngles[Rule[a_List, b_List],
   fMn_: 20, {eccMn_: 1.25, eccMx_: 8.75}] := With[
   {A = EmpiricalDistribution[
      Select[a, #[[5]] ≥ fMn && eccMn ≤ #[[4]] ≤ eccMx && 0 < #[[3]] < 180 &][[All, 3]]],
    B = EmpiricalDistribution[
      Select[b, #[[5]] ≥ fMn && eccMn ≤ #[[4]] ≤ eccMx && 0 < #[[3]] < 180 &][[All, 3]]]},
   With[
    {f = Function[{t}, InverseCDF[B, CDF[A, t]]]},
    Map[
     If[#[[5]] > fMn && eccMn ≤ #[[4]] ≤ eccMx && 0 < #[[3]] < 180,
       {#[[1]], #[[2]], f[#[[3]]], #[[4]], #[[5]]},
       {#[[1]], #[[2]], 0, 0, 0}
      ] &,
     a]]];
```

1.9.3. A function for matching two datasets to each other by index

```
MatchOrder[Rule[A_List, B_List] /; Length[A] == Length[B], OptionsPattern[]] := With[
   {tol = 10^-Replace[
        OptionValue[Precision],
        {Automatic → 5,
         e_Integer /; e > 0 :→ e,
         _ :→ Die["Precision must be a positive integer"]}]},
   With[
    {sortFn = Function[
       Which[
        #1[[1]] - #2[[1]] < -tol, True,
        #1[[1]] - #2[[1]] > tol, False,
        #1[[2]] - #2[[2]] < -tol, True,
        #1[[2]] - #2[[2]] > tol, False,
        True, True]]},
    Part[
     Sort[A, sortFn],
     Flatten[
      Reap[
        MapThread[Sow, {Range[Length@B], Ordering[B, Length@B, sortFn]}],
        Range[Length@B]
       ][[2]]]]]];
Options[MatchOrder] = {Precision → Automatic};
```

1.9.4. Low-level functions for writing and reading simulation binary files

```
Clear[WriteSimStartFile];
WriteSimStartFile[outfl_String, dat_List, OptionsPattern[]] := Module[
   {tmp,
    hem = OptionValue[Hemisphere],
    erange = OptionValue[Eccentricity],
    fstat = OptionValue[FStat],
    fl = OpenWrite[outfl, BinaryFormat → True]},
   dat = If[(hem === Automatic && Mean[dat[[All, 3]]] < 0) || hem == RH,
     Map[# * {1, 1, -1, 1, 1} &, dat],
     dat];
   tmp = Map[
     {#1[[1]], #1[[2]], #[[3]], #[[4]],
       If[erange[[1]] ≤ Abs[#1[[4]]] ≤ erange[[2]] && #[[5]] > fstat[[1]],
        If[#[[5]] > fstat[[2]], 2,
         (#[[5]] - fstat[[1]]) * 2 / (fstat[[2]] - fstat[[1]])],
        0]} &,
     dat];
   If[OptionValue[PrintFC] === True,
    Module[{fc = First[Sort[Select[tmp, InV1Q], #1[[1]] < #2[[1]] &]]},
     Print["Foveal Confluence" → Ordering[tmp, 1, Norm[#1 - fc] < Norm[#2 - fc] &]]]];
   If[OptionValue[PrintPD] === True,
    Module[{pd = Last[Sort[Select[tmp, InV1Q], #1[[1]] < #2[[1]] &]]},
     Print[
      "Peripheral Diffluence" → Ordering[tmp, 1, Norm[#1 - pd] < Norm[#2 - pd] &]]]];
   BinaryWrite[fl, Length@tmp, "Integer32"];
   BinaryWrite[
    fl,
    Flatten@tmp,
    "Real64"];
   Close[fl]];
DeclareKeyword[PrintFC, PrintPD];
Options[WriteSimStartFile] =
  {Hemisphere → Automatic, Eccentricity → {1.25, 8.75}, FStat → {28, 50},
   PrintFC → False, PrintPD → False};
```

```
Clear[ReadSimResultFile];
ReadSimResultFile[infl_String, OptionsPattern[]] := Module[
   {hem = OptionValue[Hemisphere],
    fstat = OptionValue[FStat],
    fl = OpenRead[infl, BinaryFormat → True],
    tmp},
   tmp = BinaryReadList[
     fl,
     Table["Real64", {5}],
     BinaryRead[fl, "Integer32"]];
   Close[fl];
   Map[
    Append[
      #[[1 ;; 4]],
      If[fstat === None,
       #[[5]],
       Which[
        #[[5]] ≤ 0, 0,
        #[[5]] ≥ 2, fstat[[2]],
        True, #[[5]] * (fstat[[2]] - fstat[[1]]) / 2 + fstat[[1]]]]] &,
    If[(hem === Automatic && Mean[tmp[[All, 3]]] < 0) || hem === RH,
     Map[# * {1, 1, -1, 1, 1} &, tmp],
     tmp]]];
Options[ReadSimResultFile] = {Hemisphere → Automatic, FStat → {16, 32}};
```

1.9.5. Smoothing Predicted/Aggregate data

First, a basic Gaussian smoother, which we use as a point of comparison against the Voronoi smoothing method.

```
SmoothPredictedModel[inv_, σ_: 0.02] := With[
   {V2Hull = With[
      {a = Select[
          inv[[All, {1, 2, 5}]],
          NumberQ[#[[3]]] && Abs[#[[3]]] == 2 &
         ][[All, 1 ;; 2]]},
      Part[
       a,
       Append[#, First@#] &@ConvexHull[a]]],
    V3Hull = Module[
      {a = Select[
          inv[[All, {1, 2, 5}]],
          NumberQ[#[[3]]] && Abs[#[[3]]] == 3 &
         ][[All, 1 ;; 2]]},
      Part[
       a,
       Append[#, First@#] &@ConvexHull[a]]],
    usable = Select[inv, NumberQ[#[[5]]] &]},
   With[
    {gauss = Transpose[
       ParallelMap[
        Function[{pt},
         With[
          {row = Map[
```

```
          Compile[{{other, _Real, 1}},
           With[{nrm = (other[[1]] - pt[[1]])^2 + (other[[2]] - pt[[2]])^2},
            If[nrm > 16.0 * σ^2, 0.0, Exp[-nrm / (2.0 * σ^2)]]]],
          usable[[All, 1 ;; 2]]]},
       {Select[row, Compile[{{r, _Real}}, r > 0.0]],
        Select[Range[Length@row],
         Compile[{{i, _Integer}}, row[[i]] > 0.0]]}]],
    usable[[All, 1 ;; 2]]]],
  filledAreas = ParallelMap[
    Function[{pt},
     Which[
      And[
       Or[! NumberQ[pt[[3]]], Abs[pt[[3]]] < 1, Abs[pt[[3]]] > 3],
       PointInPolygonQ[pt[[1 ;; 2]], V2Hull, BoundaryStyle → True]],
      Append[pt[[1 ;; 2]], 2],
      And[
       NumberQ[pt[[3]]], Abs[pt[[3]]] == 2,
       ! PointInPolygonQ[pt[[1 ;; 2]], V2Hull, BoundaryStyle → True],
       PointInPolygonQ[pt[[1 ;; 2]], V3Hull, BoundaryStyle → True]],
      Append[pt[[1 ;; 2]], 3],
      True, pt]],
    usable[[All, {1, 2, 5}]]]]},
 With[
  {smoothAreas = MapThread[
     Compile[{{gaus, _Real, 1}, {idx, _Integer, 1}},
      Round[
       Divide[
        Dot[Abs[usable[[idx, 5]]], gaus],
        Total[gaus]]]],
     gauss],
   smoothAngle = MapThread[
     Compile[{{gaus, _Real, 1}, {idx, _Integer, 1}},
      Divide[
       Dot[Abs[usable[[idx, 3]]], gaus],
       Total[gaus]]],
     gauss],
   smoothEccen = MapThread[
     Compile[{{gaus, _Real, 1}, {idx, _Integer, 1}},
      Divide[
       Dot[Abs[usable[[idx, 4]]], gaus],
       Total[gaus]]],
     gauss]},
  Cases[
   MapThread[
    {#4[[1]], #4[[2]], #1, #2, #3} &,
    {smoothAngle, smoothEccen, smoothAreas, usable}],
   {_?NumberQ, _?NumberQ,
    θ_ /; (NumberQ[θ] && 0 ≤ θ ≤ 180),
    r_ /; (NumberQ[r] && 0 ≤ r ≤ 90),
    a_ /; (NumberQ[a] && 1 ≤ a ≤ 4)}]]]];
```

```
Clear[WithUniformPolarAngle];
WithUniformPolarAngle[sp_] := With[
   {ids = Table[Flatten[Position[sp[[All, 5]], k]], {k, 1, 4}]},
   With[
    {dist = Table[EmpiricalDistribution[sp[[ids[[k]], 3]]], {k, 1, 4}]},
    Map[
     Function[{row},
      List[
       row[[1]], row[[2]],
       180.0 * CDF[dist[[Round[row[[5]]]]], row[[3]]],
       row[[4]], Round[row[[5]]]]],
     sp]]];
```

The VoronoiSmooth function is an alternative to Gaussian smoothing. It uses the Voronoi registration method to take the result of a simulation (ie, the unsmoothed corrected topology) and registers it to the model using the VoronoiRegister function. In this way, the result will be VoronoiRegister-like, but will begin with the spring-simulation result, thus should have the extrapolation advantage of the spring simulation along with the accuracy of the Voronoi registration.
If the option While is specified, then the Steps option is ignored; otherwise, the registration is run for Steps steps. If DelaunayTriangulation is not provided, then LocalDelaunayTriangulation is used.

```
DeclareKeyword[Steps];
Options[VoronoiSmooth] = {
   Function :> SchiraFunction[SchiraModel[]],
   Steps -> 25,
   While -> Automatic,
   DelaunayTriangulation :> LocalDelaunayTriangulation};
VoronoiSmooth[simResult_List, OptionsPattern[]] := With[
   {dt = OptionValue[DelaunayTriangulation],
    fn = OptionValue[Function],
    while = Replace[
      OptionValue[While],
      {Automatic :> With[
         {steps = OptionValue[Steps]},
         Function[{step, new, old}, step < steps]]}]},
   VoronoiRegister[
    simResult,
    Table[
      With[{z = fn[#[[1]], #[[2]], k]}, {Re@z, Im@z}],
      {k, 1, 4}
     ] &,
    DelaunayTriangulation -> dt,
    While -> while]];
```

1.9.6. Writing Jobs, the input for simulations

```
Clear[ExportJob];
DeclareKeyword[FixFC, FixPD, OverwriteExistingJob];
Options[ExportJob] = {
   Directory -> $SpringsDirectory,
   Hemisphere -> Automatic,
```

```
    Eccentricity → {1.25, 8.75},
    FStat → {28, Automatic},
    Options → None,
    OverwriteExistingJob → False,
    SchiraModel → SchiraModel[],
    FixFC → False,
    FixPD → False};
ExportJob::permission = "You do not have permission to write/create `1`";
ExportJob[name_String, data_List, OptionsPattern[]] := With[
    {dir = OptionValue[Directory] <> "/",
     opts = Replace[OptionValue[Options], None → {}],
     fstat = Replace[
       OptionValue[FStat],
       {None :→ {0, Max[data[[All, 5]]]},
        {a_, b_} /; And[NumberQ[a], NumberQ[b], 0 ≤ a < b] :→ {a, b},
        {a_, Automatic} /; And[NumberQ[a], a ≥ 0] :→ {a, Max[data[[All, 5]]]},
        _ :→ Die["Bad FStat argument"]}],
     ow = OptionValue[OverwriteExistingJob]},
    Which[
     And[
      ow == False,
      DirectoryQ[dir <> "jobs/" <> name],
      FileExistsQ[dir <> "jobs/" <> name <> "/start.bin"]],
     True,
     And[
      ! DirectoryQ[dir <> "jobs/" <> name],
      $Failed === CreateDirectory[dir <> "jobs/" <> name]],
     (Message[ExportJob::permission, dir <> "jobs/" <> name];
      $Failed),
     $Failed === WriteSimStartFile[
       dir <> "jobs/" <> name <> "/start.bin",
       data,
       Eccentricity → OptionValue[Eccentricity],
       FStat → fstat],
     (Message[ExportJob::permission, dir <> "jobs/" <> name <> "/start.bin"];
      $Failed),
     ! StringQ[
       ExportSchiraParameters[
        dir <> "jobs/" <> name <> "/initial-schira-params.txt",
        OptionValue[SchiraModel]]],
     (Message[ExportJob::permission,
       dir <> "jobs/" <> name <> "/initial-schira-params.txt"];
      $Failed),
     $Failed === Export[
       dir <> "jobs/" <> name <> "/run.sh",
       Apply[
        StringJoin,
        Flatten[
         {"#! /bin/bash\n",
          "src/schira \\\n",
          "  --threads=8 \\\n",
          "   --journal=results/" <> name, " \\\n",
          "   --schira-param-file=jobs/",
          name, "/initial-schira-params.txt \\\n",
          "   --schira-minimize-stepsize=0 \\\n",
```

```
            "   --data-minimize-stepsize=0.005 \\\n",
            "   --schira-cutoff=0.25 \\\n",
            "   --spring-cutoff=0.015 \\\n",
            "   --schira-strength=5.0 \\\n",
            "   --spring-strength=1.0 \\\n",
            "   --initial-energy=5.0 \\\n",
            "   --dampen=0.999 \\\n",
            "   --annealing-iterations=4 \\\n",
            "   --steps=5000",
            If[OptionValue[FixFC] === True,
             Module[
              {fc = First[Sort[Select[data, InV1Q], #1[[1]] < #2[[1]] &]]},
              {" \\\n   --fix-foveal-confluence=",
               ToString[First[Ordering[data, 1, Norm[#1 - fc] < Norm[#2 - fc] &]]]}],
             {}],
            If[OptionValue[FixPD] === True,
             Module[
              {pd = First[Sort[Select[data, InV1Q], #1[[1]] > #2[[1]] &]]},
              {" \\\n   --fix-peripheral-diffluence=",
               ToString[First[Ordering[data, 1, Norm[#1 - pd] < Norm[#2 - pd] &]]]}],
             {}],
            Map[
             {" \\\n   ", #} &,
             opts],
            " \\\n   \"$@\" \\\n",
            "   jobs/", name, "/start.bin \\\n",
            "   results/", name, "/result.bin\n"}]],
         "Text"],
       (Message[ExportJob::permission, dir <> "/jobs/" <> name <> "/run.sh"];
        $Failed),
       True, dir <> "/jobs/" <> name]];
```

1.9.7. Reading Journals, the output of simulations

```
Clear[ReadJournal];
(* Symbols we want to protect (e.g. Options) *)
DeclareKeyword[
  InitialSchiraParameters,
  MinimizedSchiraParameters,
  InitialSchiraModel,
  MinimizedSchiraModel,
  SchiraParameters,
  SchiraPotential,
  Trajectory,
  StepsPerCycle,
  Result,
  VoronoiResult,
  Prediction,
  CacheFile,
  SmoothPrediction,
  SmoothPredictionFull,
  SmoothPredictionVoronoi,
  SmoothPredictionVoronoiFull,
  SmoothPredictionGaussian,
```

```
  SmoothPredictionGaussianFull,
  SimulationParameters,
  VoronoiSteps];
(* Messages for ReadJournal *)
ReadJournal::nosuchdir = "No such directory: `1`";
ReadJournal::missing = "Journal component `1` is missing";
ReadJournal::badhem = "Unrecognized hemisphere: `1`";
ReadJournal::badfmt = "Bad file format for component `1`";
(* JournalQ tests to see if something is a journal *)
JournalQ[x_] := False;
(* The ReadJournal definition itself *)
ReadJournal[filename_String, OptionsPattern[]] :=
  Message[ReadJournal::nosuchdir, filename] /; Not[DirectoryQ[filename]];
ReadJournal[filename_String, OptionsPattern[]] := Module[
   {hem = Replace[
      OptionValue[Hemisphere],
      Automatic → Replace[
        StringCases[
         Last[StringSplit[filename, "/"]],
         {"lh" → LH, "left" → LH, "rh" → RH, "right" → RH, "mh" → LH},
         IgnoreCase → True],
        {{} → Indeterminate,
         x : {LH | RH} ⧴ x[[1]],
         _ → Indeterminate}]],
    fst = OptionValue[FStat],
    sp = OptionValue[SmoothPrediction],
    doCache = 0,
    key},
   (* Sanity checks... *)
   Which[
    hem =!= LH && hem =!= RH, (
     Message[ReadJournal::badhem, hem];
     $Failed),
    ! FileExistsQ[filename <> "/options.txt"], (
     Message[ReadJournal::missing, Options];
     $Failed),
    ! FileExistsQ[filename <> "/result.bin"], (
     Message[ReadJournal::missing, Options];
     $Failed),
    True, (
     key[Hemisphere] = hem;
     (* We get the options right away... *)
     key[Options] = Get[filename <> "/options.txt"];
     (* and process them *)
     ReplaceAll[
      key[Options],
      (a_ → {b_, auto_}) ⧴ (
        key[a] = b;
        key[a, Automatic] = ! b)];
     key[Cycles] = key["annealing-iterations"];
     key[StepsPerCycle] = key["steps"];
     If[
      And[
       FileExistsQ[filename <> "/minimized-schira-params.txt"],
       OrderedQ[
```

```
    {FileDate[filename <> "/initial-schira-params.txt"],
     FileDate[filename <> "/minimized-schira-params.txt"]}]],
  key[MinimizedSchiraParameters] := (
    key[MinimizedSchiraParameters] = UsingSchiraParams[
      filename <> "/minimized-schira-params.txt",
      Map[Apply[Rule, #] &, $SchiraParams]]),
  key[MinimizedSchiraParameters] = None];
If[FileExistsQ[filename <> "/initial-schira-params.txt"],
  key[InitialSchiraParameters] := (
    key[InitialSchiraParameters] = UsingSchiraParams[
      filename <> "/initial-schira-params.txt",
      Map[Apply[Rule, #] &, $SchiraParams]]),
  key[InitialSchiraParameters] := (
    Message[ReadJournal::missing, InitialSchiraParameters];
    None)];
key[SchiraParameters] := Quiet[
   Module[
    {init = key[InitialSchiraParameters],
     min = key[MinimizedSchiraParameters]},
    Which[
     min === None && init === None, (
      key[SchiraParameters] := (
        Message[ReadJournal::missing, SchiraParameters];
        None);
      key[SchiraParameters]),
     min === None, key[SchiraParameters] = init,
     True, key[SchiraParameters] = min]]];
key[SchiraModel] :=
  (key[SchiraModel] = Apply[SchiraModel, key[SchiraParameters]]);
key[InitialSchiraModel] := (key[InitialSchiraModel] =
    Apply[SchiraModel, key[InitialSchiraParameters]]);
key[MinimizedSchiraModel] := (key[MinimizedSchiraModel] =
    Apply[SchiraModel, key[MinimizedSchiraParameters]]);
(* result file... *)
key[Result] := (key[Result] =
    ReadSimResultFile[filename <> "/result.bin", Hemisphere → LH, FStat → fst]);
If[FileExistsQ[filename <> "/initial_schira_pe.bin"],
  key[SchiraPotential] := (key[SchiraPotential] =
     ReadSimResultFile[filename <> "/initial_schira_pe.bin", FStat → None])];
key[Min] := key[Result];
key[Min, k_Integer /; k > 0] := Module[
   {minfl = filename <> "/min" <> ToString[k] <> ".bin"},
   If[! FileExistsQ[minfl],
    (Message[ReadJournal::missing, "Min[" <> ToString[k] <> "]"]; $Failed),
    (key[Min, k] = ReadSimResultFile[minfl, Hemisphere → LH, FStat → fst])]];
key[Trajectory, k_Integer /; k > 0] := Module[
   {flnm = Function[{t},
      StringJoin[
       filename, "/t=", IntegerString[k - 1, 10, 2], ".",
       IntegerString[t, 10, Floor[Log[10.0, key[StepsPerCycle] / 1000.0]] + 1],
       ".bin"]],
    ok = True},
   key[Trajectory, k] = Module[
     {dat = Table[
        Which[
```

```
!ok, $Failed,
!FileExistsQ[flnm[t]], (
 Message[ReadJournal::missing, Trajectory[k, t]];
 ok = False;
 $Failed),
True, Module[
 {fl = OpenRead[flnm[t], BinaryFormat → True], tmp},
 tmp = Map[
   Partition[#, 2] &,
   BinaryReadList[fl,
    Table["Real64", {2 * BinaryRead[fl, "Integer32"]}]]];
 Close[fl];
 If[Length[tmp] < 100,
  (Message[ReadJournal::badfmt, Trajectory[k, t]];
   ok = False;
   $Failed),
  tmp]]],
{t, 0, Floor[(key[StepsPerCycle] - 1) / 1000], 1}]},
If[ok, Apply[Join, dat], $Failed]]];
key[Trajectory] := Set[
  key[Trajectory],
  Apply[Join, Table[key[Trajectory, k], {k, 1, key[Cycles]}]]];
key[Prediction] := CompoundExpression[
  Set[
   key[Prediction],
   Select[
    Quiet[
     With[{f = InverseSchiraFunction[key[InitialSchiraModel]]},
      MapThread[
       Join[
         #1[[1 ;; 2]],
         Replace[
          f[#2[[1]], #2[[2]]],
          (Undefined | {_, x_ /; x > 90, _}) →
           {Undefined, Undefined, Undefined}]] &,
       {LocalAnatomy, key[Result]}]]]],
    NumberQ[#[[5]]] &]],
  doCache += 1;
  If[doCache == 2,
   Block[
    {prediction = key[Prediction],
     smoothPrediction = key[SmoothPrediction]},
    DumpSave[key[CacheFile], {prediction, smoothPrediction}]]],
  key[Prediction]];
key[SmoothPredictionGaussian] := CompoundExpression[
  Set[
   key[SmoothPredictionGaussian],
   SmoothPredictedModel[key[Prediction]]],
  doCache += 1;
  If[doCache == 2,
   Block[
    {prediction = key[Prediction],
     smoothPrediction = key[SmoothPredictionGaussian]},
    DumpSave[key[CacheFile], {prediction, smoothPrediction}]]],
  key[SmoothPredictionGaussian]];
```

```
      key[VoronoiResult] := With[
        {fn = SchiraFunction[key[SchiraModel]],
         steps = OptionValue[VoronoiSteps]},
        key[VoronoiResult] = MapThread[
          Join,
          {VoronoiSmooth[
            key[Result],
            Function → fn,
            Steps → steps],
           key[Result][[All, 3 ;; All]]}]];
      key[VoronoiResult, steps_] := With[
        {fn = SchiraFunction[key[SchiraModel]]},
        key[VoronoiResult] = MapThread[
          Join,
          {VoronoiSmooth[
            key[Result],
            Function → fn,
            Steps → steps],
           key[Result][[All, 3 ;; All]]}]];
      key[SmoothPredictionVoronoi] := With[
        {ifn = InverseSchiraFunction[key[SchiraModel]]},
        Set[
         key[SmoothPredictionVoronoi],
         Quiet[
          MapThread[
           Join[
             #1[[1 ;; 2]],
             Replace[
              ifn[#2[[1]], #2[[2]]],
              (Undefined | {_, x_ /; x > 90, _}) → {0, 0, 0}]] &,
           {LocalAnatomy, key[VoronoiResult]}]]]]];
      key[SmoothPrediction] := key[sp];
      key[SmoothPredictionFull] := With[{tol = 0.0000001},
        Set[
         key[SmoothPredictionFull],
         Map[
          If[Length@# == 2, #[[1]], Join[#[[1, 1 ;; 2]], {0, 0, 0}]] &,
          Split[
           Sort[
            Join[
             key[SmoothPrediction],
             LocalAnatomy],
            Which[
              #1[[1]] - #2[[1]] < -tol, True,
              #1[[1]] - #2[[1]] > tol, False,
              #1[[2]] - #2[[2]] < -tol, True,
              #1[[2]] - #2[[2]] > tol, False,
              Length[#1] > Length[#2], True,
              True, False
             ] &],
           With[{tmp = Sqrt[2.0] * tol},
            (Norm[#1[[1 ;; 2]] - #2[[1 ;; 2]]] < tmp) &]]]]];
      JournalQ[key] = True;
      key)]];
Options[ReadJournal] = {
```

```
Hemisphere → Automatic,
FStat → {20, 30},
SmoothPrediction → SmoothPredictionGaussian,
VoronoiSteps → 5};
```

1.9.8. Reporting LOO Errors

This code block generates a table reporting the signed and absolute leave-one-out errors in the prediction.

```
ReportLOOErrors[data_, looPredictions_List, {eccMn_, eccMx_}] := With[
   {looErr = Table[
      With[
       {pred = looPredictions[[k]],
        lo = data[Data, All, k]},
       Flatten[
        Reap[
          MapThread[
           {pv, lv} Ì If[
             And[
              NumberQ[pv[[5]]] && 0 < pv[[5]] < 4 && eccMn ≤ lv[[4]] ≤ eccMx,
              Block[
               {x = lv[[1]], y = lv[[2]],
                θ = lv[[3]], r = lv[[4]], f = lv[[5]], c = None},
               data[MeanFilter]]],
             Sow[{{pv[[3]], lv[[3]]}, {pv[[4]], lv[[4]]}, pv[[5]]}]],
           {pred, MatchOrder[lo → pred]}]
         ][[2]],
        1]],
      {k, 1, Length@looPredictions}]},
   With[
    {eccerrTmp = Reap[
        Scan[
         Sow[#1⟦1⟧, {0, Round[#1⟦2⟧]}] &,
         Flatten[looErr⟦All, All, 2 ;; 3⟧, 1]],
        {0, 1, 2, 3},
        Sequence @@ #2 &
       ]⟦2⟧,
     polerrTmp = Reap[
        Scan[
         Sow[#1⟦1⟧, {0, Round[#1⟦2⟧]}] &,
         Flatten[looErr⟦All, All, {1, 3}⟧, 1]],
        {0, 1, 2, 3},
        Sequence @@ #2 &
       ]⟦2⟧},
    MatrixForm[
     Prepend[
      MapThread[
       {#1,
         Median[#2⟦All, 1⟧ - #2⟦All, 2⟧],
         Median[Abs[#2⟦All, 1⟧ - #2⟦All, 2⟧]],
         Median[#3⟦All, 1⟧ - #3⟦All, 2⟧],
         Median[Abs[#3⟦All, 1⟧ - #3⟦All, 2⟧]]} &,
       {{"V1", "V2", "V3", "All"},
        RotateLeft[eccerrTmp],
        RotateLeft[polerrTmp]}],
      {"Area",
       "\!\(\*SubscriptBox[\(ϵ\), \(ρ\)]\)",
       "|\!\(\*SubscriptBox[\(ϵ\), \(ρ\)]\)|",
       "\!\(\*SubscriptBox[\(ϵ\), \(θ\)]\)",
       "|\!\(\*SubscriptBox[\(ϵ\), \(θ\)]\)|"}]]]];
```

1.10. Figures Setup

1.10.1. Default Values

These are some convenient functions for things like color scales that are used throughout.

```
$PolarAngleColorFn = Function[{val},
   Blend[
    {Blue, Cyan, Darker[Green], Yellow, Red},
    val / 180.0]];
$EccentricityColorFn = Function[{val},
   If[val > 20.0,
    White,
    Blend[
     {Blue, Magenta, Darker@Red, Red, Lighter@Yellow, White},
     val / 20.0]]];
$Data10FSAverageOutline = Black;
$Data10CorrectedOutline = Red;
$Data20FSAverageOutline = Black;
$Data20CorrectedOutline = Red;
$DataLongFSAverageOutline = Black;
$DataLongCorrectedOutline = Red;
SelectPolarAngle[data_List, minFValue_: 22.0, eccenRange_: {0, 20.0}] := Select[
    data,
    And[
      #[[5]] > minFValue,
      0 < #[[3]] < 180,
      #[[3]] ≠ 90,
      eccenRange[[1]] < #[[4]] ≤ eccenRange[[2]]] &
   ][[All, 1 ;; 3]];
SelectEccentricity[data_List,
   minFValue_: 22.0, eccenRange_: {0, 20.0}] := Select[
    data,
    And[
      #[[5]] > minFValue,
      0 < #[[3]] < 180,
      #[[3]] ≠ 90,
      eccenRange[[1]] < #[[4]] ≤ eccenRange[[2]]] &
   ][[All, {1, 2, 4}]];
DistortData[data_List, anat_List] :=
  MapThread[Join[#2[[1 ;; 2]], #1[[3 ;; All]]] &, {data, anat}] /;
   Length@anat == Length@data;
```

1.10.2. Merging two different scales, such as LocalAnatomy and aggregate/prediction data

```
With[{ϵ=0.01,precision=0.0000001},
    Clear[MergeScales];
    MergeScales[argsSeq:(Rule[_List,_]..)] := With[
        {args={argsSeq}},
        With[
            {extrema = Map[
                {Min@#[[1,All,3]],Max@#[[1,All,3]]}&,
```

```
            args]},
        With[
            {funs = MapThread[
                Function[{arg,minmax},
                    Replace[
                        arg[[2]],
                        {Hue → (Hue[2./3.*(1.0 - (#-minmax[[1]])/(minmax[[2]]-minmax
                         x_List :→ (Blend[x,(#-minmax[[1]])/(minmax[[2]]-minmax[[1]]
                {args,extrema}]},
            Module[{starts, range},
                {range, {starts}} = Module[{stack = -ϵ},
                    Reap[
                        Scan[
                            (stack+=ϵ;Sow[stack];stack+=#[[2]]-#[[1]])&,
                            extrema];
                        stack]];
                {Part[
                     Split[
                         Sort[
                             Flatten[
                                 MapThread[
                                     Function[{arg,idx,extreme,start},
                                         Map[
                                             List[
                                                 #[[1]],
                                                 #[[2]],
                                                 start+#[[3]]-extreme[[1]],
                                                 idx
                                             ]&,
                                             arg]],
                                     {args[[All,1]],Range[Length[args]],extrema,sta
                                 1],
                             Which[
                                 #1[[1]] - #2[[1]] < -precision, True,
                                 #1[[1]] - #2[[1]] > precision, False,
                                 #1[[2]] - #2[[2]] < -precision, True,
                                 #1[[2]] - #2[[2]] > precision, False,
                                 #1[[4]] < #2[[4]], True,
                                 #1[[4]] > #2[[4]], False,
                                 True,True
                            ]&],
                         And[
                             Abs[#1[[1]]-#2[[1]]]<precision,
                             Abs[#1[[2]]-#2[[2]]]<precision
                         ]&],
                     All, 1, 1;;3],
                 Block[{val},
                     Function[{val},
                        Evaluate[
                            Apply[
                                Which,
                                Join[
                                    Flatten[
                                        MapThread[
                                            Function[{minmax,start,fun},
                                                {start≤val≤(start+minmax[[2]]-minma
                                                Unevaluated[fun[(val-start+minmax[
                                            {extrema,starts,funs}]],
                                    {True,Black}]]]]]}]]]]];
```

```
Clear[Compare];
Compare[A_, B_, OptionsPattern[]] := With[
   {cmpFn = OptionValue[Order],
    a = If[Head[A] === Rule, A[[1]], A],
    b = If[Head[B] === Rule, B[[1]], B],
    aFn = If[Head[A] === Rule, A[[2]], Null],
    bFn = If[Head[B] === Rule, B[[2]], Null],
    outer = OptionValue[Outer],
    inner = OptionValue[Inner]},
   outer[
    NestWhile[
     Function[{lists},
      Which[
       Length[lists[[1]]] == 0, (
        If[bFn =!= Null, bFn[lists[[2, 1]]]];
        {{}, Rest[lists[[2]]]}),
       Length[lists[[2]]] == 0, (
        If[aFn =!= Null, aFn[lists[[1, 1]]]];
        {Rest[lists[[1]]], {}}),
       True, With[
        {cmp = cmpFn[lists[[1, 1]], lists[[2, 1]]]},
        Which[
         cmp > 0, (
          If[aFn =!= Null, aFn[lists[[1, 1]]]];
          {Rest[lists[[1]]], lists[[2]]}),
         cmp < 0, (
          If[bFn =!= Null, bFn[lists[[2, 1]]]];
          {lists[[1]], Rest[lists[[2]]]}),
         True, (
          inner[lists[[1, 1]], lists[[2, 1]]];
          {Rest[lists[[1]]], Rest[lists[[2]]]})]]]],
     With[
      {sortFn = (cmpFn[#1, #2] ≥ 0) &},
      {Sort[a, sortFn], Sort[b, sortFn]}],
     Or[Length[#[[1]]] > 0, Length[#[[2]]] > 0] &]]];
Options[Compare] = List[
   Order → Order,
   Outer → Function[{code}, Reap[code][[2, 1]], {HoldAll}],
   Inner → (Sow[{#1, #2}] &)];

VertexOrder[a_List, b_List, tol_?NumberQ /; tol > 0] := Which[
   a[[1]] - b[[1]] < -tol, 1,
   a[[1]] - b[[1]] > tol, -1,
   a[[2]] - b[[2]] < -tol, 1,
   a[[2]] - b[[2]] > tol, -1,
   True, 0];
VertexOrder[a_List, b_List] := Which[
   a[[1]] - b[[1]] < -0.000001, 1,
   a[[1]] - b[[1]] > 0.000001, -1,
   a[[2]] - b[[2]] < -0.000001, 1,
   a[[2]] - b[[2]] > 0.000001, -1,
   True, 0];
```

1.10.3. Polar Angle/Eccentricity Legends

These are two convenience functions for making polar angle and eccentricity legend plots (ie, the half-circle ones).

```
With[
  {ldat = Reverse[
     Table[
      If[Norm[{x, y}] > 10,
       {-1, -1},
       {If[x == y == 0, -1, N[(Pi / 2 - ArcTan[-x, y]) / Pi]], Norm[{x, y}] / 10.0}],
      {y, -10, 10, 0.1},
      {x, -10, 0, 0.1}]],
   rdat = Reverse[
     Table[
      If[Norm[{x, y}] > 10,
       {-1, -1},
       {If[x == y == 0, -1, N[(Pi / 2 - ArcTan[x, y]) / Pi]], Norm[{x, y}] / 10.0}],
      {y, -10, 10, 0.1},
      {x, 0, 10, 0.1}]]},
  PolarAngleLegend[hemiField : (Left | Right), OptionsPattern[]] := With[
    {sz = OptionValue[ImageSize],
     txt = OptionValue[Text],
     cfun = OptionValue[ColorFunction],
     cfunSc = OptionValue[ColorFunctionScaling],
     fsz = OptionValue[FontSize]},
    Apply[
     ArrayPlot,
     Join[
      {If[hemiField === Left, ldat, rdat][[All, All, 1]],
       ColorFunction → If[cfunSc,
         If[# < 0, White, cfun[#]] &,
         If[# < 0, White, cfun[180.0 * #]] &],
       ColorFunctionScaling → False,
       ImageSize → sz,
       Frame → None,
       Axes → None},
      If[txt,
       {Epilog → {
          Inset[
           Style["0°", FontColor → White, FontSize → fsz, FontFamily → "Arial"],
           ImageScaled[{If[hemiField === Left, 0.96, 0.04], 0.85}],
           ImageScaled[{If[hemiField === Left, 1, 0], 0.5}]],
          Inset[
           Style["180°", FontColor → White, FontSize → fsz, FontFamily → "Arial"],
           ImageScaled[{If[hemiField === Left, 0.96, 0.04], 0.15}],
           ImageScaled[{If[hemiField === Left, 1, 0], 0.5}]]}},
       {}]]]];
  EccentricityLegend[hemiField : (Left | Right), OptionsPattern[]] := With[
    {sz = OptionValue[ImageSize],
     txt = OptionValue[Text],
     cfun = OptionValue[ColorFunction],
     cfunSc = OptionValue[ColorFunctionScaling],
     fsz = OptionValue[FontSize]},
    Apply[
     ArrayPlot,
     Join[
```

```
      {If[hemiField === Left, ldat, rdat][[All, All, 2]],
       ColorFunction → If[cfunSc,
         If[# < 0, White, cfun[#]] &,
         If[# < 0, White, cfun[20.0 * #]] &],
       ColorFunctionScaling → False,
       ImageSize → sz,
       Frame → None,
       Axes → None},
      If[txt,
       {Epilog → {
          Inset[
           Style["0°", FontColor → White, FontSize → fsz, FontFamily → "Arial"],
           ImageScaled[{If[hemiField === Left, 0.96, 0.04], 0.5}],
           ImageScaled[{If[hemiField === Left, 1, 0], 0.5}]],
          Inset[
           Style["10°", FontColor → White, FontSize → fsz, FontFamily → "Arial"],
           If[hemiField === Left,
            ImageScaled[{0.66, 0.33}], ImageScaled[{0.33, 0.33}]],
           ImageScaled[{0.5, 0.5}]],
          Inset[
           Style["20°", FontColor → White, FontSize → fsz, FontFamily → "Arial"],
           ImageScaled[{If[hemiField === Left, 0.3, 0.7], 0.15}],
           ImageScaled[{If[hemiField === Left, 0, 1], 0}]]}},
       {}]]]]];
Options[PolarAngleLegend] = {
   ImageSize → 0.55 * 72,
   Text → True,
   FontSize → 8,
   ColorFunction :→ $PolarAngleColorFn,
   ColorFunctionScaling → False};
Options[EccentricityLegend] = {
   ImageSize → 0.55 * 72,
   Text → True,
   FontSize → 8,
   ColorFunction :→ $EccentricityColorFn,
   ColorFunctionScaling → False};
```

1.10.4. Inset Plot Functions

Methods for plotting the Schira line plot and the V1 Hull plot, without needing to remake them every time we use them.

```
Clear[SchiraLinePlotHash];
SchiraLinePlotHash[mdl_, Eccentricity] := Set[
   SchiraLinePlotHash[mdl, Eccentricity],
   SchiraLinePlot[
    mdl,
    EccentricityStyleFunction → ({Thick, Darker[$EccentricityColorFn[#]]} &),
    PolarAngleStyleFunction → ({Dashed, White} &),
    PolarAngleLines → {{0, 180}, None, {0, 180}},
    Axes → None,
    Frame → False]];
SchiraLinePlotHash[mdl_, PolarAngle] := Set[
   SchiraLinePlotHash[mdl, PolarAngle],
   SchiraLinePlot[
    mdl,
    PolarAngleStyleFunction → ({Thick, Darker[$PolarAngleColorFn[#]]} &),
    EccentricityStyleFunction → ({Dashed, White} &),
    EccentricityLines → {10, 90},
    Axes → None,
    Frame → False]];
Clear[V1HullPlot];
V1HullPlot := Set[
   V1HullPlot,
   ListPlot[
    V1Hull,
    Joined → True,
    PlotStyle → {Black, Dashed}]];
```

1.10.5. Cortical surface plots

Useful function for plotting images of the cortical surface.

```
CorticalPlot::badarg = "Bad argument `1`: `2`";
DeclareKeyword[BackgroundColorFunction,
  ShowCortex, CortexOutline, V1Outline, AnatomyData];
CorticalPlot[
   dat_List /; With[{d = Dimensions[dat]}, Length@d == 2 && d[[2]] ≥ 3],
   opts : OptionsPattern[CorticalPlot]
  ] := With[
   {colorFnScale = Replace[
      OptionValue[ColorFunctionScaling],
      Except[True | False] :> Message[CorticalPlot::badarg,
        "ColorFunctionScaling", "must be True or False"]],
    corticalColorFn = Replace[
      OptionValue[BackgroundColorFunction],
      Automatic → (If[# < -0.03051, LightGray, Gray] &)],
    showCortex = Replace[
      OptionValue[ShowCortex],
      Except[True | False] :>
       Message[CorticalPlot::badarg, "ShowCortex", "must be True or False"]],
    v1Outline = Replace[
      OptionValue[V1Outline],
      {(Automatic | True) → {Dashed, Black},
       (False | None) → None,
       x_ /; ! ListQ[x] :> {x}}],
```

```
    cortexOutline = Replace[
      OptionValue[CortexOutline],
      {(Automatic | True) → {Black, Thick},
       (False | None) → None,
       x_ /; ! ListQ[x] :→ {x}}],
    anat = Replace[
      OptionValue[AnatomyData],
      Automatic → LocalAnatomy],
    epi = Replace[OptionValue[Epilog], (None | False) → {}]},
   With[
    {colorFn = Replace[
       OptionValue[ColorFunction],
       Automatic :→ If[colorFnScale,
         Hue[2.0 / 3.0 * (1.0 - #)] &,
         With[{mn = Min[dat[[All, 3]]], mx = Max[dat[[All, 3]]]},
          Hue[2. / 3. * (1 - (# - mn) / (mx - mn))] &]]]},
    Module[{data, cfun},
     {data, cfun} = With[
       {tmpClrFn = If[colorFnScale,
          With[{mn = Min[dat[[All, 3]]], mx = Max[dat[[All, 3]]]},
           colorFn[(# - mn) / (mx - mn)] &],
          colorFn]},
       If[showCortex,
        MergeScales[dat → tmpClrFn, anat → corticalColorFn],
        {dat, tmpClrFn}]];
     ListDensityPlot[
      Select[data,
       PointInPolygonQ[#[[1 ;; 2]], LocalHull, BoundaryStyle → False] &],
      Evaluate[
       Apply[
        Sequence,
        FilterRules[
         Join[
          {Epilog → Apply[
             Join,
             {If[v1Outline === None, {}, {Append[v1Outline, Line[V1Hull]]}],
              If[cortexOutline ===
                None, {}, {Append[cortexOutline, Line[LocalHull]]}],
              {epi}}],
           ColorFunction → cfun,
           opts},
          Options[CorticalPlot]],
         Options[ListDensityPlot]]]]]]]];
Options[CorticalPlot] = Join[
   ReplaceAll[
    Options[ListDensityPlot],
    Map[
     Rule[#[[1]] → _, #[[1]] → #[[2]]] &,
     {ColorFunctionScaling → False,
      ColorFunction → Automatic,
      ImageSize → 3.25 * 72,
      AspectRatio → 1,
      PlotRange → Full,
      BaseStyle → Directive[10, FontFamily → "Arial"],
      Frame → False,
```

```
     Axes → False}]],
  {BackgroundColorFunction → Automatic,
   CortexOutline → None,
   ShowCortex → True,
   AnatomyData → Automatic,
   V1Outline → Automatic}];
```

2. Datasets

Datasets are declared using the DeclareDataset[] funciton, defined below. This function needs a list of subjects and a function (such as LoadSubjectVTK) that takes a subject's id (as passed to DeclareDataset) and hemisphere (LH or RH) and returns the subject's data.

For the datasets used in this paper, data for a single subject (which can be accessed via forms such as data10[Data]), are stored as n x 6 matrices where n is the number of vertices and the columns are {λ, ϕ, θ, ρ, f, c} where:
λ is the longitude of the vertex on the cortical surface
ϕ is the latitude of the vertex on the cortical surface
θ is the polar angle of the vertex
ρ is the eccentricity of the vertex
f is the f-value or confidence (which may have been rescaled)
c is the Gaussian curvature of the vertex

2.1. Dataset Infrastructure

```
DeclareKeyword[
  LoadFunction, Subjects, Filter, Data, Aggregate,
  Curvature, VoronoiPrediction, LOO, FStatRange, FilteredData];
Clear[DeclareDataset];
{$PolarAngle, $Eccentricity, $FStat, $Curvature} = {3, 4, 5, 6};
DeclareDataset[name_String,
   subjects_List, loadFun_, OptionsPattern[]] := Module[
   {data,
    filt = Block[{x, y, θ, r, f},
      Replace[
       OptionValue[Filter],
       None → True]],
    meanFilter = Block[{x, y, θ, r, f},
      Replace[
       OptionValue[MeanFilter],
       None → True]]},

   data[Subjects] := subjects;
   data[LoadFunction] := loadFun;

   (* If this is not redefined, then 20 is used as a Data threshold *)
   Block[{x, y, θ, r, f},
    data[Filter] := filt;
```

```
 data[MeanFilter] := meanFilter];

data[Data, hemi : (LH | RH), sub_String /; MemberQ[subjects, sub]] := Set[
  data[Data, hemi, sub],
  Select[
   loadFun[sub, hemi],
   Block[{x = #[[1]], y = #[[2]],
      θ = #[[3]], r = #[[4]], f = #[[5]]}, data[Filter]] &]];
data[Data, sub_String /; MemberQ[subjects, sub]] := {
  data[Data, RH, sub],
  data[Data, LH, sub]};
data[Data, All, sub_String /; MemberQ[subjects, sub]] := Set[
  data[Data, All, sub],
  MeanJointMap[
   data[Data, sub],
   data[MeanFilter],
   f]];
data[Data] := (data[Data] = Map[data[Data, #] &, subjects]);
(* Integer subject support *)
data[Data, hemi : (LH | RH), sub_Integer /; 1 ≤ sub ≤ Length[subjects]] :=
 data[Data, hemi, subjects[[sub]]];
data[Data, All, sub_Integer /; 1 ≤ sub ≤ Length[subjects]] :=
 data[Data, All, subjects[[sub]]];
data[Data, sub_Integer /; 1 ≤ sub ≤ Length[subjects]] :=
 data[Data, subjects[[sub]]];
(* All subject versions *)
data[Data, hemi : (LH | RH | All)] := Map[data[Data, hemi, #] &, subjects];

data[Aggregate, hemi : (LH | RH)] := Set[
  data[Aggregate, hemi],
  MeanJointMap[
   data[Data, hemi],
   data[MeanFilter],
   f]];
data[Aggregate] := (data[Aggregate] = MeanJointMap[
    Join[data[Data, LH], data[Data, RH]],
    data[MeanFilter],
    f]);
data[Aggregate,
  type : (PolarAngle | Eccentricity | FStat), hemi : (LH | RH)] := Which[
  type === PolarAngle, data[Aggregate, hemi][[All, 1 ;; 3]],
  type === Eccentricity, data[Aggregate, hemi][[All, {1, 2, 4}]],
  True, data[Aggregate, hemi][[All, {1, 2, 5}]]];
data[Aggregate, type : (PolarAngle | Eccentricity | FStat)] := Which[
  type === PolarAngle, data[Aggregate][[All, 1 ;; 3]],
  type === Eccentricity, data[Aggregate][[All, {1, 2, 4}]],
  True, data[Aggregate][[All, {1, 2, 5}]]];

data[Aggregate, LOO[sub_Integer /; 1 ≤ sub ≤ Length[subjects]]] := Set[
  data[Aggregate, LOO[sub]],
  MeanJointMap[
   Flatten[
    Delete[data[Data], sub],
    1],
```

```
    Evaluate[Block[{x, y, θ, r, f}, data[MeanFilter]]],
    f]];
data[Aggregate, LOO[sub_String /; MemberQ[subjects, sub]]] :=
 data[Aggregate, LOO[Position[subjects , sub][[1, 1]]]];
data[Aggregate, LOO[sub_Integer /; 1 ≤ sub ≤ Length[subjects]],
   hemi : (LH | RH)] := Set[
   data[Aggregate, LOO[sub], hemi],
   MeanJointMap[
    Delete[data[Data, hemi], sub],
    Evaluate[Block[{x, y, θ, r, f}, data[MeanFilter]]],
    f]];
data[Aggregate, LOO[sub_String /; MemberQ[subjects, sub]], hemi : (LH | RH)] :=
 data[Aggregate, LOO[Position[subjects , sub][[1, 1]]], hemi];

(* The Voronoi registration of the simulation to a Schira model *)
data[VoronoiRegister, mdl_SchiraModelObject, args___] := Set[
   data[VoronoiRegister, mdl, args],
   VoronoiRegister[
    data[Aggregate],
    SchiraToVoronoiFunction[SchiraFunction[mdl]],
    args,
    DelaunayTriangulation → LocalDelaunayTriangulation]];
data[VoronoiRegister, hemi : (LH | RH), mdl_SchiraModelObject, args___] := Set[
   data[VoronoiRegister, hemi, mdl, args],
   VoronoiRegister[
    data[Aggregate, hemi],
    SchiraToVoronoiFunction[SchiraFunction[mdl]],
    args,
    DelaunayTriangulation → LocalDelaunayTriangulation]];
data[VoronoiRegister,
   LOO[sub_String /; MemberQ[subjects, sub]],
   mdl_SchiraModelObject,
   args___
  ] := Set[
   data[VoronoiRegister, LOO[sub], mdl, args],
   VoronoiRegister[
    data[Aggregate, LOO[sub]],
    SchiraToVoronoiFunction[SchiraFunction[mdl]],
    args,
    DelaunayTriangulation → LocalDelaunayTriangulation]];
data[VoronoiRegister,
   hemi : (LH | RH),
   LOO[sub_String /; MemberQ[subjects, sub]],
   mdl_SchiraModelObject,
   args___
  ] := Set[
   data[VoronoiRegister, LOO[sub], mdl, args],
   VoronoiRegister[
    data[Aggregate, LOO[sub], hemi],
    SchiraToVoronoiFunction[SchiraFunction[mdl]],
    args,
    DelaunayTriangulation → LocalDelaunayTriangulation]];
data[
   VoronoiRegister,
```

```
    LOO[sub_Integer /; 1 ≤ sub ≤ Length[subjects]],
    mdl_SchiraModelObject,
    args___
   ] := data[VoronoiRegister, LOO[subjects[[sub]]], mdl, args];
  data[
    VoronoiRegister,
    hemi : (LH | RH),
    LOO[sub_Integer /; 1 ≤ sub ≤ Length[subjects]],
    mdl_SchiraModelObject,
    args__
   ] := data[VoronoiRegister, hemi, LOO[subjects[[sub]]], mdl, args];

  data[VoronoiPrediction, mdl_SchiraModelObject, args___] := Set[
    data[VoronoiPrediction, mdl, args],
    Select[
     Quiet[
      With[{f = InverseSchiraFunction[mdl]},
       MapThread[
        Join[
          #1[[1 ;; 2]],
          Replace[
           f[#2[[1]], #2[[2]]],
           (Undefined | {_, x_ /; x > 90, _}) → {0, 0, 0}]] &,
        {LocalAnatomy,
         data[VoronoiRegister, mdl, args]}]]],
     NumberQ[#[[5]]] &]];
  data[
    VoronoiPrediction,
    LOO[sub_ /; MemberQ[data[Subjects], sub]],
    mdl_SchiraModelObject,
    args___
   ] := Set[
    data[VoronoiPrediction, LOO[sub], mdl, args],
    Select[
     Quiet[
      With[{f = InverseSchiraFunction[mdl]},
       MapThread[
        Join[
          #1[[1 ;; 2]],
          Replace[
           f[#2[[1]], #2[[2]]],
           (Undefined | {_, x_ /; x > 90, _}) → {0, 0, 0}]] &,
        {LocalAnatomy,
         data[VoronoiRegister, LOO[sub], mdl, args]}]]],
     NumberQ[#[[5]]] &]];
  data[
    VoronoiPrediction,
    hemi : (LH | RH),
    LOO[sub_ /; MemberQ[data[Subjects], sub]],
    mdl_SchiraModelObject,
    args___
   ] := Set[
    data[VoronoiPrediction, LOO[sub], mdl, args],
    Select[
```

```
      Quiet[
       With[{f = InverseSchiraFunction[mdl]},
        MapThread[
         Join[
           #1[[1 ;; 2]],
           Replace[
            f[#2[[1]], #2[[2]]],
            (Undefined | {_, x_ /; x > 90, _}) → {0, 0, 0}]] &,
         {LocalAnatomy,
          data[VoronoiRegister, hemi, LOO[sub], mdl, args]}]]],
      NumberQ[#[[5]]] &]];
   data[
     VoronoiPrediction,
     LOO[sub_Integer /; 0 < sub ≤ Length[data[Subjects]]],
     mdl_SchiraModelObject,
     args___
    ] := data[VoronoiPrediction, LOO[subjects[[sub]]], mdl, args];
   data[
     VoronoiPrediction,
     hemi : (LH | RH),
     LOO[sub_Integer /; 0 < sub ≤ Length[data[Subjects]]],
     mdl_SchiraModelObject,
     args__
    ] := data[VoronoiPrediction, hemi, LOO[subjects[[sub]]], mdl, args];

   ToString[data] ^:= name;
   data];
Options[DeclareDataset] = Block[{f}, {Filter → None, MeanFilter → (f > 16)}];

SaveDataset[filename_String, dataset_] :=
  Block[{data = DownValues[Evaluate[dataset]]}, DumpSave[filename, data]];
LoadDataset[filename_String] := Block[{data}, Get[filename];
   If[! ListQ[data], (Print["Bad dataset file"]; Abort[])];
   With[{sym = Unique["test"]},
    Scan[Function[{rule}, Apply[SetDelayed, {Evaluate[Apply[Unevaluated,
          ReplaceAll[Extract[rule, {1, 1}, Hold], rule[[1, 1, 0]] → sym]]],
        Evaluate[Apply[Unevaluated, ReplaceAll[Extract[rule, {2}, Hold],
           rule[[1, 1, 0]] → sym]]]}]], data];
    sym]];
```

2.2. Dataset Declaration

2.2.1. 10° Dataset

Note that we declare no filters; this is because we want to examine the data before we

```
data10 = DeclareDataset[
   "10° dataset",
   Import[$SubjectDirectory <> "/subjects10.txt", "Words"],
   Function[{subject, hemisphere},
    LoadSubjectVTK[
     subject,
     hemisphere,
     Select → (ArcCos[Cos[#[[2]]] * Cos[-V1EllipseA - #[[1]]]] < Pi / 3 &)]],
   Filter → None,
   MeanFilter → None];
```

2.2.2. 20° Dataset

```
data20 = DeclareDataset[
   "20° dataset",
   Import[$SubjectDirectory <> "/subjects20.txt", "Words"],
   Function[{subject, hemisphere},
    LoadSubjectVTK[
     subject,
     hemisphere,
     RHInverted → False,
     Select → (ArcCos[Cos[#[[2]]] * Cos[-V1EllipseA - #[[1]]]] < Pi / 3 &),
     FStatSeparated → True]],
   Filter → None,
   MeanFilter → None];
```

2.2.3. Test/Retest Dataset

```
dataRetest = DeclareDataset[
   "Test/Retest dataset",
   Import[$SubjectDirectory <> "/subjectsRetest.txt", "Words"],
   Function[{subject, hemisphere},
    LoadSubjectVTK[
     subject,
     hemisphere,
     Select → (ArcCos[Cos[#[[2]]] * Cos[-V1EllipseA - #[[1]]]] < Pi / 3 &)]],
   Filter → None,
   MeanFilter → None];
```

3. F-Value Thresholds

In this section, we examine the appropriate thresholds for our datasets. First, we want to decide what f-value threshold we want to require of our individual subjects in order for their data to be included in the aggregate.

If you are using this notebook or the code in this notebook to examine a dataset other than our own, then it is critical that this section is examined in some depth. Based on your pre-processing, your retinotopy models, and the specifics of how you analyze and collect your data, these f-value thresholds may be very inappropriate for your dataset. The functions

and graphing options in this section will allow you to examine your dataset in order to find an appropriate f-value threshold.

Note that there are actually three steps in this process. First, the dataset’s Filter argument (which can be passed to DeclareDataset[] or redefined before the dataset’s Data field is evaluated, i.e., data10[Filter]:=(f>10)) determines which vertices are included in a subject’s raw data. We prefer to leave this filter as True (the default), meaning that all vertices, regardless of f-value, are included in the raw data. Second, there is the MeanFilter, which also may be passed or redefined. The MeanFilter determines which vertices are included in the calculation of the dataset’s Aggregates. During the calculation of the Aggregates, the values are weighted by their f-values.

One final thing; the image drawing cells here are marked as text; this is because you generally want to evaluate the rescaling cells but not the image drawing cells. The image-drawing cells take a long time to run, and probably aren’t ideal elements of the average startup sequence for this notebook.

3.1. Function for plotting differences in thresholds

This function actually produces plots of the thresholds; it can take awhile to run, but by varying the parameters, we can visualize the aggregates we get. Notice that this function does not actually reify the dataset[Aggregate] field, meaning that if you set the MeanFilter or the SubjectFilter for the dataset after running this function, you will still restrict the aggregate.

```
PlotThresholdedAggregates[dataset_,
   eccenPlotRange_, aggregateFMin_, springsCutoff_] := With[
   {agg = MeanJointMap[
      Flatten[dataset[Data], 1],
      f > aggregateFMin,
      f]},
   Graphics[
    Join[
     {Inset[
       CorticalPlot[
        SelectPolarAngle[
         agg,
         springsCutoff,
         eccenPlotRange],
        ImageSize → 3.25 * 72,
        CortexOutline → $Data10FSAverageOutline],
       ImageScaled[{0, 1}],
       ImageScaled[{0, 1}]],
      Inset[
       CorticalPlot[
        SelectEccentricity[
         agg,
         springsCutoff,
         eccenPlotRange],
```

```
        ImageSize → 3.25 * 72,
        CortexOutline → $Data10FSAverageOutline],
       ImageScaled[{0.5, 1}],
       ImageScaled[{0.5, 1}]],
      Inset[
       With[
        {selection = Select[
            agg,
            pt Ì eccenPlotRange[[1]] ≤ pt[[4]] ≤ eccenPlotRange[[2]]
           ][[All, {1, 2, 5}]]},
        With[
         {aggregateFMax = Max[selection[[All, 3]]]},
         CorticalPlot[
          selection,
          ColorFunctionScaling → False,
          ColorFunction → (
            Hue[2. / 3. * (1 - Max[
                  {0, (# - aggregateFMin) / (aggregateFMax - aggregateFMin)}])] &),
          ImageSize → 3.25 * 72,
          CortexOutline → $Data10FSAverageOutline]]],
       ImageScaled[{1, 1}],
       ImageScaled[{1, 1}]]},
     Table[
      Inset[
       SmoothHistogram[
        Select[agg[[All, dim]], # > 0 &],
        ImageSize → 3.25 * 72,
        Axes → None,
        Frame → {{True, False}, {True, False}},
        FrameLabel → {
          {"Probability Density", None},
          {Part[
            {"Polar Angle [deg]",
             "Eccentricity [deg]",
             "F-Value"},
            dim - 2],
           None}},
        PlotRange → {
          Part[
           {{0, 180}, {0, 12}, {0, 100}},
           dim - 2],
          Full},
        BaseStyle → Directive[10, FontFamily → "Arial"]],
       ImageScaled[{(dim - 3) / 2, 0}],
       ImageScaled[{(dim - 3) / 2, 0}]],
      {dim, 3, 5, 1}]],
    ImageSize → {10, 6} * 72,
    ImagePadding → 2000]];
```

3.2. 10° Dataset F-Value Threshold Exploration

Changing the third parameter (22 for our 10° dataset), you can examine different potential aggregates. We have found that the given aggregate produces low errors and provides a reasonable aggregate while excluding minimal measured data.

PlotThresholdedAggregates[data10, {0, 10}, 22, 20]

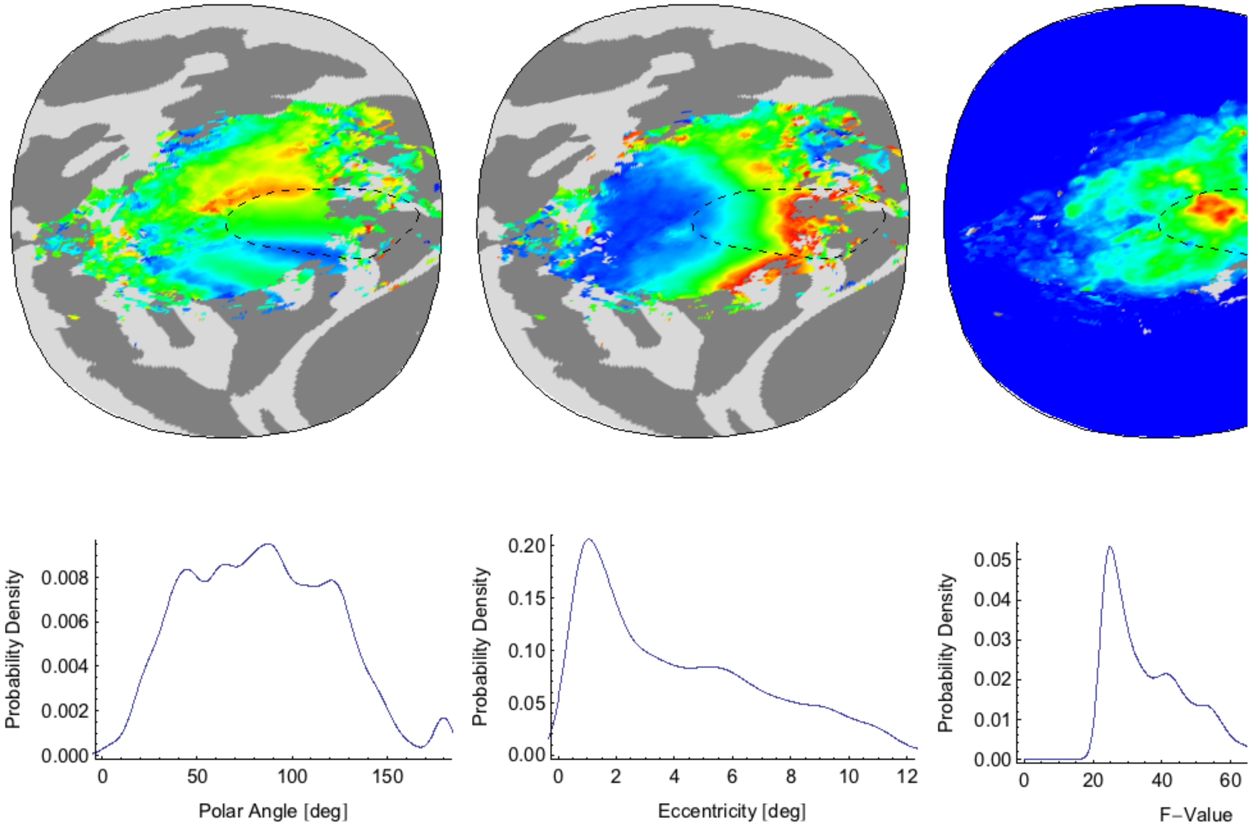

Based on these, we choose f > 22 (required of the aggregate) for a relatively good aggregate that errs on the side of including noisy data. Note that we can help mediate some of the noise in the next step, at least for the purposes of the simulated registration.

```
Block[{f}, data10[MeanFilter] := (f > 22)];
```

If one desires to weight the spring strength by fstat value, setting this to {fMin, fMax} will scale all the spring strengths to be between 0 and 2 (multiplied by the overall schira model strength, which for our simulations was 5) such that any vertex whose f-value is ≥ fMax is assigned a strength of 2, any vertex whose f-value is ≤ fMin is assigned a strength of 0, and any vertex whose f-values is between fMin and fMax is scaled linearly. For our purposes we did not find this to be necessary and did not weight vertices; by setting the fMax above the minimum f-value (per MeanFilter), this will accomplish this.

```
data10[FStatRange] = {10, 20};
```

3.3. 20° Dataset F-Value Threshold Exploration

Here we do the same for the 20° dataset, which was collected and analyzed using different methods thus will require a much different threshold.

PlotThresholdedAggregates[data20, {0, 20}, 12, 10]

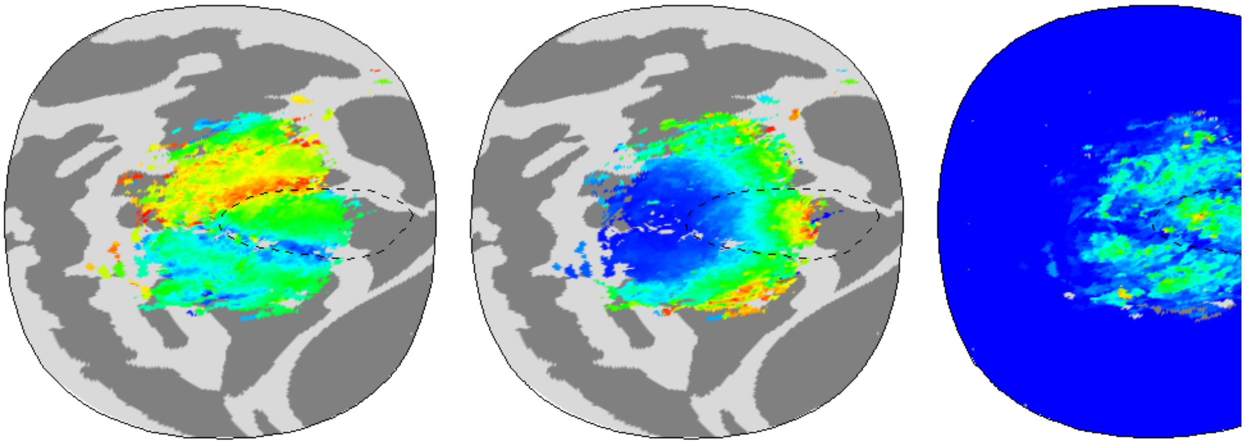

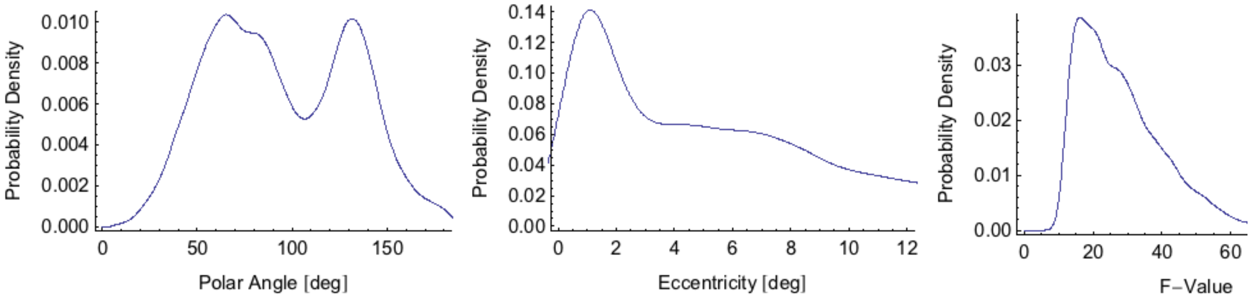

Based on these, we choose f > 12. For any simulations

```
Block[{f}, data20[MeanFilter] := (f > 12)];
```

```
data20[FStatRange] = {5, 10};
```

4. Spring Simulation Registration

4.1. Outputting Data for Simulation

Here, we output, into the jobs directory (which is set in section 1.1), the simulations to be run for morphing the cortical surface to the Schira model. Note that all simulations are outputted using the argument Hemisphere->LH, even the right hemisphere data. This is because the FSAverage-sym hemisphere is a left hemisphere.

4.1.1. 10° subjects

Output subjects for an overall aggregate fit into the jobs directory. The FStat argument here specifies the range of f-stats that get compressed to spring strengths of 0-2. For example, we used {20,30} for our FStat argument (see section 3.2), meaning that any vertex in the aggregate with an f-stat of 20 or lower gets a spring strength of 0, and every other vertex gets a spring strength of min{2, (f - 20) / 10}.

```
With[
  {data = data10,
   eccMn = 1.25,
   eccMx = 8.75},
  MapThread[
   {agg, name} Ì ExportJob[
     name,
     agg,
     SchiraModel → OurSchiraModel,
     FStat → data[FStatRange],
     Hemisphere → LH,
     OverwriteExistingJob → False],
   {{MatchPolarAngles[
      data[Aggregate] → Flatten[data[Data], 2],
      data[FStatRange][[2]],
      {eccMn, eccMx}],
     MatchPolarAngles[
      data[Aggregate, LH] → Flatten[data[Data, LH], 1],
      data[FStatRange][[2]],
      {eccMn, eccMx}],
     MatchPolarAngles[
      data[Aggregate, RH] → Flatten[data[Data, RH], 1],
      data[FStatRange][[2]],
      {eccMn, eccMx}]},
    {"aggregate", "aggregate-lh", "aggregate-rh"}}]];
```

4.1.2. Leave-One-Out simulations for the 10° dataset

Output individual LOO-type jobs into the jobs directory.
Note that LOO Aggregates don’t get rescaled by the rescaling functions called in section 3, so we have to rescale these manually.

```
With[
  {data = data10,
   eccMn = 1.25,
   eccMx = 8.75},
  Do[
   With[
    {agg = MatchPolarAngles[
       data[Aggregate, LOO[k]] → Flatten[Delete[data[Data], k], 2],
       data[FStatRange][[2]],
       {eccMn, eccMx}]},
    ExportJob[
     "loo-data10-subject" <> IntegerString[k, 10, 2],
     agg,
     SchiraModel → OurSchiraModel,
     FStat → data[FStatRange],
     Hemisphere → LH,
     OverwriteExistingJob → False]],
   {k, 1, Length@data[Subjects]}]];
```

4.2. Importing Spring Simulation Journals

Import the simulations (after they have been run; they should be run with journaling and be

placed in the results directory for this to work seamlessly). Again, all simulations are input as left hemispheres because the FSAverage-sym hemisphere is a left hemisphere.

```
sim10 = ReadJournal[
   $SpringsResultsDirectory <> "/aggregate",
   Hemisphere → LH,
   FStat → data10[FStatRange]];
```

```
simLH = ReadJournal[
   $SpringsResultsDirectory <> "/aggregate-lh",
   Hemisphere → LH,
   FStat → data10[FStatRange]];
simRH = ReadJournal[
   $SpringsResultsDirectory <> "/aggregate-rh",
   Hemisphere → LH,
   FStat → data10[FStatRange]];
```

```
simsLOO = Map[
   ReadJournal[
     #,
     Hemisphere → LH,
     FStat → data10[FStatRange]] &,
   FileNames[$SpringsResultsDirectory <> "/loo-data10-*"]];
```

4.3. Left versus Right Hemisphere Comparison

This shows the median absolute difference in the simulation-produced models of left and right hemisphere data; the first column is polar angle and the second is eccentricity. The frow are both-versus-LH, LH-versus-RH, and both-versus-RH

```
With[
  {errs = MapThread[
     Transpose[
       Map[
        {#[[1, 3]] - #[[2, 3]], #[[1, 4]] - #[[2, 4]]} &,
        Compare[#1[Prediction], #2[Prediction], Order → VertexOrder]]
      ] &,
     {{sim10, simLH, simRH},
      {simLH, simRH, sim10}}]},
  Map[
   Median[Abs[#]] &,
   errs,
   {2}]
 ] // MatrixForm
```

$$\begin{pmatrix} 1.20245 & 0.156459 \\ 1.58261 & 0.254376 \\ 1.32617 & 0.166837 \end{pmatrix}$$

5. Analyses of the Resulting Registration Data

Here, we generate several error reports; they should be self-explanatary. In general, they

are formatted as Type(PolarAngle|Eccentricity)->{region(1|2|3|All)->{median signed error, median absolute error}}.

5.1. Leave-One-Out Error Report for the 10° Datset

5.1.1. Spring Simulation Errors

```
ReportLOOErrors[
 data10,
 Map[#[SmoothPredictionFull] &, simsLOO],
 {1.25, 8.75}]
```

Area	ϵ_ρ	$\lvert\epsilon_\rho\rvert$	ϵ_θ	$\lvert\epsilon_\theta\rvert$
V1	0.259097	0.407562	2.0084	10.482
V2	0.0634423	0.341445	-3.1651	11.1177
V3	0.0950986	0.324936	3.34536	11.7285
All	0.147444	0.365449	0.579012	10.9429

5.1.2. Voronoi Registraion Errors

```
ReportLOOErrors[
 data10,
 data10[VoronoiPrediction, LOO[#], OurSchiraModel] & /@ data10[Subjects],
 {1.25, 8.75}]
```

Area	ϵ_ρ	$\lvert\epsilon_\rho\rvert$	ϵ_θ	$\lvert\epsilon_\theta\rvert$
V1	-0.0134286	0.792847	-0.469754	10.9703
V2	0.0442682	0.803277	-6.29698	15.6208
V3	0.294734	0.926893	3.42957	18.2345
All	0.0931622	0.834897	-1.23893	14.1289

5.2. Extension of 10° Dataset's Model Spring Registration to the 20° Dataset

5.2.1. Error in the spring registration model's prediction versus the 20° dataset aggregate

First, we calculate all the errors over the range 1.25° - 18.75°.

```
data20SSErrors =
  AggregateErrors[data20, sim10[SmoothPredictionGaussian], {1.25, 18.75}];
ReportAggregateErrors[data20SSErrors]
```

```
{PolarAngle → {All → {1.43451, 14.9683},
   1 → {-8.34411, 14.5685}, 2 → {11.6224, 15.7664}, 3 → {0.824473, 13.0797}},
 Eccentricity → {All → {-0.356903, 0.882095}, 1 → {-0.826636, 0.937429},
   2 → {-0.408766, 0.792058}, 3 → {0.114345, 0.661171}}}
```

Here, we do errors over the range 8.75° - 18.75°.

```
data20SSErrorsOuter = AggregateErrors[
   data20,
   sim10[SmoothPredictionFull],
   {8.75, 18.75}];
ReportAggregateErrors[data20SSErrorsOuter]
```

```
{PolarAngle → {All → {31.7252, 43.5846},
   1 → {-23.388, 27.5895}, 2 → {20.4848, 24.7553}, 3 → {2.4842, 14.6984}},
 Eccentricity → {All → {2.93308, 6.02071}, 1 → {-2.7701, 2.79628},
   2 → {-0.511348, 2.50193}, 3 → {1.99196, 2.85576}}}
```

And here we do errors over the range 1.25° - 8.75°.

```
data20SSErrorsInner = AggregateErrors[
   data20,
   sim10[SmoothPredictionFull],
   {1.25, 8.75}];
ReportAggregateErrors[data20SSErrorsInner]
```

```
{PolarAngle → {All → {20.5057, 27.5473},
   1 → {-4.09626, 11.1741}, 2 → {8.04281, 12.4087}, 3 → {0.3002, 12.5481}},
 Eccentricity → {All → {0.421052, 1.53197}, 1 → {-0.574508, 0.638006},
   2 → {-0.406267, 0.544438}, 3 → {-0.112814, 0.494671}}}
```

5.2.2. Error in the voronoi registration model's prediction versus the 20° dataset aggregate

First, we calculate all the errors over the range 1.25° - 18.75°.

```
data20VoronoiErrors = AggregateErrors[
   data20,
   data10[VoronoiPrediction, OurSchiraModel],
   {1.25, 18.75}];
ReportAggregateErrors[data20VoronoiErrors]
```

```
{PolarAngle → {All → {4.75455, 12.725},
   1 → {-3.60673, 8.85163}, 2 → {12.2631, 13.7344}, 3 → {2.72315, 13.7956}},
 Eccentricity → {All → {-0.195306, 0.807774}, 1 → {-0.0486485, 0.777741},
   2 → {-0.481118, 0.779543}, 3 → {-0.0779731, 0.793915}}}
```

Here, we do errors over the range 8.75° - 18.75°.

```
data20VoronoiErrorsOuter = AggregateErrors[
   data20,
   data10[VoronoiPrediction, OurSchiraModel],
   {8.75, 18.75}];
ReportAggregateErrors[data20VoronoiErrorsOuter]
```

```
{PolarAngle → {All → {2.54755, 16.2539},
   1 → {-5.90181, 9.60608}, 2 → {16.636, 18.2211}, 3 → {3.00479, 18.0869}},
 Eccentricity → {All → {1.305, 2.36234}, 1 → {1.4863, 2.7315},
   2 → {0.133312, 1.67663}, 3 → {2.20901, 2.80215}}}
```

And here we do errors over the range 1.25° - 8.75°.

```
data20VoronoiErrorsInner = AggregateErrors[
   data20,
   data10[VoronoiPrediction, OurSchiraModel],
   {1.25, 8.75}];
ReportAggregateErrors[data20VoronoiErrorsInner]
```

```
{PolarAngle → {All → {5.47721, 11.857},
   1 → {-1.6025, 8.27569}, 2 → {11.2712, 12.4909}, 3 → {2.6652, 12.5473}},
 Eccentricity → {All → {-0.327912, 0.576148}, 1 → {-0.220437, 0.470704},
   2 → {-0.515554, 0.625397}, 3 → {-0.335332, 0.594661}}}
```

5.3. Test/Retest Analysis

This makes the same report as above in the leave-one-out errors section, but it uses test/retest data instead of leave-one-out predictions, so what we get here is an estimate of the error between two identical 20 minute scans, in a comparable report to the leave-one-out errors.

```
With[
 {sim = sim10,
  data = dataRetest},
 With[
  {fMin = 3,
   ecMin = 1.25, ecMax = 8.75},
  With[
   {err = Reap[
       Map[
        With[
          {lh1 = #[[1, 1]],
           lh2 = #[[2, 1]],
           rh1 = #[[1, 2]],
           rh2 = #[[2, 2]]},
          MapThread[
           If[#1[[5]] > fMin && #2[[5]] > fMin && 0 < Abs[#3[[5]]] < 4 && #1[[3]] ≠ 0 &&
               #2[[3]] ≠ 0 && ecMin ≤ #1[[4]] ≤ ecMax && ecMin ≤ #2[[4]] ≤ ecMax,
             Sow[{{#1[[3]], #2[[3]]}, {#1[[4]], #2[[4]]}}, {0, Round[#3[[5]]]}]
            ] &,
           {Join[lh1, rh1],
            Join[lh2, rh2],
            With[{tmp = MatchOrder[sim[SmoothPredictionFull] → lh1]},
             Join[tmp, tmp]]}]
         ] &,
        Partition[data[Data], 2]],
       {0, 1, 2, 3},
       Apply[Sequence, #2] &
      ][[2]]},
   MapThread[
    Rule[
      #1,
      Map[
       {Median@#, Median@Abs@#} &,
       List[
        MapThread[Subtract, Transpose[#2[[All, 1]]]],
        MapThread[Subtract, Transpose[#2[[All, 2]]]]]]
     ] &,
    {{All, 1, 2, 3}, err}]]]]
```

```
{All → {{0.176834, 7.49586}, {0.00671768, 0.5}},
 1 → {{-1.39719, 13.7767}, {-0.101168, 0.686035}},
 2 → {{0.320091, 7.12503}, {0.00734663, 0.496135}},
 3 → {{0.581678, 9.85724}, {0.0394301, 0.633874}}}
```

5.4. Accuracy of the Aggregate

Here we calculate the LOO prediction error when the prediction for the left-out subject is just the aggregate of the remaining subjects.

```
ReportLOOErrors[
 data10,
 Map[
  sub Ì MapThread[
    {row, area} Ì ReplacePart[row, 5 → area],
    {data10[Aggregate, LOO[sub]], sim10[SmoothPredictionFull][[All, 5]]}],
  data10[Subjects]],
 {1.25, 8.75}]
```

Area	ϵ_ρ	$\lvert\epsilon_\rho\rvert$	ϵ_θ	$\lvert\epsilon_\theta\rvert$
V1	0.288204	0.614351	−7.81213	21.2368
V2	0.042629	0.957058	−1.09091	16.1487
V3	0.159682	1.09045	−3.18332	20.5131
All	0.0529652	0.961309	−1.29239	16.4721

5.5. Accuracy without Registration

```
With[
 {mdl = InverseSchiraFunction[OurSchiraModel],
  data = data10},
 With[
  {fMin = 0,
   ecMin = 1.25, ecMax = 8.75,
   full = Quiet[
     Map[
      mdl[#[[1]], #[[2]]] &,
      LocalAnatomy]]},
  With[
   {err = Reap[
       Scan[
        dat Ì MapThread[
          {row, pred} Ì If[
            ListQ[pred] && row[[5]] > fMin && ecMin ≤ row[[4]] ≤ ecMax,
            Sow[{{row[[3]], pred[[1]]},
              {row[[4]], pred[[2]]}}, {0, Round[pred[[3]]]}]],
          {dat, full}],
        data[Data],
        {2}],
       {0, 1, 2, 3},
       Apply[Sequence, #2] &
      ][[2]]},
   MapThread[
    Rule[
      #1,
      Map[
       {Median@#, Median@Abs@#} &,
       List[
        MapThread[Subtract, Transpose[#2[[All, 1]]]],
        MapThread[Subtract, Transpose[#2[[All, 2]]]]]]
     ] &,
    {{All, 1, 2, 3}, err}]]]]
```

```
{All → {{-5.09435, 37.2782}, {-5.79545, 5.94235}},
 1 → {{-15.0364, 33.1957}, {-6.13449, 6.19674}},
 2 → {{1.61995, 37.5238}, {-10.3796, 10.3796}},
 3 → {{-7.46367, 37.3402}, {-2.70076, 3.07255}}}
```

6. Figures

6.1. Anatomy Tinted by Region

These figures show the sulcal curvature of the local region around the occipital pole with those regions found to be in V1, V2, and V3 tinted red, green, or blue respectively.

6.1.1. FSAverage-sym Space

This image is the data in FSAverage-sym space (ie, prior to simulation).

```
figRegionsFSAverage = With[
   {dat = Select[sim10[SmoothPrediction][[All, {1, 2, 5}]], 0 < #[[3]] < 4 &],
    bgColorFn = (If[# < -0.03051, LightGray, Gray] &),
    precision = 0.0000001},
   CorticalPlot[
    Reap[
      Scan[
       If[Length@# == 2, Sow[{#[[1, 1]], #[[1, 2]], #[[1, 3]] + #[[2, 3]]}]] &,
       Split[
        Sort[
         Join[
          {#[[1]], #[[2]], 10 * #[[3]]} & /@ dat,
          LocalAnatomy],
         Which[
            #1[[1]] - #2[[1]] > precision, True,
            #1[[1]] - #2[[1]] < -precision, False,
            #1[[2]] - #2[[2]] > precision, True,
            #1[[2]] - #2[[2]] < -precision, False,
            #1[[3]] > 9, True,
            True, False
          ] &],
        (Norm[#1[[1 ;; 2]] - #2[[1 ;; 2]]] < precision) &]]
     ][[2, 1]],
    CortexOutline → $Data10FSAverageOutline,
    ColorFunction → (Which[
        # > 25, Blend[{Blue, bgColorFn[# - 30]}, 0.75],
        # > 15, Blend[{Green, bgColorFn[# - 20]}, 0.75],
        # > 5, Blend[{Red, bgColorFn[# - 10]}, 0.75],
        True, Die["Bad value: " <> ToString[#]]
      ] &),
    ImageSize → 1.15 * 72]];
```

6.1.2. Spring Space

This shows the anatomy as changed by simulation (ie, in the corrected topology).

```
figRegionsCorrected = With[{precision = 0.0000001},
   With[
    {dat = Select[
        Map[
         If[Length@# == 2,
           {#[[2, 3]], #[[2, 4]], #[[1, 3]]},
           {#[[1, 1]], #[[1, 2]], 0}
          ] &,
         Split[
          Sort[
           Join[
            sim10[SmoothPrediction][[All, {1, 2, 5}]],
            MapThread[Join[#1[[1 ;; 2]], #2[[1 ;; 2]]] &,
             {data10[Aggregate], sim10[Result]}]],
```

```
          Which[
            #1[[1]] - #2[[1]] > precision, True,
            #1[[1]] - #2[[1]] < -precision, False,
            #1[[2]] - #2[[2]] > precision, True,
            #1[[2]] - #2[[2]] < -precision, False,
            Length[#1] == 3, True,
            True, False
           ] &],
        (Norm[#1[[1 ;; 2]] - #2[[1 ;; 2]]] < precision) &]],
      0 < #[[3]] < 4 &],
    bgColorFn = (If[# < -0.03051, LightGray, Gray] &)},
   Show[
    CorticalPlot[
     Reap[
       Scan[
        If[Length@# == 2, Sow[{#[[1, 1]], #[[1, 2]], #[[1, 3]] + #[[2, 3]]}]] &,
        Split[
         Sort[
          Join[
            {#[[1]], #[[2]], 10 * #[[3]]} & /@ dat,
            DistortData[LocalAnatomy, sim10[Result]]],
          Which[
             #1[[1]] - #2[[1]] > precision, True,
             #1[[1]] - #2[[1]] < -precision, False,
             #1[[2]] - #2[[2]] > precision, True,
             #1[[2]] - #2[[2]] < -precision, False,
             #1[[3]] > 9, True,
             True, False
            ] &],
         (Norm[#1[[1 ;; 2]] - #2[[1 ;; 2]]] < precision) &]]
      ][[2, 1]],
     CortexOutline -> $Data10CorrectedOutline,
     ColorFunction -> (Which[
          # > 25, Blend[{Blue, bgColorFn[# - 30]}, 0.75],
          # > 15, Blend[{Green, bgColorFn[# - 20]}, 0.75],
          # > 5, Blend[{Red, bgColorFn[# - 10]}, 0.75],
          True, Die["Bad value: " <> ToString[#]]
        ] &),
     V1Outline -> None,
     AnatomyData -> DistortData[LocalAnatomy, sim10[Result]],
     ImageSize -> 1.15 * 72],
    SchiraLinePlotHash[sim10[SchiraModel], PolarAngle]]]];
```

6.1.3. Together

The previous two images combined into a single explanatory graphic.

```
figRegionsCombined = With[
  {img = Graphics[
     {Inset[
       figRegionsFSAverage,
       ImageScaled[{0, 0.5}],
       ImageScaled[{0, 0.5}]],
      Inset[
       figRegionsCorrected,
       ImageScaled[{1, 0.5}],
       ImageScaled[{1, 0.5}]],
      Inset[
       Style["V1", FontSize → 10,
        FontFamily → "Arial", FontColor → Red, FontWeight → Bold],
       ImageScaled[{0.5, 0.5}],
       ImageScaled[{0.5, 0.5}]],
      Inset[
       Style["V2", FontSize → 10, FontFamily → "Arial",
        FontColor → Darker[Green], FontWeight → Bold],
       ImageScaled[{0.5, 0.65}],
       ImageScaled[{0.5, 0.5}]],
      Inset[
       Style["V3", FontSize → 10,
        FontFamily → "Arial", FontColor → Blue, FontWeight → Bold],
       ImageScaled[{0.5, 0.8}],
       ImageScaled[{0.5, 0.5}]]},
     ImagePadding → 2000,
     ImageSize → {3.4, 1.2} * 72]},
  img];
Image[figRegionsCombined, ImageResolution → 150]
```

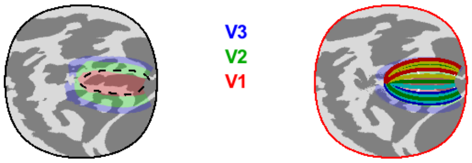

6.2. Aggregates of the 10° Dataset in the Various Topologies

This shows the aggregate of the dataset in the FSaverage-sym space as well as in the corrected topology.

6.2.1. Aggregate Figure in FSAverage-sym Topology

These are just the raw aggregate maps of the retinotopic data for the 19 subjects in the 10° dataset.

```
{figData10AggEcc, figData10AggPol, eccLegend, polLegend, figData10Agg} = With[
   {paImg = CorticalPlot[
      SelectPolarAngle[data10[Aggregate], 22, {0, 10}],
      CortexOutline → $Data10FSAverageOutline,
      ColorFunction → $PolarAngleColorFn,
      ImageSize → 1.15 * 72],
    ecImg = CorticalPlot[
      SelectEccentricity[data10[Aggregate], 22, {0, 10}],
      CortexOutline → $Data10FSAverageOutline,
      ColorFunction → $EccentricityColorFn,
      ImageSize → 1.15 * 72],
    paLeg = PolarAngleLegend[Right, ImageSize → 0.45 * 72],
    ecLeg = EccentricityLegend[Left, ImageSize → 0.45 * 72]},
   {ecImg, paImg, ecLeg, paLeg,
    Graphics[
     {Inset[ecLeg, ImageScaled[{0.495, 0}], ImageScaled[{1, 0}]],
      Inset[paLeg, ImageScaled[{0.505, 0}], ImageScaled[{0, 0}]],
      Inset[ecImg, ImageScaled[{0, 1}], ImageScaled[{0, 1}]],
      Inset[paImg, ImageScaled[{1, 1}], ImageScaled[{1, 1}]]},
     ImageSize → {3.4, 1.15} * 72,
     ImagePadding → 2000]}];
Image[figData10Agg, ImageResolution → 150]
```

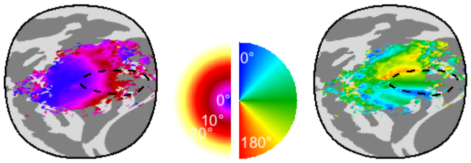

Here, we show the same figures, but with the Schira model superimposed in order to demonstrate how the aggregate data deviates from the model itself.

6.2.2. Aggregate Figures in the Spring Corrected Topology

This figure shows the same data as the previous figure, but it is distorted into the corrected topology and shown with the schira model imposed.

```
{figData10SSEcc, figData10SSPol, eccLegend, polLegend, figData10SS} = With[
   {sim = sim10[Result],
    data = data10,
    agg = data10[Aggregate]},
   With[
    {paImg = Show[
       CorticalPlot[
        SelectPolarAngle[
         DistortData[agg, sim],
         data[FStatRange][[1]],
         {0, 10}],
        CortexOutline → $Data10CorrectedOutline,
        ColorFunction → $PolarAngleColorFn,
        V1Outline → None,
        ImageSize → 1.15 * 72,
        AnatomyData → DistortData[LocalAnatomy, sim]],
       SchiraLinePlot[
        sim10[SchiraModel],
        PolarAngleStyleFunction →
         ({Thickness[0.006], Darker[$PolarAngleColorFn[#]]} &),
        EccentricityStyleFunction → ({Thickness[0.004], Dashed, White} &),
        EccentricityLines → {10, 90},
        Axes → None,
        Frame → False]],
     ecImg = Show[
       CorticalPlot[
        SelectEccentricity[
         DistortData[agg, sim],
         data[FStatRange][[1]],
         {0, 10}],
        CortexOutline → $Data10CorrectedOutline,
        ColorFunction → $EccentricityColorFn,
        V1Outline → None,
        ImageSize → 1.15 * 72,
        AnatomyData → DistortData[LocalAnatomy, sim]],
       SchiraLinePlot[
        sim10[SchiraModel],
        EccentricityStyleFunction →
         ({Thickness[0.006], Darker[$EccentricityColorFn[#]]} &),
        PolarAngleStyleFunction → ({Thickness[0.004], White} &),
        PolarAngleLines → {{0, 180}, None, {0, 180}},
        Axes → None,
        Frame → False]],
     paLeg = PolarAngleLegend[Right, ImageSize → 0.45 * 72],
     ecLeg = EccentricityLegend[Left, ImageSize → 0.45 * 72]},
    {ecImg, paImg, ecLeg, paLeg,
     Graphics[
      {Inset[ecLeg, ImageScaled[{0.495, 0}], ImageScaled[{1, 0}]],
       Inset[paLeg, ImageScaled[{0.505, 0}], ImageScaled[{0, 0}]],
       Inset[ecImg, ImageScaled[{0, 1}], ImageScaled[{0, 1}]],
       Inset[paImg, ImageScaled[{1, 1}], ImageScaled[{1, 1}]]},
      ImageSize → {3.4, 1.25} * 72,
      ImagePadding → 2000]}]];
Image[figData10SS, ImageResolution → 150]
```

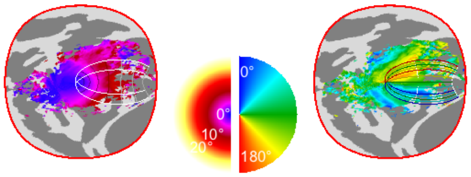

6.2.3. Aggregate Figures in the Delaunay Corrected Topology

This figure shows the same data as the previous figure, but it is distorted into the corrected topology and shown with the schira model imposed.

```
{figData10VoronoiEcc, figData10VoronoiPol,
   eccLegend, polLegend, figData10Voronoi} = With[
   {sim = data10[VoronoiRegister, OurSchiraModel],
    data = data10,
    agg = data10[Aggregate]},
   With[
    {paImg = Show[
       CorticalPlot[
        SelectPolarAngle[
         DistortData[agg, sim],
         0.001,
         {0, 10}],
        CortexOutline → $Data10CorrectedOutline,
        ColorFunction → $PolarAngleColorFn,
        V1Outline → None,
        ImageSize → 1.15 * 72,
        AnatomyData → DistortData[LocalAnatomy, sim]],
       SchiraLinePlot[
        sim10[SchiraModel],
        PolarAngleStyleFunction →
          ({Thickness[0.006], Darker[$PolarAngleColorFn[#]]} &),
        EccentricityStyleFunction → ({Thickness[0.004], Dashed, White} &),
        EccentricityLines → {10, 90},
        Axes → None,
        Frame → False]],
     ecImg = Show[
       CorticalPlot[
        SelectEccentricity[
         DistortData[agg, sim],
         0.001,
         {0, 10}],
        CortexOutline → $Data10CorrectedOutline,
        ColorFunction → $EccentricityColorFn,
        V1Outline → None,
        ImageSize → 1.15 * 72,
        AnatomyData → DistortData[LocalAnatomy, sim]],
       SchiraLinePlot[
        sim10[SchiraModel],
        EccentricityStyleFunction →
          ({Thickness[0.006], Darker[$EccentricityColorFn[#]]} &),
        PolarAngleStyleFunction → ({Thickness[0.004], White} &),
        PolarAngleLines → {{0, 180}, None, {0, 180}},
        Axes → None,
```

```
        Frame → False]],
     paLeg = PolarAngleLegend[Right, ImageSize → 0.45 * 72],
     ecLeg = EccentricityLegend[Left, ImageSize → 0.45 * 72]},
    {ecImg, paImg, ecLeg, paLeg,
     Graphics[
      {Inset[ecLeg, ImageScaled[{0.495, 0}], ImageScaled[{1, 0}]],
       Inset[paLeg, ImageScaled[{0.505, 0}], ImageScaled[{0, 0}]],
       Inset[ecImg, ImageScaled[{0, 1}], ImageScaled[{0, 1}]],
       Inset[paImg, ImageScaled[{1, 1}], ImageScaled[{1, 1}]]},
      ImageSize → {3.4, 1.25} * 72,
      ImagePadding → 2000]}]];
Image[figData10Voronoi, ImageResolution → 150]
```

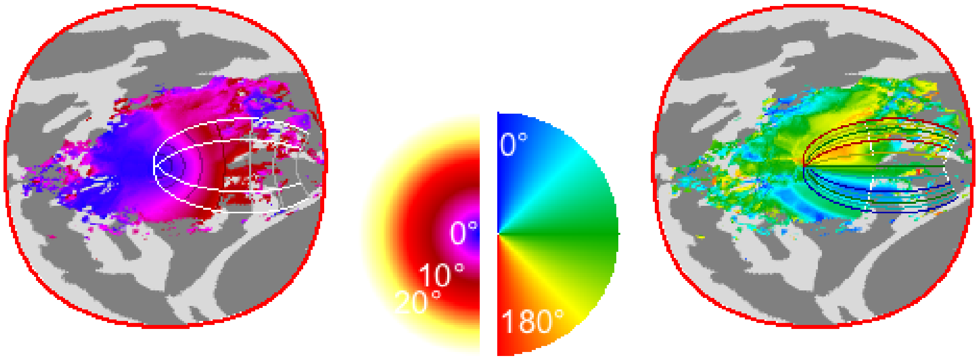

6.3. Distortion Field Plots

6.3.1. Spring Simulations

This figure shows the amount (distance) and direction of distortion caused by the simulation.

```
{figSSNormDist, figSSDirDist, figSSDistortion} = With[
   {simres = sim10[Result]},
   With[
    {imgNorm = With[
       {dat = MapThread[
          Append[#1[[1 ;; 2]], Norm[#1[[1 ;; 2]] - #2[[1 ;; 2]]]] &,
          {LocalAnatomy, simres}],
        xyrng = {
          {Min@LocalAnatomy[[All, 1]], Max@LocalAnatomy[[All, 1]]},
          {Min@LocalAnatomy[[All, 2]], Max@LocalAnatomy[[All, 2]]}}},
       With[{mx = Max[dat[[All, 3]]]},
        Graphics[
         {Inset[
           Show[
            {ListDensityPlot[
              dat,
              ColorFunction → (If[# > 0.2, Black, Blend[{White, Black}, # / 0.2]] &),
              ColorFunctionScaling → False,
              PlotRange → Full,
              ImageSize → 1.15 * 72,
              AspectRatio → 1,
```

```
          Frame → False,
          Axes → False,
          Epilog → {
            Thick, Black,
            Line[
             {{xyrng[[1, 1]], xyrng[[2, 1]] * 0.95 + xyrng[[2, 2]] * 0.05},
              {xyrng[[1, 1]] + 0.2,
               xyrng[[2, 1]] * 0.95 + xyrng[[2, 2]] * 0.05}}],
            Thickness[Medium], Black, Line[LocalHull]}]},
        V1HullPlot],
       ImageScaled[{0, 1}],
       ImageScaled[{0, 1}]],
      Inset[
       Style["0.2", FontFamily → "Arial", FontSize → 10],
       ImageScaled[{0, 0}],
       ImageScaled[{0, 0}]],
      Inset[
       ArrayPlot[
        Reverse[Table[{k, k}, {k, 0, 0.2, 0.2 / 100}]],
        DataRange → {{0, 1}, {0, 0.2}},
        ColorFunction → (Blend[{White, Black}, #] &),
        Frame → {{False, True}, {False, False}},
        FrameTicks →
         {{False, Range[0.0, 0.2, 0.1] /. (0. → {0., "0.0"})}, {False, False}},
        BaseStyle → Directive[10, FontFamily → "Arial"],
        ImageSize → 0.35 * 72,
        AspectRatio → 3],
       ImageScaled[{1, 0}],
       ImageScaled[{1, 0}]]},
     ImagePadding → 10 000,
     ImageSize → {1.5, 1.25} * 72]]],
imgAng = With[
  {dat = MapThread[
     Append[
       #1[[1 ;; 2]],
       If[Norm[#1[[1 ;; 2]] - #2[[1 ;; 2]]] < 0.01,
        - (ArcTan[#2[[1]] - #1[[1]], #2[[2]] - #1[[2]]] + Pi) / (2 * Pi),
        (ArcTan[#2[[1]] - #1[[1]], #2[[2]] - #1[[2]]] + Pi) / (2 * Pi)]] &,
     {LocalAnatomy, simres}]},
  With[{mx = Max[dat[[All, 3]]]},
   Graphics[
    {Inset[
      Show[
       {ListDensityPlot[
         dat,
         ColorFunction → (If[# < 0, Hue[#, 0.3, 1], Hue[#]] &),
         ColorFunctionScaling → False,
         PlotRange → Full,
         ImageSize → 1.15 * 72,
         AspectRatio → 1,
         Frame → False,
         Axes → False,
         Epilog → {Thickness[Medium], Black, Line[LocalHull]}],
        V1HullPlot}],
      ImageScaled[{0, 1}],
```

```
                 ImageScaled[{0, 1}]],
                Inset[
                 ListDensityPlot[
                  Flatten[
                   Table[
                    {r * Cos[th], r * Sin[th], If[
                      r < 0.25, -(th + Pi) / (2 * Pi), (th + Pi) / (2 * Pi)]},
                    {r, 0.05, 1, 0.05},
                    {th, -Pi, Pi - 0.01, 0.05}],
                   1],
                  ColorFunction → (If[# < 0, Hue[#, 0.3, 1], Hue[#]] &),
                  ColorFunctionScaling → False,
                  ImageSize → 0.25 * 72,
                  AspectRatio → 1,
                  Frame → False,
                  Axes → False],
                 ImageScaled[{1, 0}],
                 ImageScaled[{1, 0}]]},
               ImageSize → {1.25, 1.25} * 72]]]},
      {imgNorm, imgAng,
       Graphics[
        {Inset[imgNorm, ImageScaled[{0, 0}], ImageScaled[{0, 0}]],
         Inset[imgAng, ImageScaled[{1, 0}], ImageScaled[{1, 0}]]},
        ImageSize → {3.4, 1.25} * 72,
        ImagePadding → 2000]}]];
Image[figSSDistortion, ImageResolution → 150]
```

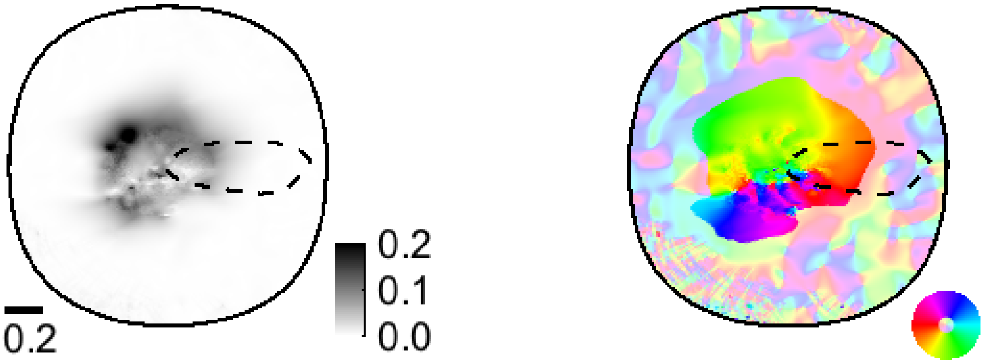

6.3.2. Voronoi Mesh

This figure shows the amount (distance) and direction of distortion caused by the simulation.

```
{figVoronoiNormDist, figVoronoiDirDist, figVoronoiDistortion} = With[
   {simres = data10[VoronoiRegister, SchiraModel[]]},
   With[
    {imgNorm = With[
       {dat = MapThread[
          Append[#1[[1 ;; 2]], Norm[#1[[1 ;; 2]] - #2[[1 ;; 2]]]] &,
          {LocalAnatomy, simres}],
        xyrng = {
          {Min@LocalAnatomy[[All, 1]], Max@LocalAnatomy[[All, 1]]},
          {Min@LocalAnatomy[[All, 2]], Max@LocalAnatomy[[All, 2]]}}},
```

```
    With[{mx = Max[dat[[All, 3]]]},
     Graphics[
      {Inset[
        Show[
         {ListDensityPlot[
           dat,
           ColorFunction → (If[# > 0.2, Black, Blend[{White, Black}, # / 0.2]] &),
           ColorFunctionScaling → False,
           PlotRange → Full,
           ImageSize → 1.15 * 72,
           AspectRatio → 1,
           Frame → False,
           Axes → False,
           Epilog → {
             Thick, Black,
             Line[
              {{xyrng[[1, 1]], xyrng[[2, 1]] * 0.95 + xyrng[[2, 2]] * 0.05},
               {xyrng[[1, 1]] + 0.2,
                xyrng[[2, 1]] * 0.95 + xyrng[[2, 2]] * 0.05}}],
             Thickness[Medium], Black, Line[LocalHull]}]},
         V1HullPlot],
        ImageScaled[{0, 1}],
        ImageScaled[{0, 1}]],
       Inset[
        Style["0.2", FontFamily → "Arial", FontSize → 10],
        ImageScaled[{0, 0}],
        ImageScaled[{0, 0}]],
       Inset[
        ArrayPlot[
         Reverse[Table[{k, k}, {k, 0, 0.2, 0.2 / 100}]],
         DataRange → {{0, 1}, {0, 0.2}},
         ColorFunction → (Blend[{White, Black}, #] &),
         Frame → {{False, True}, {False, False}},
         FrameTicks →
          {{False, Range[0.0, 0.2, 0.1] /. (0. → {0., "0.0"})}, {False, False}},
         BaseStyle → Directive[10, FontFamily → "Arial"],
         ImageSize → 0.35 * 72,
         AspectRatio → 3],
        ImageScaled[{1, 0}],
        ImageScaled[{1, 0}]]},
      ImagePadding → 10 000,
      ImageSize → {1.5, 1.25} * 72]]],
  imgAng = With[
    {dat = MapThread[
       Append[
         #1[[1 ;; 2]],
         Which[
          #1[[1 ;; 2]] == #2[[1 ;; 2]], 0,
          Norm[#1[[1 ;; 2]] - #2[[1 ;; 2]]] < 0.01,
          - (ArcTan[#2[[1]] - #1[[1]], #2[[2]] - #1[[2]]] + Pi) / (2 * Pi),
          True,
          (ArcTan[#2[[1]] - #1[[1]], #2[[2]] - #1[[2]]] + Pi) / (2 * Pi)]] &,
       {LocalAnatomy, simres}]},
    With[{mx = Max[dat[[All, 3]]]},
     Graphics[
```

```
            {Inset[
              Show[
               {ListDensityPlot[
                 dat,
                 ColorFunction → (If[# < 0, Hue[#, 0.3, 1], Hue[#]] &),
                 ColorFunctionScaling → False,
                 PlotRange → Full,
                 ImageSize → 1.15 * 72,
                 AspectRatio → 1,
                 Frame → False,
                 Axes → False,
                 Epilog → {Thickness[Medium], Black, Line[LocalHull]}],
                V1HullPlot}],
              ImageScaled[{0, 1}],
              ImageScaled[{0, 1}]],
             Inset[
              ListDensityPlot[
               Flatten[
                Table[
                 {r * Cos[th], r * Sin[th], If[
                   r < 0.25, -(th + Pi) / (2 * Pi), (th + Pi) / (2 * Pi)]},
                 {r, 0.05, 1, 0.05},
                 {th, -Pi, Pi - 0.01, 0.05}],
                1],
               ColorFunction → (If[# < 0, Hue[#, 0.3, 1], Hue[#]] &),
               ColorFunctionScaling → False,
               ImageSize → 0.25 * 72,
               AspectRatio → 1,
               Frame → False,
               Axes → False],
              ImageScaled[{1, 0}],
              ImageScaled[{1, 0}]]},
            ImageSize → {1.25, 1.25} * 72]]]},
     {imgNorm, imgAng,
      Graphics[
       {Inset[imgNorm, ImageScaled[{0, 0}], ImageScaled[{0, 0}]],
        Inset[imgAng, ImageScaled[{1, 0}], ImageScaled[{1, 0}]]},
       ImageSize → {3.4, 1.25} * 72,
       ImagePadding → 2000]}]];
Image[figVoronoiDistortion, ImageResolution → 150]
```

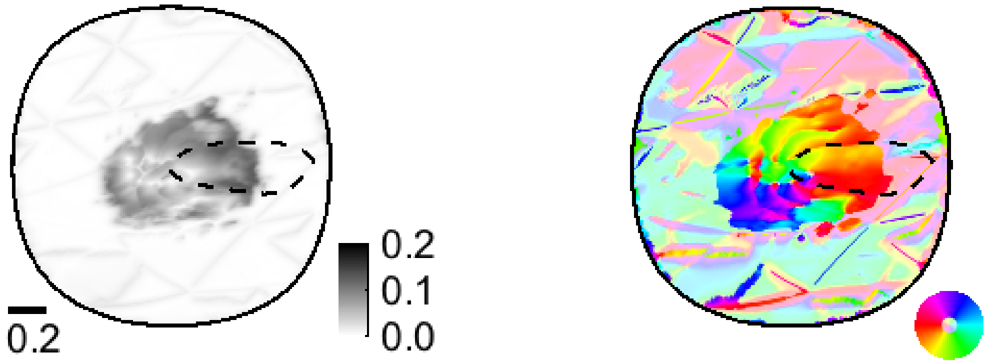

6.3.3. Schira Model

This image shows the distortion projected onto the Schira model. To be precise, this takes the polar angle and eccentricity that is observed at a particular vertex, and plots the distortion of that vertex at the position in the Schira model that it predicts for the observed polar angle and eccentricity.

```
With[
 {simpre = sim10[SmoothPredictionFull],
  simres = sim10[Result],
  agg = data10[Aggregate],
  sfn = SchiraFunction[OurSchiraModel]},
 With[
  {imgNorm = With[
     {dat = Select[
        MapThread[
         If[0 < Abs[#2[[5]]] < 4,
           Append[
            {Re@#, Im@#} &@sfn[#1[[3]], #1[[4]], #2[[5]]],
            Norm[#1[[1 ;; 2]] - #3[[1 ;; 2]]]],
           None
          ] &,
         {agg, simpre, simres}],
        ListQ],
      xyrng = {
        {Min@simres[[All, 1]], Max@simres[[All, 1]]},
        {Min@simres[[All, 2]], Max@simres[[All, 2]]}}},
     With[{mx = Max[dat[[All, 3]]]},
      Graphics[
       {Inset[
         Show[
          {ListDensityPlot[
            dat,
            ColorFunction → (If[# > 0.2, Black, Blend[{White, Black}, # / 0.2]] &),
            ColorFunctionScaling → False,
            PlotRange → Full,
            ImageSize → 1.15 * 72,
            AspectRatio → 1,
            Frame → False,
            Axes → False,
            Epilog → {
              Thick, Black,
              Line[
               {{xyrng[[1, 1]], xyrng[[2, 1]] * 0.95 + xyrng[[2, 2]] * 0.05},
                {xyrng[[1, 1]] + 0.2,
                 xyrng[[2, 1]] * 0.95 + xyrng[[2, 2]] * 0.05}}]}],
           SchiraLinePlotHash[OurSchiraModel, PolarAngle]}],
         ImageScaled[{0, 1}],
         ImageScaled[{0, 1}]],
        Inset[
         Style["0.2", FontFamily → "Arial", FontSize → 10],
         ImageScaled[{0, 0}],
         ImageScaled[{0, 0}]],
        Inset[
         ArrayPlot[
          Reverse[Table[{k, k}, {k, 0, 0.2, 0.2 / 100}]],
```

```
          DataRange → {{0, 1}, {0, 0.2}},
          ColorFunction → (Blend[{White, Black}, #] &),
          Frame → {{False, True}, {False, False}},
          FrameTicks →
           {{False, Range[0.0, 0.2, 0.1] /. (0. → {0., "0.0"})}, {False, False}},
          BaseStyle → Directive[10, FontFamily → "Arial"],
          ImageSize → 0.35 * 72,
          AspectRatio → 3],
         ImageScaled[{1, 0}],
         ImageScaled[{1, 0}]]},
       ImagePadding → 10 000,
       ImageSize → {1.5, 1.25} * 72]]],
   imgAng = With[
     {dat = Select[
        MapThread[
         If[0 < Abs[#2[[5]]] < 4,
           Append[
            {Re@#, Im@#} &@sfn[#1[[3]], #1[[4]], #2[[5]]],
            Which[
             #1[[1 ;; 2]] == #3[[1 ;; 2]], 0,
             Norm[#1[[1 ;; 2]] - #3[[1 ;; 2]]] < 0.01,
             -(ArcTan[#1[[1]] - #3[[1]], #1[[2]] - #3[[2]]] + Pi) / (2 * Pi),
             True, (ArcTan[#1[[1]] - #3[[1]], #1[[2]] - #3[[2]]] + Pi) / (2 * Pi)]],
           None
          ] &,
         {agg, simpre, simres}],
        ListQ]},
     With[{mx = Max[dat[[All, 3]]]},
      Graphics[
       {Inset[
         Show[
          {ListDensityPlot[
            dat,
            ColorFunction → (If[# < 0, Hue[#, 0.3, 1], Hue[#]] &),
            ColorFunctionScaling → False,
            PlotRange → Full,
            ImageSize → 1.15 * 72,
            AspectRatio → 1,
            Frame → False,
            Axes → False],
           SchiraLinePlotHash[OurSchiraModel, PolarAngle]}],
         ImageScaled[{0, 1}],
         ImageScaled[{0, 1}]],
        Inset[
         ListDensityPlot[
          Flatten[
           Table[
            {r * Cos[th], r * Sin[th],
             If[r < 0.25, -(th + Pi) / (2 * Pi), (th + Pi) / (2 * Pi)]},
            {r, 0.05, 1, 0.05},
            {th, -Pi, Pi - 0.01, 0.05}],
           1],
          ColorFunction → (If[# < 0, Hue[#, 0.3, 1], Hue[#]] &),
          ColorFunctionScaling → False,
          ImageSize → 0.25 * 72,
```

```
AspectRatio → 1,
Frame → False,
Axes → False],
ImageScaled[{1, 0}],
ImageScaled[{1, 0}]]},
ImageSize → {1.25, 1.25} * 72]]]},
{imgNorm, imgAng}]]
```

{ 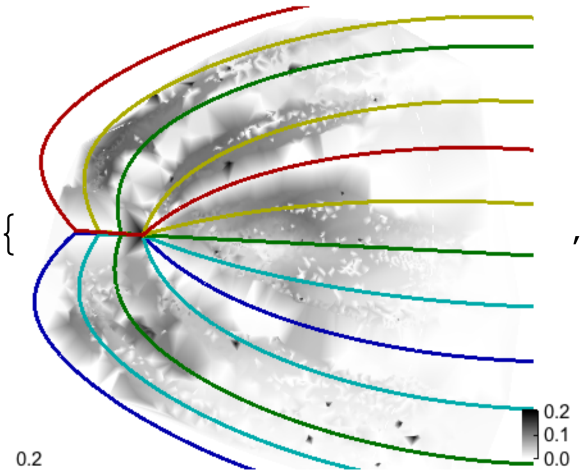

, 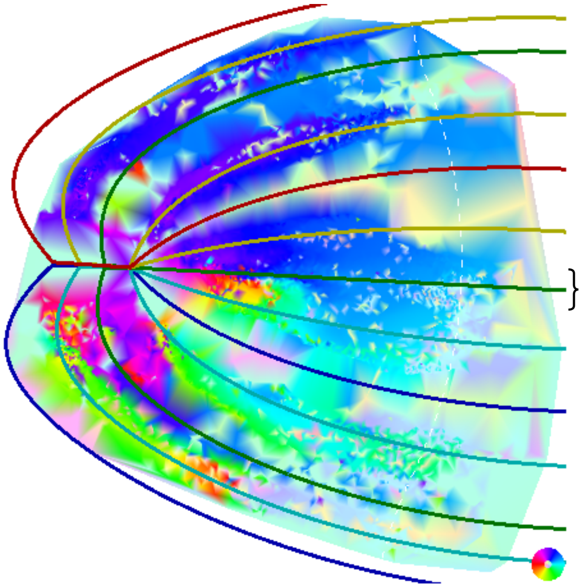 }

6.4. Aggregates of the 20° Dataset

6.4.1. Aggregate in the FSAverage-sym Topology

```
{figData20AggEcc, figData20AggPol, eccLegend, polLegend, figData20Agg} = With[
   {paImg = CorticalPlot[
      SelectPolarAngle[data20[Aggregate], 18, {0, 20}],
      CortexOutline → $Data10FSAverageOutline,
      ColorFunction → $PolarAngleColorFn,
      ImageSize → 1.15 * 72],
    ecImg = CorticalPlot[
      SelectEccentricity[data20[Aggregate], 18, {0, 20}],
      CortexOutline → $Data10FSAverageOutline,
      ColorFunction → $EccentricityColorFn,
      ImageSize → 1.15 * 72],
    paLeg = PolarAngleLegend[Right, ImageSize → 0.45 * 72],
    ecLeg = EccentricityLegend[Left, ImageSize → 0.45 * 72]},
   {ecImg, paImg, ecLeg, paLeg,
    Graphics[
     {Inset[ecLeg, ImageScaled[{0.495, 0}], ImageScaled[{1, 0}]],
      Inset[paLeg, ImageScaled[{0.505, 0}], ImageScaled[{0, 0}]],
      Inset[ecImg, ImageScaled[{0, 1}], ImageScaled[{0, 1}]],
      Inset[paImg, ImageScaled[{1, 1}], ImageScaled[{1, 1}]]},
     ImageSize → {3.4, 1.15} * 72,
     ImagePadding → 2000]}];
Image[figData20Agg, ImageResolution → 150]
```

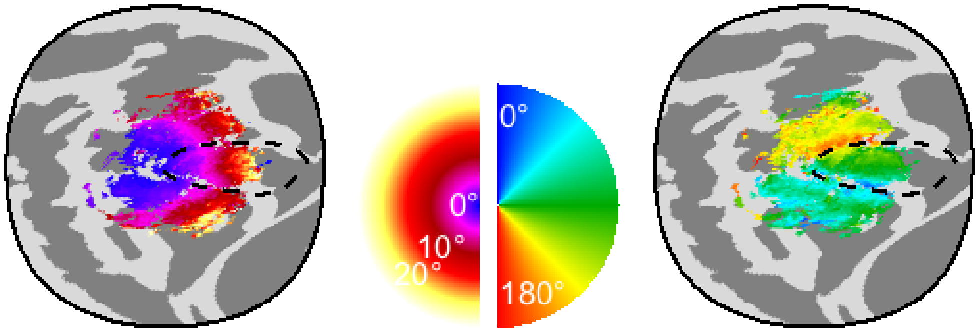

6.4.2. Aggregate in Spring-Corrected Topology

```
{figData20SSEcc, figData20SSPol, eccLegend, polLegend, figData20SS} = With[
   {simres = sim10[Result],
    agg = data20[Aggregate],
    schira = sim10[SchiraModel]},
   With[
    {paImg = Show[
       CorticalPlot[
        SelectPolarAngle[DistortData[agg, simres], 18, {0, 20}],
        CortexOutline → $Data20CorrectedOutline,
        ColorFunction → $PolarAngleColorFn,
        V1Outline → None,
        AnatomyData → DistortData[LocalAnatomy, simres],
        ImageSize → 1.15 * 72],
       SchiraLinePlot[
        schira,
        PolarAngleStyleFunction →
         ({Thickness[0.006], Darker[$PolarAngleColorFn[#]]} &),
        EccentricityStyleFunction → ({Thickness[0.004], Dashed, White} &),
        EccentricityLines → {10, 90},
        Axes → None,
        Frame → False]],
     ecImg = Show[
       CorticalPlot[
        SelectEccentricity[DistortData[agg, simres], 18, {0, 20}],
        CortexOutline → $Data20CorrectedOutline,
        ColorFunction → $EccentricityColorFn,
        V1Outline → None,
        AnatomyData → DistortData[LocalAnatomy, simres],
        ImageSize → 1.15 * 72],
       SchiraLinePlot[
        schira,
        EccentricityStyleFunction →
         ({Thickness[0.006], Darker[$EccentricityColorFn[#]]} &),
        PolarAngleStyleFunction → ({Thickness[0.004], White} &),
        PolarAngleLines → {{0, 180}, None, {0, 180}},
        Axes → None,
        Frame → False]],
     paLeg = PolarAngleLegend[Right, ImageSize → 0.4 * 72],
     ecLeg = EccentricityLegend[Left, ImageSize → 0.4 * 72]},
    {ecImg, paImg, ecLeg, paLeg,
     Graphics[
      {Inset[ecLeg, ImageScaled[{0.495, 0}], ImageScaled[{1, 0}]],
       Inset[paLeg, ImageScaled[{0.505, 0}], ImageScaled[{0, 0}]],
       Inset[ecImg, ImageScaled[{0, 1}], ImageScaled[{0, 1}]],
       Inset[paImg, ImageScaled[{1, 1}], ImageScaled[{1, 1}]]},
      ImageSize → {3.4, 1.15} * 72,
      ImagePadding → 2000]}]];
Image[figData20SS, ImageResolution → 150]
```

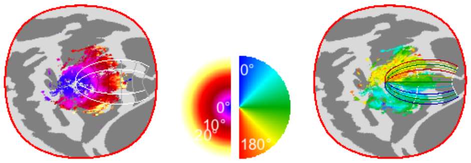
0°
10°
20°
0°
180°

6.4.3. Aggregate in Voronoi-Corrected Topology

```
{figData20VoronoiEcc, figData20VoronoiPol,
   eccLegend, polLegend, figData20Voronoi} = With[
   {simres = data10[VoronoiRegister, OurSchiraModel],
    agg = data20[Aggregate],
    schira = sim10[SchiraModel]},
   With[
    {paImg = Show[
       CorticalPlot[
        SelectPolarAngle[DistortData[agg, simres], 18, {0, 20}],
        CortexOutline → $Data20CorrectedOutline,
        ColorFunction → $PolarAngleColorFn,
        V1Outline → None,
        AnatomyData → DistortData[LocalAnatomy, simres],
        ImageSize → 1.15 * 72],
       SchiraLinePlot[
        schira,
        PolarAngleStyleFunction →
          ({Thickness[0.006], Darker[$PolarAngleColorFn[#]]} &),
        EccentricityStyleFunction → ({Thickness[0.004], Dashed, White} &),
        EccentricityLines → {10, 90},
        Axes → None,
        Frame → False]],
     ecImg = Show[
       CorticalPlot[
        SelectEccentricity[DistortData[agg, simres], 18, {0, 20}],
        CortexOutline → $Data20CorrectedOutline,
        ColorFunction → $EccentricityColorFn,
        V1Outline → None,
        AnatomyData → DistortData[LocalAnatomy, simres],
        ImageSize → 1.15 * 72],
       SchiraLinePlot[
        schira,
        EccentricityStyleFunction →
          ({Thickness[0.006], Darker[$EccentricityColorFn[#]]} &),
        PolarAngleStyleFunction → ({Thickness[0.004], White} &),
        PolarAngleLines → {{0, 180}, None, {0, 180}},
        Axes → None,
        Frame → False]],
     paLeg = PolarAngleLegend[Right, ImageSize → 0.4 * 72],
     ecLeg = EccentricityLegend[Left, ImageSize → 0.4 * 72]},
    {ecImg, paImg, ecLeg, paLeg,
     Graphics[
      {Inset[ecLeg, ImageScaled[{0.495, 0}], ImageScaled[{1, 0}]],
       Inset[paLeg, ImageScaled[{0.505, 0}], ImageScaled[{0, 0}]],
       Inset[ecImg, ImageScaled[{0, 1}], ImageScaled[{0, 1}]],
       Inset[paImg, ImageScaled[{1, 1}], ImageScaled[{1, 1}]]},
      ImageSize → {3.4, 1.15} * 72,
      ImagePadding → 2000]}]];
Image[figData20SS, ImageResolution → 150]
```

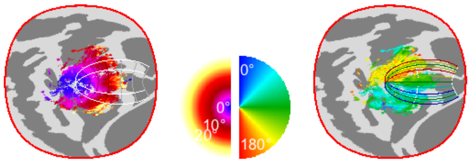
0°
10°
20°
0°
180°

6.5. Leave-One-Out Predictions

6.5.1. Spring Space LOO Error Plots on the Cortical Surface

```
{figLOOErrsEcc, figLOOErrsPol, figLOOErrsLegend, figLOOErrs} = With[
   {looErr = data10[CollectLOOErrors,
      #[SmoothPredictionGaussian] & /@ simsLOO,
      {1.25, 8.75}],
    ecTicks = {0, 2, 4},
    paTicks = {0, 25, 50}},
   With[
    {paerr = CorticalPlot[
       Map[Append[#[[1]],
          Median[Abs[#[[2, All, 1, 1]] - #[[2, All, 1, 2]]]]] &, looErr],
       CortexOutline → $Data10FSAverageOutline,
       ColorFunction → (Which[
            # > Last@paTicks, Darker[Green],
            # > 0, Blend[{White, Darker@Green}, # / Last@paTicks],
            # < -Last@paTicks, Red,
            True, Blend[{White, Red}, Abs[# / Last@paTicks]]] &),
       ImageSize → 1.15 * 72],
     ecerr = CorticalPlot[
       Map[Append[#[[1]],
          Median[Abs[#[[2, All, 2, 1]] - #[[2, All, 2, 2]]]]] &, looErr],
       CortexOutline → $Data10FSAverageOutline,
       ColorFunction → (If[# > Last@ecTicks,
            Darker[Green],
            Blend[{White, Darker@Green}, # / Last@ecTicks]] &),
       ImageSize → 1.15 * 72],
     legend = With[
       {emx = Last@ecTicks, pmx = Last@paTicks},
       ArrayPlot[
        Reverse@Table[{k, k}, {k, 0, 1, 0.01}],
        DataRange → {{0, 1}, {0, 1}},
        ImageSize → 0.9 * 72,
        AspectRatio → 5,
        Frame → {{True, True}, {False, False}},
        ColorFunction → (Blend[{White, Darker@Green}, #] &),
        FrameTicks → {{Table[{k / emx, k}, {k, {0, 2, 4}}],
           Table[{k / pmx, k}, {k, {0, 25, 50}}]}, {None, None}},
        FrameLabel → {{None, None}, {"Eccentricity", "Polar Angle"}},
        PlotLabel → Style["Error  (°)", FontFamily → "Arial", FontSize → 10],
        BaseStyle → Directive[10, FontFamily → "Arial"]]]},
    {ecerr, paerr, legend,
     Graphics[
      {Inset[legend, ImageScaled[{0.5, 0}], ImageScaled[{0.5, 0}]],
       Inset[ecerr, ImageScaled[{0, 0.55}], ImageScaled[{0, 0.5}]],
       Inset[paerr, ImageScaled[{1, 0.55}], ImageScaled[{1, 0.5}]]},
      ImageSize → {3.4, 1.25} * 72,
      ImagePadding → 2000]}]];
Image[figLOOErrs, ImageResolution → 150]
```

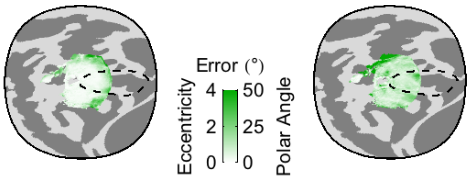

6.5.2. Spring Space Line Plots of LOO Errors

```
{figLOOErrorLinesEcc1, figLOOErrorLinesEcc2,
   figLOOErrorLinesEcc3, figLOOErrorLinesEccAll,
   figLOOErrorLinesPol1, figLOOErrorLinesPol2,
   figLOOErrorLinesPol3, figLOOErrorLinesPolAll} = With[
   {looErr = data10[CollectLOOErrors,
      #[SmoothPredictionFull] & /@ simsLOO,
      {1.25, 8.75}]},
   With[
    {ecplots = With[
       {eccerrTmp = Reap[
           Scan[
            Sow[#[[1]], {0, Round@#[[2]]}] &,
            Flatten[looErr[[All, 2, All, 2 ;; 3]], 1]],
           {1, 2, 3, 0},
           Apply[Sequence, #2] &
          ][[2]],
        fillColors = {
          Blend[{White, Pink}, 0.2],
          Blend[{White, Green}, 0.2],
          Blend[{White, Blue}, 0.2],
          Blend[{White, Black}, 0.2]}},
       With[
        {ecErr = Map[
           Reap[
              Scan[
               If[Length@# ≥ 3,
                 Sow[{Mean[#[[All, 1]]], Abs[#[[All, 1]] - #[[All, 2]]]}]] &,
               Split[
                Sort[#, #1[[1]] < #2[[1]] &],
                Round[#1[[1]] / (10 / 200)] == Round[#2[[1]] / (10 / 200)] &]]
             ][[2, 1]] &,
           eccerrTmp]},
        With[
         {ecErrQuartiles =
           Map[Map[Prepend[Quartiles[#[[2]]], #[[1]]] &, #] &, ecErr]},
         Block[{t, A, B, C, D, F},
          With[
           {mdls = Table[
              NonlinearModelFit[
               ecErrQuartiles[[k, All, {1, dim}]],
               A + B * t + C * t^2 + D * t^3 + F * t^4,
               {A, B, C, D, F},
               t],
              {k, 1, Length@ecErrQuartiles},
              {dim, {2, 3, 4}}]},
           MapThread[
```

```
        Function[{models, fillColor, qrtls},
         Plot[
          Evaluate[#[t] & /@ models],
          {t, 1.25, 8.75},
          PlotStyle → {{Thin, fillColor}, Black, {Thin, fillColor}},
          Filling → {1 → {{2}, fillColor}, 3 → {{2}, fillColor}},
          Epilog → {Gray, Line[qrtls[[All, {1, 3}]]]},
          BaseStyle → Directive[10, FontFamily → "Arial"],
          PlotRange → {{1, 9}, {0, 4}},
          Axes → None,
          Frame → Table[{True, False}, {2}],
          FrameLabel → {{"Absolute Error [deg]", None},
            {"Predicted Eccentricity [deg]", None}},
          FrameTicks → {{{0, 1, 2, 3, 4}, None}, {{1, 3, 5, 7, 9}, None}},
          AspectRatio → 0.75,
          ImageSize → 2.25 * 72]],
        {mdls, fillColors, ecErrQuartiles}]]]]]],
paplots = With[
  {polerrTmp = Reap[
      Scan[
       Sow[#[[1]], {0, Round@#[[2]]}] &,
       Flatten[looErr[[All, 2, All, {1, 3}]], 1]],
      {1, 2, 3, 0},
      Apply[Sequence, #2] &
     ][[2]],
   fillColors = {
     Blend[{White, Pink}, 0.2],
     Blend[{White, Green}, 0.2],
     Blend[{White, Blue}, 0.2],
     Blend[{White, Black}, 0.2]}},
  With[
   {paErr = Map[
      Reap[
         Scan[
          If[Length@# ≥ 3,
            Sow[{Mean[#[[All, 1]]], Abs[#[[All, 1]] - #[[All, 2]]]}]] &,
          Split[
           Sort[#, #1[[1]] < #2[[1]] &],
           Round[#1[[1]] / (180 / 200)] == Round[#2[[1]] / (180 / 200)] &]]
        ][[2, 1]] &,
      polerrTmp]},
   With[
    {paErrQuartiles = Map[
       Select[
         Map[If[Length[#[[2]]] > 2,
            Prepend[Quartiles[#[[2]]], #[[1]]], None] &, #],
         ListQ
        ] &,
       paErr]},
    Block[{t, A, B, C, D, F},
     With[
      {mdls = Table[
         NonlinearModelFit[
          paErrQuartiles[[k, All, {1, dim}]],
          A + B * t + C * t^2 + D * t^3 + F * t^4,
```

```
                 {A, B, C, D, F},
                 t],
                {k, 1, Length@paErrQuartiles},
                {dim, {2, 3, 4}}]},
            MapThread[
             Function[{models, fillColor, qrtls},
              Plot[
               Evaluate[#[t] & /@ models],
               {t, 0, 180},
               PlotStyle → {{Thin, fillColor}, Black, {Thin, fillColor}},
               Filling → {1 → {{2}, fillColor}, 3 → {{2}, fillColor}},
               Epilog → {Gray, Line[qrtls[[All, {1, 3}]]]},
               PlotRange → {0, 50},
               BaseStyle → Directive[10, FontFamily → "Arial"],
               Axes → None,
               Frame → Table[{True, False}, {2}],
               FrameLabel → {{"Absolute Error [deg]", None},
                 {"Predicted Polar Angle [deg]", None}},
               FrameTicks → {{{0, 10, 20, 30, 40, 50}, None},
                 {{0, 45, 90, 135, 180}, None}},
               AspectRatio → 0.75,
               ImageSize → 2.25 * 72]],
             {mdls, fillColors, paErrQuartiles}]]]]]]},
    Join[ecplots, paplots]]];
Image[
 GraphicsGrid[
  Transpose[
   {{figLOOErrorLinesEcc1, figLOOErrorLinesEcc2,
     figLOOErrorLinesEcc3, figLOOErrorLinesEccAll},
    {figLOOErrorLinesPol1, figLOOErrorLinesPol2,
     figLOOErrorLinesPol3, figLOOErrorLinesPolAll}}]],
 ImageResolution → 150]
```

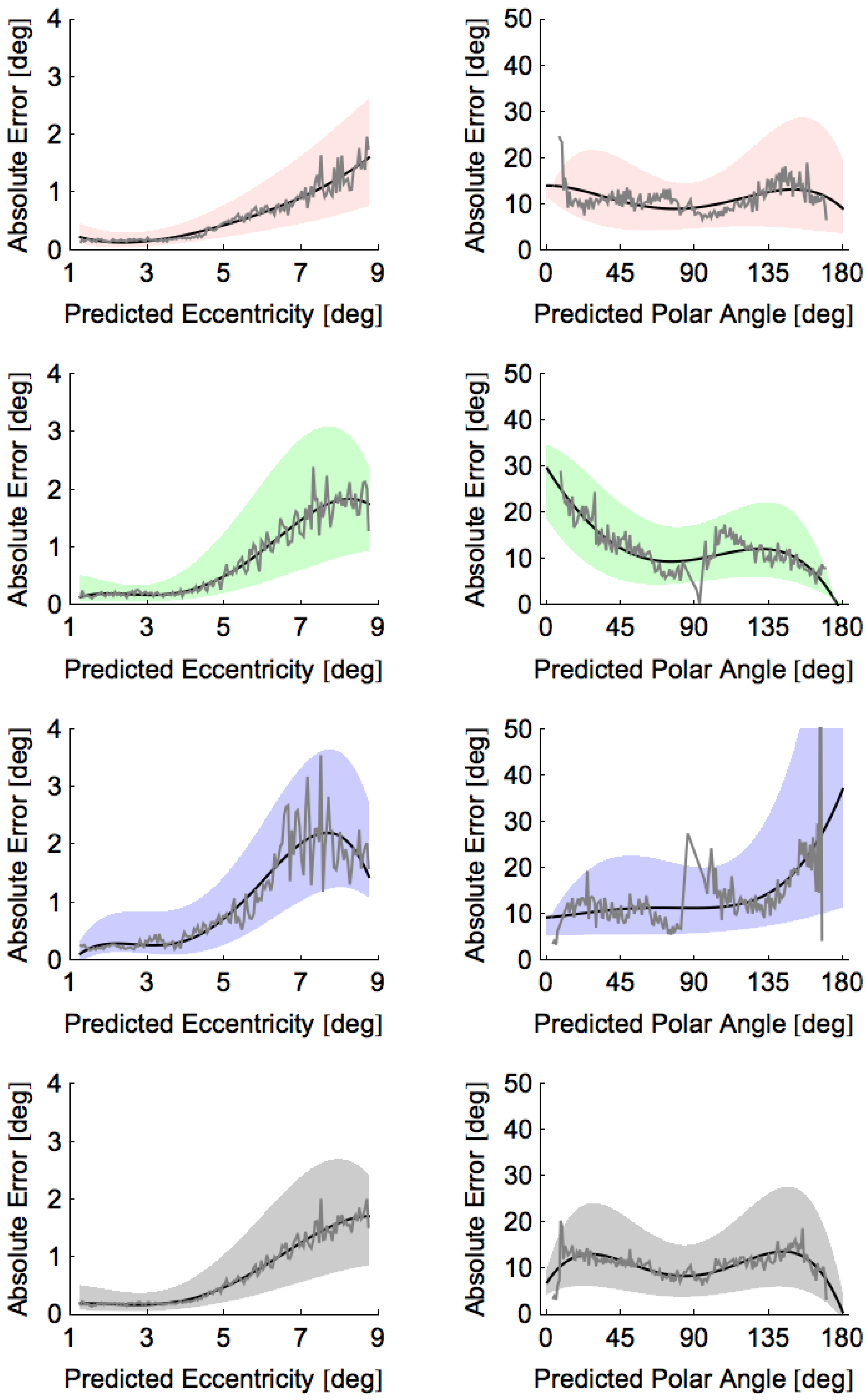

Absolute Error [deg]
Predicted Eccentricity [deg]
Absolute Error [deg]
Predicted Polar Angle [deg]
Absolute Error [deg]
Predicted Eccentricity [deg]
Absolute Error [deg]
Predicted Polar Angle [deg]
Absolute Error [deg]
Predicted Eccentricity [deg]
Absolute Error [deg]
Predicted Polar Angle [deg]
Absolute Error [deg]
Predicted Eccentricity [deg]
Absolute Error [deg]
Predicted Polar Angle [deg]

6.5.3. Spring Space Histograms

Here, we can see histograms of the leave-one-out error in the predictions of the polar angle and eccentricity.

```
figErrHistograms = GraphicsGrid[
   With[
    {loo = data10[CollectLOOErrors,
       #[SmoothPredictionGaussian] & /@ simsLOO,
       {1.25, 8.75}]},
    With[
     {ecerr = Reap[
         Scan[
          Sow[#[[1]], {0, Round@#[[2]]}] &,
          Flatten[loo[[All, 2, All, 2 ;; 3]], 1]],
         {0, 1, 2, 3},
         Apply[Sequence, #2] &
        ][[2]],
      polerr = Reap[
         Scan[
          Sow[#[[1]], {0, Round@#[[2]]}] &,
          Flatten[loo[[All, 2, All, {1, 3}]], 1]],
         {0, 1, 2, 3},
         Apply[Sequence, #2] &
        ][[2]]},
     List[
      MapThread[
       SmoothDensityHistogram[
         #1,
         ImageSize → 3.25 * 72,
         AspectRatio → 1,
         BaseStyle → Directive[10, FontFamily → "Arial"],
         ColorFunction → (If[# > 0.2, Black, Blend[{White, Black}, # / 0.2]] &),
         Axes → None,
         PlotRange → {{1.25, 8.75}, {1.25, 8.75}},
         PlotLabel →
          Style[#2, FontFamily → "Arial", FontSize → 10, FontWeight → Bold],
         Frame → {{True, False}, {True, False}},
         FrameLabel → {
            {"Observed Eccentricity (°)", None},
            {"Predicted Eccentricity (°)", None}}
        ] &,
       {ecerr, {"All", "V1", "V2", "V3"}}],
      MapThread[
       SmoothDensityHistogram[
         #1,
         ImageSize → 3.25 * 72,
         AspectRatio → 1,
         BaseStyle → Directive[10, FontFamily → "Arial"],
         ColorFunction → (If[# > 0.2, Black, Blend[{White, Black}, # / 0.2]] &),
         Axes → None,
         PlotRange → {{0, 180}, {0, 180}},
         PlotLabel →
          Style[#2, FontFamily → "Arial", FontSize → 10, FontWeight → Bold],
```

```
            Frame → {{True, False}, {True, False}},
            FrameLabel → {{"Observed Polar Angle (°)", None},
              {"Predicted Polar Angle (°)", None}}
          ] &,
        {polerr, {"All", "V1", "V2", "V3"}}]]]]];
Image[figErrHistograms, ImageResolution → 150]
```

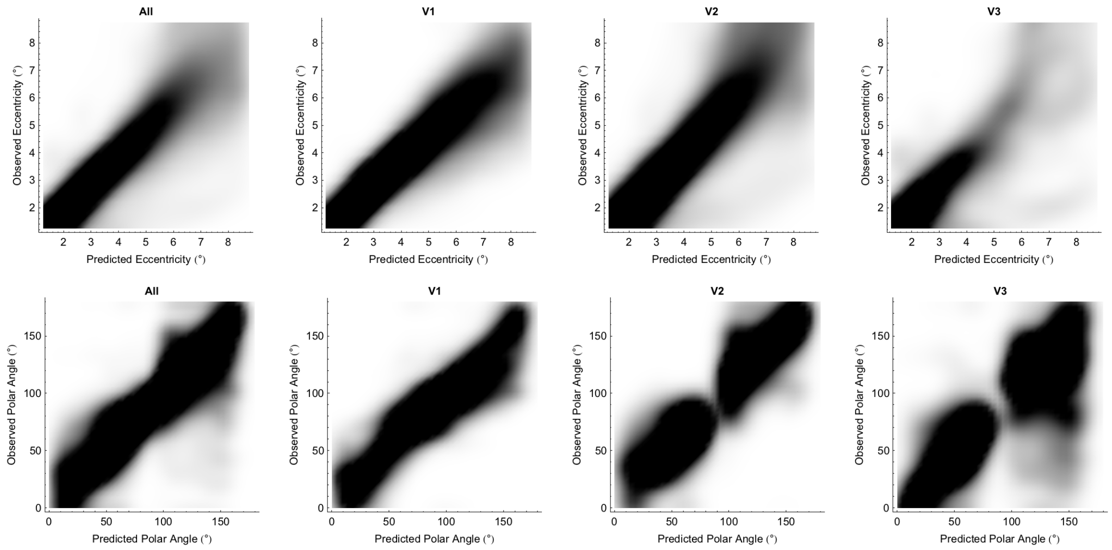

6.5.4. Voronoi LOO Error Plots on Cortical Surface

```
{figVoronoiLOOErrsEcc, figVoronoiLOOErrsPol,
   figVoronoiLOOErrsLegend, figVoronoiLOOErrs} = With[
   {looErr = data10[CollectLOOErrors,
      data10[VoronoiPrediction, LOO[#], OurSchiraModel] & /@ data10[Subjects],
      {1.25, 8.75}],
    ecTicks = {0, 2, 4},
    paTicks = {0, 25, 50}},
   With[
    {paerr = CorticalPlot[
       Map[Append[#[[1]],
          Median[Abs[#[[2, All, 1, 1]] - #[[2, All, 1, 2]]]]] &, looErr],
       CortexOutline → $Data10FSAverageOutline,
       ColorFunction → (Which[
           # > Last@paTicks, Darker[Green],
           # > 0, Blend[{White, Darker@Green}, # / Last@paTicks],
           # < -Last@paTicks, Red,
           True, Blend[{White, Red}, Abs[# / Last@paTicks]]] &),
       ImageSize → 1.15 * 72],
     ecerr = CorticalPlot[
       Map[Append[#[[1]],
          Median[Abs[#[[2, All, 2, 1]] - #[[2, All, 2, 2]]]]] &, looErr],
       CortexOutline → $Data10FSAverageOutline,
       ColorFunction → (If[# > Last@ecTicks,
           Darker[Green],
           Blend[{White, Darker@Green}, # / Last@ecTicks]] &),
       ImageSize → 1.15 * 72],
     legend = With[
       {emx = Last@ecTicks, pmx = Last@paTicks},
       ArrayPlot[
        Reverse@Table[{k, k}, {k, 0, 1, 0.01}],
        DataRange → {{0, 1}, {0, 1}},
        ImageSize → 0.9 * 72,
        AspectRatio → 5,
        Frame → {{True, True}, {False, False}},
        ColorFunction → (Blend[{White, Darker@Green}, #] &),
        FrameTicks → {{Table[{k / emx, k}, {k, {0, 2, 4}}],
           Table[{k / pmx, k}, {k, {0, 25, 50}}]}, {None, None}},
        FrameLabel → {{None, None}, {"Eccentricity", "Polar Angle"}},
        PlotLabel → Style["Error (°)", FontFamily → "Arial", FontSize → 10],
        BaseStyle → Directive[10, FontFamily → "Arial"]]]},
    {ecerr, paerr, legend,
     Graphics[
      {Inset[legend, ImageScaled[{0.5, 0}], ImageScaled[{0.5, 0}]],
       Inset[ecerr, ImageScaled[{0, 0.55}], ImageScaled[{0, 0.5}]],
       Inset[paerr, ImageScaled[{1, 0.55}], ImageScaled[{1, 0.5}]]},
      ImageSize → {3.4, 1.25} * 72,
      ImagePadding → 2000]}]];
Image[figVoronoiLOOErrs, ImageResolution → 150]
```

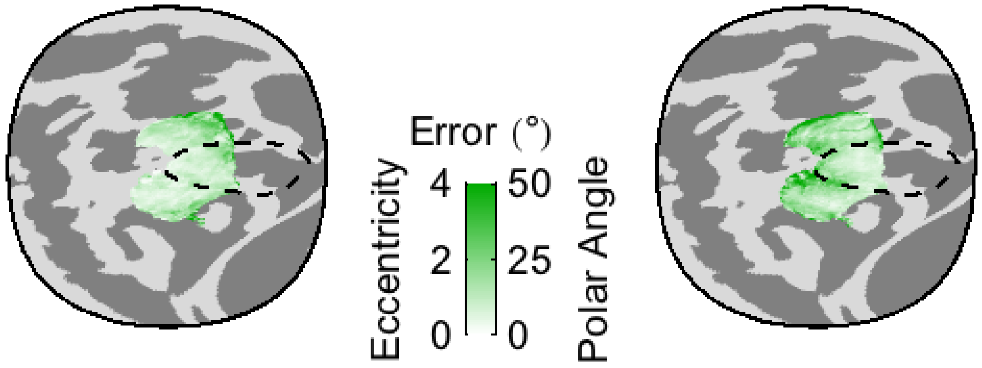

6.5.5. Voronoi Space Line Plots of LOO Errors

```
{figVoronoiLOOErrorLinesEcc1, figVoronoiLOOErrorLinesEcc2,
   figVoronoiLOOErrorLinesEcc3, figVoronoiLOOErrorLinesEccAll,
   figVoronoiLOOErrorLinesPol1, figVoronoiLOOErrorLinesPol2,
   figVoronoiLOOErrorLinesPol3, figVoronoiLOOErrorLinesPolAll} = With[
   {looErr = data10[CollectLOOErrors,
      data10[VoronoiPrediction, LOO[#], OurSchiraModel] & /@ data10[Subjects],
      {1.25, 8.75}]},
   With[
    {ecplots = With[
       {eccerrTmp = Reap[
           Scan[
            Sow[#[[1]], {0, Round@#[[2]]}] &,
            Flatten[looErr[[All, 2, All, 2 ;; 3]], 1]],
           {1, 2, 3, 0},
           Apply[Sequence, #2] &
          ][[2]],
        fillColors = {
          Blend[{White, Pink}, 0.2],
          Blend[{White, Green}, 0.2],
          Blend[{White, Blue}, 0.2],
          Blend[{White, Black}, 0.2]}},
       With[
        {ecErr = Map[
           Reap[
              Scan[
               If[Length@# ≥ 3,
                 Sow[{Mean[#[[All, 1]]], Abs[#[[All, 1]] - #[[All, 2]]]}]] &,
               Split[
                Sort[#, #1[[1]] < #2[[1]] &],
                Round[#1[[1]] / (10 / 200)] == Round[#2[[1]] / (10 / 200)] &]]
             ][[2, 1]] &,
           eccerrTmp]},
        With[
         {ecErrQuartiles =
           Map[Map[Prepend[Quartiles[#[[2]]], #[[1]]] &, #] &, ecErr]},
         Block[{t, A, B, C, D, F},
          With[
           {mdls = Table[
              NonlinearModelFit[
               ecErrQuartiles[[k, All, {1, dim}]],
```

```
              A + B * t + C * t^2 + D * t^3 + F * t^4,
              {A, B, C, D, F},
              t],
             {k, 1, Length@ecErrQuartiles},
             {dim, {2, 3, 4}}]]},
         MapThread[
          Function[{models, fillColor, qrtls},
           Plot[
            Evaluate[#[t] & /@ models],
            {t, 1.25, 8.75},
            PlotStyle → {{Thin, fillColor}, Black, {Thin, fillColor}},
            Filling → {1 → {{2}, fillColor}, 3 → {{2}, fillColor}},
            Epilog → {Gray, Line[qrtls[[All, {1, 3}]]]},
            BaseStyle → Directive[10, FontFamily → "Arial"],
            PlotRange → {{1, 9}, {0, 4}},
            Axes → None,
            Frame → Table[{True, False}, {2}],
            FrameLabel → {{"Absolute Error [deg]", None},
              {"Predicted Eccentricity [deg]", None}},
            FrameTicks → {{{0, 1, 2, 3, 4}, None}, {{1, 3, 5, 7, 9}, None}},
            AspectRatio → 0.75,
            ImageSize → 2.25 * 72]],
          {mdls, fillColors, ecErrQuartiles}]]]]]],
   paplots = With[
     {polerrTmp = Reap[
         Scan[
          Sow[#[[1]], {0, Round@#[[2]]}] &,
          Flatten[looErr[[All, 2, All, {1, 3}]], 1]],
         {1, 2, 3, 0},
         Apply[Sequence, #2] &
        ][[2]],
      fillColors = {
        Blend[{White, Pink}, 0.2],
        Blend[{White, Green}, 0.2],
        Blend[{White, Blue}, 0.2],
        Blend[{White, Black}, 0.2]}},
     With[
      {paErr = Map[
         Reap[
            Scan[
             If[Length@# ≥ 3,
               Sow[{Mean[#[[All, 1]]], Abs[#[[All, 1]] - #[[All, 2]]]}]] &,
             Split[
              Sort[#, #1[[1]] < #2[[1]] &],
              Round[#1[[1]] / (180 / 200)] == Round[#2[[1]] / (180 / 200)] &]]
           ][[2, 1]] &,
         polerrTmp]},
      With[
       {paErrQuartiles = Map[
          Select[
            Map[If[Length[#[[2]]] > 2,
               Prepend[Quartiles[#[[2]]], #[[1]]], None] &, #],
            ListQ
           ] &,
          paErr]},
```

```
          Block[{t, A, B, C, D, F},
           With[
            {mdls = Table[
               NonlinearModelFit[
                paErrQuartiles[[k, All, {1, dim}]],
                A + B * t + C * t^2 + D * t^3 + F * t^4,
                {A, B, C, D, F},
                t],
               {k, 1, Length@paErrQuartiles},
               {dim, {2, 3, 4}}]},
            MapThread[
             Function[{models, fillColor, qrtls},
              Plot[
               Evaluate[#[t] & /@ models],
               {t, 0, 180},
               PlotStyle → {{Thin, fillColor}, Black, {Thin, fillColor}},
               Filling → {1 → {{2}, fillColor}, 3 → {{2}, fillColor}},
               Epilog → {Gray, Line[qrtls[[All, {1, 3}]]]},
               PlotRange → {0, 50},
               BaseStyle → Directive[10, FontFamily → "Arial"],
               Axes → None,
               Frame → Table[{True, False}, {2}],
               FrameLabel → {{"Absolute Error [deg]", None},
                 {"Predicted Polar Angle [deg]", None}},
               FrameTicks → {{{0, 10, 20, 30, 40, 50}, None},
                 {{0, 45, 90, 135, 180}, None}},
               AspectRatio → 0.75,
               ImageSize → 2.25 * 72]],
             {mdls, fillColors, paErrQuartiles}]]]]]]},
    Join[ecplots, paplots]]];
Image[
 GraphicsGrid[
  Transpose[
   {{figVoronoiLOOErrorLinesEcc1, figVoronoiLOOErrorLinesEcc2,
     figVoronoiLOOErrorLinesEcc3, figVoronoiLOOErrorLinesEccAll},
    {figVoronoiLOOErrorLinesPol1, figVoronoiLOOErrorLinesPol2,
     figVoronoiLOOErrorLinesPol3, figVoronoiLOOErrorLinesPolAll}}]],
 ImageResolution → 150]
```

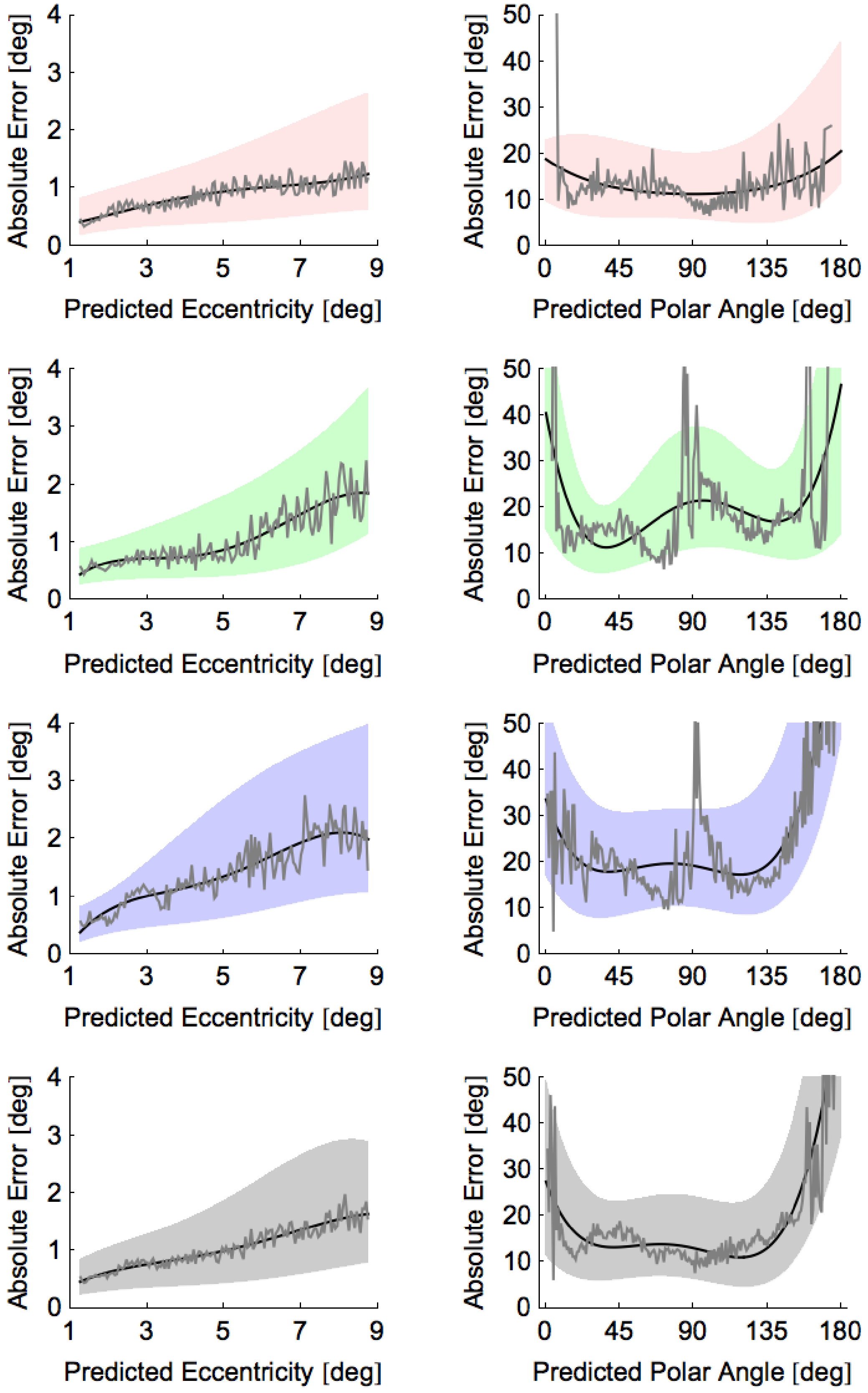
Absolute Error [deg]
Predicted Eccentricity [deg]
Predicted Polar Angle [deg]
0
1
2
3
4
1
3
5
7
9
0
10
20
30
40
50
0
45
90
135
180

6.5.6. Voronoi Space Histograms

Here, we can see histograms of the leave-one-out error in the predictions of the polar angle and eccentricity.

```
figErrVoronoiHistograms = GraphicsGrid[
   With[
    {loo = data10[CollectLOOErrors,
       data10[VoronoiPrediction, LOO[#], OurSchiraModel] & /@ data10[Subjects],
       {1.25, 8.75}]},
    With[
     {ecerr = Reap[
         Scan[
          Sow[#[[1]], {0, Round@#[[2]]}] &,
          Flatten[loo[[All, 2, All, 2 ;; 3]], 1]],
         {0, 1, 2, 3},
         Apply[Sequence, #2] &
        ][[2]],
      polerr = Reap[
         Scan[
          Sow[#[[1]], {0, Round@#[[2]]}] &,
          Flatten[loo[[All, 2, All, {1, 3}]], 1]],
         {0, 1, 2, 3},
         Apply[Sequence, #2] &
        ][[2]]},
     List[
      MapThread[
       SmoothDensityHistogram[
         #1,
         ImageSize → 3.25 * 72,
         AspectRatio → 1,
         BaseStyle → Directive[10, FontFamily → "Arial"],
         ColorFunction → (If[# > 0.2, Black, Blend[{White, Black}, # / 0.2]] &),
         Axes → None,
         PlotRange → {{1.25, 8.75}, {1.25, 8.75}},
         PlotLabel →
          Style[#2, FontFamily → "Arial", FontSize → 10, FontWeight → Bold],
         Frame → {{True, False}, {True, False}},
         FrameLabel → {
           {"Observed Eccentricity (°)", None},
           {"Predicted Eccentricity (°)", None}}
        ] &,
       {ecerr, {"All", "V1", "V2", "V3"}}],
      MapThread[
       SmoothDensityHistogram[
         #1,
         ImageSize → 3.25 * 72,
         AspectRatio → 1,
         BaseStyle → Directive[10, FontFamily → "Arial"],
         ColorFunction → (If[# > 0.2, Black, Blend[{White, Black}, # / 0.2]] &),
         Axes → None,
         PlotRange → {{0, 180}, {0, 180}},
         PlotLabel →
          Style[#2, FontFamily → "Arial", FontSize → 10, FontWeight → Bold],
```

```
          Frame → {{True, False}, {True, False}},
          FrameLabel → {{"Observed Polar Angle (°)", None},
            {"Predicted Polar Angle (°)", None}}
         ] &,
        {polerr, {"All", "V1", "V2", "V3"}}]]]]]];
Image[figErrVoronoiHistograms, ImageResolution → 150]
```

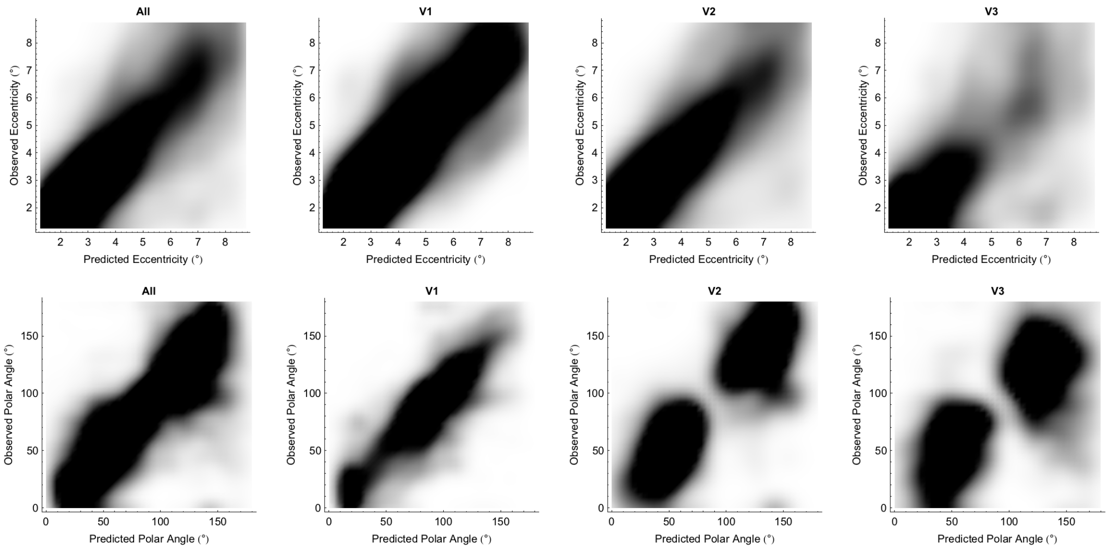

6.5.7. Errors plotted on the Schira Model

This image shows the LOO error projected onto the Schira model. To be precise, this takes the polar angle and eccentricity that is observed at a particular vertex, and plots the LOO error of that vertex at the position in the Schira model that it predicts for the observed polar angle and eccentricity.

```
With[
 {looErr = data10[CollectLOOErrors,
    #[SmoothPredictionGaussian] & /@ simsLOO,
    {1.25, 8.75}],
  sfn = SchiraFunction[OurSchiraModel]},
 With[
  {map = Map[
     group Ì Join[
       group[[1, 1 ;; 2]],
       Median /@ Transpose[group[[All, 3 ;; 4]]]],
     SplitBy[
      SortBy[
       Map[
        pt Ì With[
          {z = sfn[pt[[1, 1]], pt[[2, 1]], pt[[3]]]},
          {Re@z, Im@z,
           Abs[pt[[1, 1]] - pt[[1, 2]]],
           Abs[pt[[2, 1]] - pt[[2, 2]]]}],
        Flatten[looErr[[All, 2]], 1]],
       #[[1 ;; 2]] &],
      #[[1 ;; 2]] &]]},
  With[
```

```
{imgPol = With[
   {mx = 60},
   Graphics[
    {Inset[
      Show[
       {ListDensityPlot[
         map[[All, {1, 2, 3}]],
         ColorFunction → (If[# > mx, Green, Blend[{White, Green}, # / mx]] &),
         ColorFunctionScaling → False,
         PlotRange → Full,
         ImageSize → 3.25 * 72,
         AspectRatio → 1,
         Frame → False,
         Axes → False],
        SchiraLinePlotHash[OurSchiraModel, PolarAngle]}],
      ImageScaled[{0, 1}],
      ImageScaled[{0, 1}]],
     Inset[
      ArrayPlot[
       Reverse[Table[{k, k}, {k, 0, 1, 0.01}]],
       DataRange → {{0, 1}, {0, mx}},
       ColorFunction → (Blend[{White, Green}, #] &),
       Frame → {{False, True}, {False, False}},
       FrameTicks → {{False, Automatic}, {False, False}},
       BaseStyle → Directive[10, FontFamily → "Arial"],
       ImageSize → 0.5 * 72,
       AspectRatio → 3],
      ImageScaled[{1, 0}],
      ImageScaled[{1, 0}]]},
    ImagePadding → 10 000,
    ImageSize → {3.5, 3} * 72]],
 imgEcc = With[
   {mx = Max[map[[All, 4]]]},
   Graphics[
    {Inset[
      Show[
       {ListDensityPlot[
         map[[All, {1, 2, 4}]],
         ColorFunction → (If[# > mx, Green, Blend[{White, Green}, #]] &),
         ColorFunctionScaling → False,
         PlotRange → Full,
         ImageSize → 3.25 * 72,
         AspectRatio → 1,
         Frame → False,
         Axes → False],
        SchiraLinePlotHash[OurSchiraModel, Eccentricity]}],
      ImageScaled[{0, 1}],
      ImageScaled[{0, 1}]],
     Inset[
      ArrayPlot[
       Reverse[Table[{k, k}, {k, 0, 1, 0.01}]],
       DataRange → {{0, 1}, {0, mx}},
       ColorFunction → (Blend[{White, Green}, #] &),
       Frame → {{False, True}, {False, False}},
       FrameTicks → {{False, Automatic}, {False, False}},
```

```
            BaseStyle → Directive[10, FontFamily → "Arial"],
            ImageSize → 0.35 * 72,
            AspectRatio → 3],
           ImageScaled[{1, 0}],
           ImageScaled[{1, 0}]]},
         ImageSize → {3.5, 3} * 72,
         ImagePadding → 10 000]]},
     {imgPol, imgEcc}]]]
```

{ 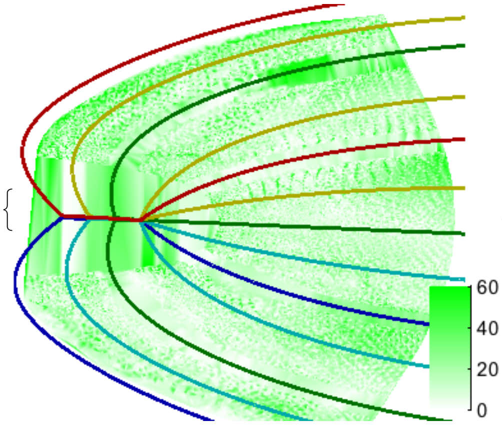

, 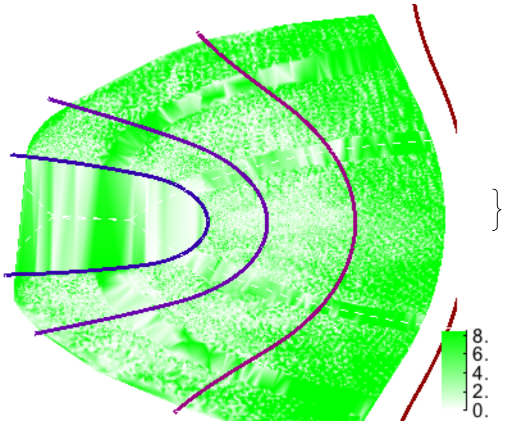

}

6.6. 20° Dataset Predictions

6.6.1. Spring Space 20° Error Plots on the Cortical Surface

```
{figExtErrsEcc, figExtErrsPol, figExtErrsLegend, figExtErrs} = With[
   {err = AggregateErrors[
      data20,
      sim10[SmoothPrediction],
      {1.25, 8.75},
      12],
    ecTicks = {0, 2, 4},
    paTicks = {0, 25, 50}},
   dbg = err;
   With[
    {paerr = CorticalPlot[
       Map[{#[[1, 1]], #[[1, 2]], Abs[#[[1, 3]] - #[[2, 3]]]} &, err[[1]]],
       CortexOutline → $Data10FSAverageOutline,
       ColorFunction → (Which[
           # > Last@paTicks, Darker[Green],
           # > 0, Blend[{White, Darker@Green}, # / Last@paTicks],
           # < -Last@paTicks, Red,
           True, Blend[{White, Red}, Abs[# / Last@paTicks]]] &),
       ImageSize → 1.15 * 72],
     ecerr = CorticalPlot[
       Map[{#[[1, 1]], #[[1, 2]], Abs[#[[1, 4]] - #[[2, 4]]]} &, err[[1]]],
       CortexOutline → $Data10FSAverageOutline,
       ColorFunction → (If[# > Last@ecTicks,
           Darker[Green],
           Blend[{White, Darker@Green}, # / Last@ecTicks]] &),
       ImageSize → 1.15 * 72],
     legend = With[
       {emx = Last@ecTicks, pmx = Last@paTicks},
       ArrayPlot[
        Reverse@Table[{k, k}, {k, 0, 1, 0.01}],
        DataRange → {{0, 1}, {0, 1}},
        ImageSize → 0.9 * 72,
        AspectRatio → 5,
        Frame → {{True, True}, {False, False}},
        ColorFunction → (Blend[{White, Darker@Green}, #] &),
        FrameTicks → {{Table[{k / emx, k}, {k, {0, 2, 4}}],
           Table[{k / pmx, k}, {k, {0, 25, 50}}]}, {None, None}},
        FrameLabel → {{None, None}, {"Eccentricity", "Polar Angle"}},
        PlotLabel → Style["Error (°)", FontFamily → "Arial", FontSize → 10],
        BaseStyle → Directive[10, FontFamily → "Arial"]]]},
    {ecerr, paerr, legend,
     Graphics[
      {Inset[legend, ImageScaled[{0.5, 0}], ImageScaled[{0.5, 0}]],
       Inset[ecerr, ImageScaled[{0, 0.55}], ImageScaled[{0, 0.5}]],
       Inset[paerr, ImageScaled[{1, 0.55}], ImageScaled[{1, 0.5}]]},
      ImageSize → {3.4, 1.25} * 72,
      ImagePadding → 2000]}]];
Image[figExtErrs, ImageResolution → 150]
```

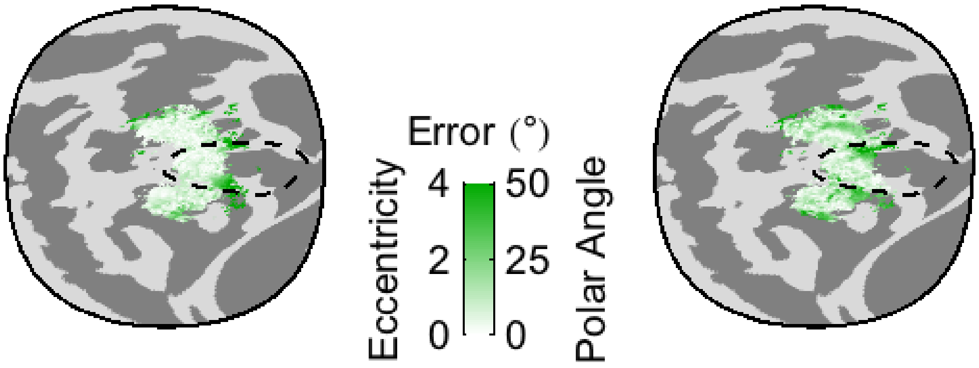

6.6.2. Spring Space Line Plots of 20° Errors

```
{figExtErrorLinesEcc1, figExtErrorLinesEcc2,
   figExtErrorLinesEcc3, figExtErrorLinesEccAll,
   figExtErrorLinesPol1, figExtErrorLinesPol2,
   figExtErrorLinesPol3, figExtErrorLinesPolAll} = With[
   {err = AggregateErrors[
      data20,
      sim10[SmoothPrediction],
      {1.25, 18.75},
      18]},
   With[
    {ecplots = With[
       {eccerrTmp = err[[{2, 3, 4, 1}, All, All, 4]],
        fillColors = {
          Blend[{White, Pink}, 0.2],
          Blend[{White, Green}, 0.2],
          Blend[{White, Blue}, 0.2],
          Blend[{White, Black}, 0.2]}},
       With[
        {ecErr = Map[
           Reap[
              Scan[
               If[Length@# ≥ 3,
                 Sow[{Mean[#[[All, 1]]], Abs[#[[All, 1]] - #[[All, 2]]]}]] &,
               Split[
                Sort[#, #1[[1]] < #2[[1]] &],
                Round[#1[[1]] / (10 / 200)] == Round[#2[[1]] / (10 / 200)] &]]
             ][[2, 1]] &,
           eccerrTmp]},
        With[
         {ecErrQuartiles =
           Map[Map[Prepend[Quartiles[#[[2]]], #[[1]]] &, #] &, ecErr]},
         Block[{t, A, B, C, D, F},
          With[
           {mdls = Table[
              NonlinearModelFit[
               ecErrQuartiles[[k, All, {1, dim}]],
               A + B * t + C * t^2 + D * t^3 + F * t^4,
               {A, B, C, D, F},
               t],
              {k, 1, Length@ecErrQuartiles},
```

```
          {dim, {2, 3, 4}}]],
       MapThread[
        Function[{models, fillColor, qrtls},
         Plot[
          Evaluate[#[t] & /@ models],
          {t, 1.25, 18.75},
          PlotStyle → {{Thin, fillColor}, Black, {Thin, fillColor}},
          Filling → {1 → {{2}, fillColor}, 3 → {{2}, fillColor}},
          Epilog → {Gray, Line[qrtls[[All, {1, 3}]]]},
          BaseStyle → Directive[10, FontFamily → "Arial"],
          PlotRange → {{1, 19}, {0, 8}},
          Axes → None,
          Frame → Table[{True, False}, {2}],
          FrameLabel → {{"Absolute Error [deg]", None},
            {"Predicted Eccentricity [deg]", None}},
          FrameTicks → {{{0, 1, 2, 3, 4} * 2, None}, {{5, 10, 15}, None}},
          AspectRatio → 0.75,
          ImageSize → 2.25 * 72]],
        {mdls, fillColors, ecErrQuartiles}]]]]]],
paplots = With[
  {polerrTmp = err[[{2, 3, 4, 1}, All, All, 3]],
   fillColors = {
     Blend[{White, Pink}, 0.2],
     Blend[{White, Green}, 0.2],
     Blend[{White, Blue}, 0.2],
     Blend[{White, Black}, 0.2]}},
  With[
   {paErr = Map[
      Reap[
        Scan[
         If[Length@# ≥ 3,
           Sow[{Mean[#[[All, 1]]], Abs[#[[All, 1]] - #[[All, 2]]]}]] &,
         Split[
          Sort[#, #1[[1]] < #2[[1]] &],
          Round[#1[[1]] / (180 / 200)] == Round[#2[[1]] / (180 / 200)] &]]
        ][[2, 1]] &,
      polerrTmp]},
   With[
    {paErrQuartiles = Map[
       Select[
         Map[If[Length[#[[2]]] > 2,
             Prepend[Quartiles[#[[2]]], #[[1]]], None] &, #],
         ListQ
        ] &,
       paErr]},
    Block[{t, A, B, C, D, F},
     With[
      {mdls = Table[
         NonlinearModelFit[
          paErrQuartiles[[k, All, {1, dim}]],
          A + B * t + C * t^2 + D * t^3 + F * t^4,
          {A, B, C, D, F},
          t],
         {k, 1, Length@paErrQuartiles},
         {dim, {2, 3, 4}}]],
```

```
          MapThread[
           Function[{models, fillColor, qrtls},
            Plot[
             Evaluate[#[t] & /@ models],
             {t, 0, 180},
             PlotStyle → {{Thin, fillColor}, Black, {Thin, fillColor}},
             Filling → {1 → {{2}, fillColor}, 3 → {{2}, fillColor}},
             Epilog → {Gray, Line[qrtls[[All, {1, 3}]]]},
             PlotRange → {0, 50},
             BaseStyle → Directive[10, FontFamily → "Arial"],
             Axes → None,
             Frame → Table[{True, False}, {2}],
             FrameLabel → {{"Absolute Error [deg]", None},
               {"Predicted Polar Angle [deg]", None}},
             FrameTicks → {{{0, 10, 20, 30, 40, 50}, None},
               {{0, 45, 90, 135, 180}, None}},
             AspectRatio → 0.75,
             ImageSize → 2.25 * 72]],
           {mdls, fillColors, paErrQuartiles}]]]]]]},
    Join[ecplots, paplots]]];
Image[
 GraphicsGrid[
  Transpose[
   {{figExtErrorLinesEcc1, figExtErrorLinesEcc2,
     figExtErrorLinesEcc3, figExtErrorLinesEccAll},
    {figExtErrorLinesPol1, figExtErrorLinesPol2,
     figExtErrorLinesPol3, figExtErrorLinesPolAll}}]],
 ImageResolution → 150]
```

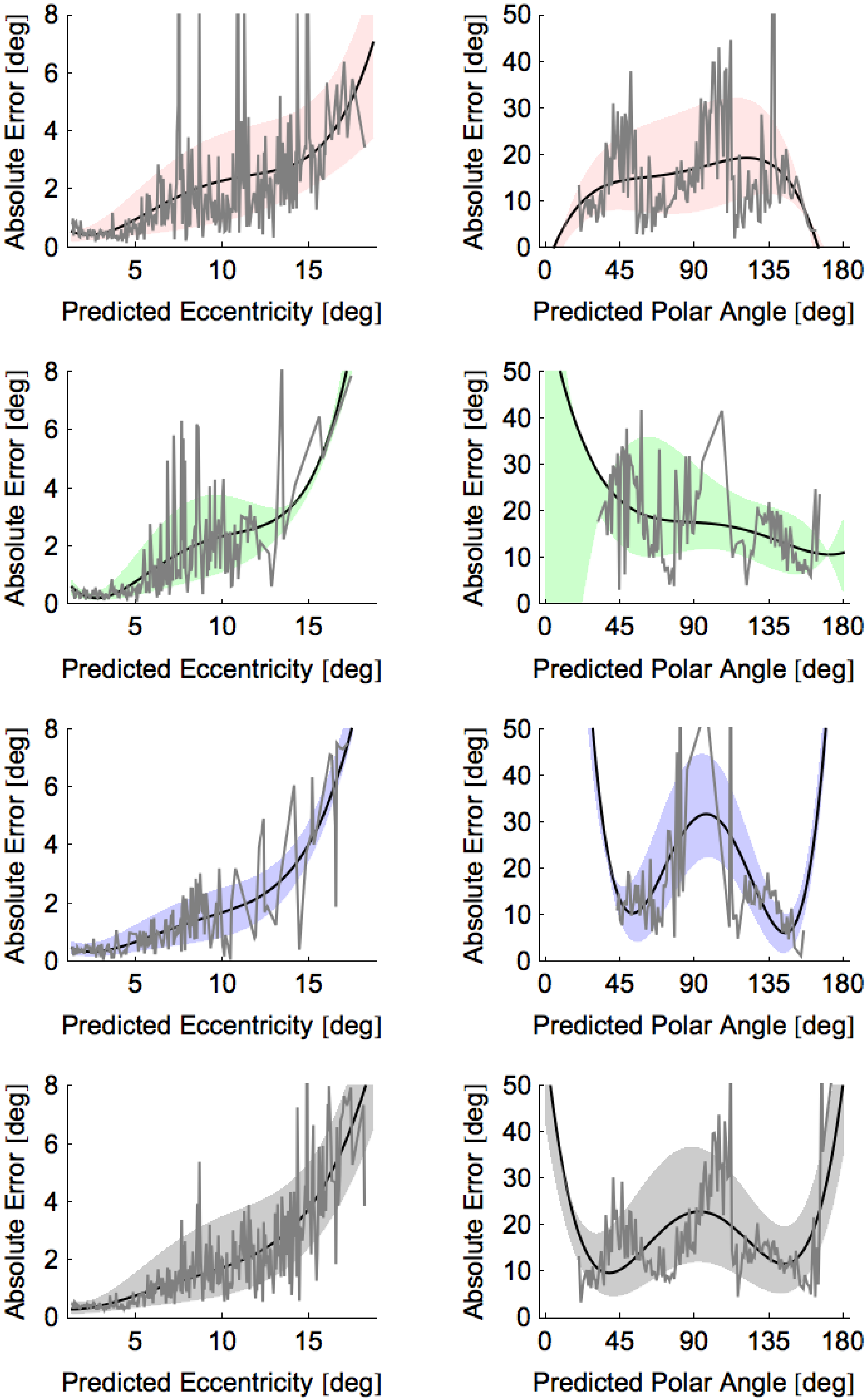
Absolute Error [deg]
Predicted Eccentricity [deg]
Absolute Error [deg]
Predicted Polar Angle [deg]
Absolute Error [deg]
Predicted Eccentricity [deg]
Absolute Error [deg]
Predicted Polar Angle [deg]
Absolute Error [deg]
Predicted Eccentricity [deg]
Absolute Error [deg]
Predicted Polar Angle [deg]
Absolute Error [deg]
Predicted Eccentricity [deg]
Absolute Error [deg]
Predicted Polar Angle [deg]

6.6.3. Spring Space Histograms

Here, we can see histograms of the leave-one-out error in the predictions of the polar angle and eccentricity.

```
figErr20Histograms = GraphicsGrid[
   With[
    {err = AggregateErrors[
       data20,
       sim10[SmoothPrediction],
       {1.25, 8.75},
       12]},
    With[
     {ecerr = err[[{2, 3, 4, 1}, All, All, 4]] /. (Rule[a_, b_] :> {a, b}),
      polerr = err[[{2, 3, 4, 1}, All, All, 3]] /. (Rule[a_, b_] :> {a, b})},
     List[
      MapThread[
       SmoothDensityHistogram[
         #1,
         ImageSize → 3.25 * 72,
         AspectRatio → 1,
         BaseStyle → Directive[10, FontFamily → "Arial"],
         ColorFunction → (If[# > 0.2, Black, Blend[{White, Black}, # / 0.2]] &),
         Axes → None,
         PlotRange → {{1.25, 8.75}, {1.25, 8.75}},
         PlotLabel →
          Style[#2, FontFamily → "Arial", FontSize → 10, FontWeight → Bold],
         Frame → {{True, False}, {True, False}},
         FrameLabel → {
           {"Observed Eccentricity (°)", None},
           {"Predicted Eccentricity (°)", None}}
        ] &,
       {ecerr, {"All", "V1", "V2", "V3"}}],
      MapThread[
       SmoothDensityHistogram[
         #1,
         ImageSize → 3.25 * 72,
         AspectRatio → 1,
         BaseStyle → Directive[10, FontFamily → "Arial"],
         ColorFunction → (If[# > 0.2, Black, Blend[{White, Black}, # / 0.2]] &),
         Axes → None,
         PlotRange → {{0, 180}, {0, 180}},
         PlotLabel →
          Style[#2, FontFamily → "Arial", FontSize → 10, FontWeight → Bold],
         Frame → {{True, False}, {True, False}},
         FrameLabel → {{"Observed Polar Angle (°)", None},
           {"Predicted Polar Angle (°)", None}}
        ] &,
       {polerr, {"All", "V1", "V2", "V3"}}]]]]];
Image[figErr20Histograms, ImageResolution → 150]
```

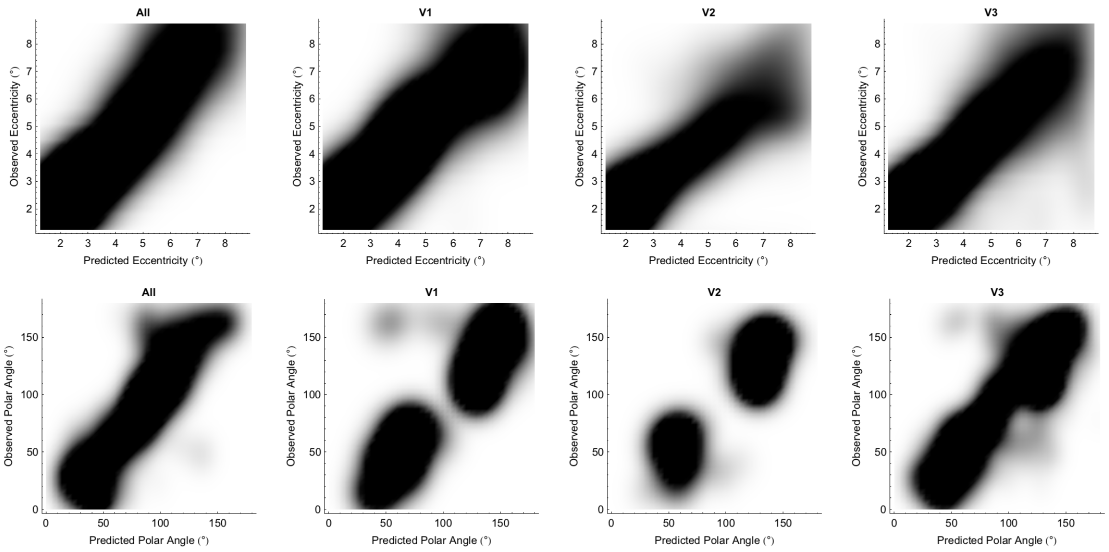
All
V1
V2
V3
Observed Eccentricity (°)
Predicted Eccentricity (°)
Observed Polar Angle (°)
Predicted Polar Angle (°)

6.6.4. Voronoi 20° Error Plots on Cortical Surface

```
{figVoronoiExtErrsEcc, figVoronoiExtErrsPol,
   figVoronoiExtErrsLegend, figVoronoiExtErrs} = With[
   {err = AggregateErrors[
      data20,
      sim10[SmoothPrediction],
      {1.25, 8.75}],
    ecTicks = {0, 2, 4},
    paTicks = {0, 25, 50}},
   With[
    {paerr = CorticalPlot[
       Map[{#[[1, 1]], #[[1, 2]], Abs[#[[1, 3]] - #[[2, 3]]]} &, err[[1]]],
       CortexOutline → $Data10FSAverageOutline,
       ColorFunction → (Which[
           # > Last@paTicks, Darker[Green],
           # > 0, Blend[{White, Darker@Green}, # / Last@paTicks],
           # < -Last@paTicks, Red,
           True, Blend[{White, Red}, Abs[# / Last@paTicks]]] &),
       ImageSize → 1.15 * 72],
     ecerr = CorticalPlot[
       Map[{#[[1, 1]], #[[1, 2]], Abs[#[[1, 4]] - #[[2, 4]]]} &, err[[1]]],
       CortexOutline → $Data10FSAverageOutline,
       ColorFunction → (If[# > Last@ecTicks,
           Darker[Green],
           Blend[{White, Darker@Green}, # / Last@ecTicks]] &),
       ImageSize → 1.15 * 72],
     legend = With[
       {emx = Last@ecTicks, pmx = Last@paTicks},
       ArrayPlot[
        Reverse@Table[{k, k}, {k, 0, 1, 0.01}],
        DataRange → {{0, 1}, {0, 1}},
        ImageSize → 0.9 * 72,
        AspectRatio → 5,
        Frame → {{True, True}, {False, False}},
        ColorFunction → (Blend[{White, Darker@Green}, #] &),
        FrameTicks → {{Table[{k / emx, k}, {k, {0, 2, 4}}],
           Table[{k / pmx, k}, {k, {0, 25, 50}}]}, {None, None}},
        FrameLabel → {{None, None}, {"Eccentricity", "Polar Angle"}},
        PlotLabel → Style["Error (°)", FontFamily → "Arial", FontSize → 10],
        BaseStyle → Directive[10, FontFamily → "Arial"]]]},
    {ecerr, paerr, legend,
     Graphics[
      {Inset[legend, ImageScaled[{0.5, 0}], ImageScaled[{0.5, 0}]],
       Inset[ecerr, ImageScaled[{0, 0.55}], ImageScaled[{0, 0.5}]],
       Inset[paerr, ImageScaled[{1, 0.55}], ImageScaled[{1, 0.5}]]},
      ImageSize → {3.4, 1.25} * 72,
      ImagePadding → 2000]}]];
Image[figExtErrs, ImageResolution → 150]
```

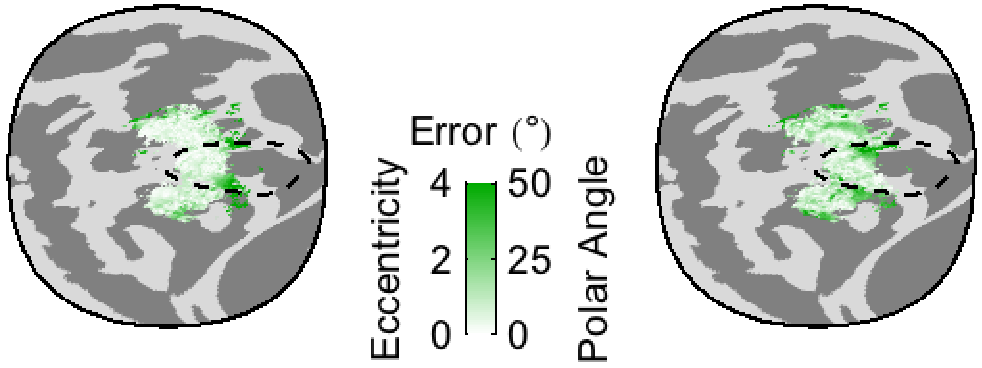

6.6.5. Voronoi Space Line Plots of 20° Errors

```
{figVoronoiExtErrorLinesEcc1, figVoronoiExtErrorLinesEcc2,
   figVoronoiExtErrorLinesEcc3, figVoronoiExtErrorLinesEccAll,
   figVoronoiExtErrorLinesPol1, figVoronoiExtErrorLinesPol2,
   figVoronoiExtErrorLinesPol3, figVoronoiExtErrorLinesPolAll} = With[
   {err = AggregateErrors[
      data20,
      sim10[SmoothPrediction],
      {1.25, 18.75},
      18]},
   With[
    {ecplots = With[
       {eccerrTmp = err[[{2, 3, 4, 1}, All, All, 4]] /. (Rule[a_, b_] :> {a, b}),
        fillColors = {
          Blend[{White, Pink}, 0.2],
          Blend[{White, Green}, 0.2],
          Blend[{White, Blue}, 0.2],
          Blend[{White, Black}, 0.2]}},
       With[
        {ecErr = Map[
           Reap[
              Scan[
               If[Length@# ≥ 3,
                 Sow[{Mean[#[[All, 1]]], Abs[#[[All, 1]] - #[[All, 2]]]}]] &,
               Split[
                Sort[#, #1[[1]] < #2[[1]] &],
                Round[#1[[1]] / (10 / 200)] == Round[#2[[1]] / (10 / 200)] &]]
             ][[2, 1]] &,
           eccerrTmp]},
        With[
         {ecErrQuartiles =
           Map[Map[Prepend[Quartiles[#[[2]]], #[[1]]] &, #] &, ecErr]},
         Block[{t, A, B, C, D, F},
          With[
           {mdls = Table[
              NonlinearModelFit[
               ecErrQuartiles[[k, All, {1, dim}]],
               A + B * t + C * t^2 + D * t^3 + F * t^4,
               {A, B, C, D, F},
               t],
```

```
            {k, 1, Length@ecErrQuartiles},
            {dim, {2, 3, 4}}]],
         MapThread[
          Function[{models, fillColor, qrtls},
           Plot[
            Evaluate[#[t] & /@ models],
            {t, 1.25, 18.75},
            PlotStyle → {{Thin, fillColor}, Black, {Thin, fillColor}},
            Filling → {1 → {{2}, fillColor}, 3 → {{2}, fillColor}},
            Epilog → {Gray, Line[qrtls[[All, {1, 3}]]]},
            BaseStyle → Directive[10, FontFamily → "Arial"],
            PlotRange → {{1, 19}, {0, 8}},
            Axes → None,
            Frame → Table[{True, False}, {2}],
            FrameLabel → {{"Absolute Error [deg]", None},
              {"Predicted Eccentricity [deg]", None}},
            FrameTicks → {{{0, 1, 2, 3, 4} * 2, None}, {{5, 10, 15}, None}},
            AspectRatio → 0.75,
            ImageSize → 2.25 * 72]],
          {mdls, fillColors, ecErrQuartiles}]]]]]],
paplots = With[
  {polerrTmp = err[[{2, 3, 4, 1}, All, All, 3]] /. (Rule[a_, b_] :→ {a, b}),
   fillColors = {
     Blend[{White, Pink}, 0.2],
     Blend[{White, Green}, 0.2],
     Blend[{White, Blue}, 0.2],
     Blend[{White, Black}, 0.2]}},
  With[
   {paErr = Map[
      Reap[
         Scan[
          If[Length@# ≥ 3,
            Sow[{Mean[#[[All, 1]]], Abs[#[[All, 1]] - #[[All, 2]]]}]] &,
          Split[
           Sort[#, #1[[1]] < #2[[1]] &],
           Round[#1[[1]] / (180 / 200)] == Round[#2[[1]] / (180 / 200)] &]]
        ][[2, 1]] &,
      polerrTmp]},
   With[
    {paErrQuartiles = Map[
       Select[
         Map[If[Length[#[[2]]] > 2,
            Prepend[Quartiles[#[[2]]], #[[1]]], None] &, #],
         ListQ
        ] &,
       paErr]},
    Block[{t, A, B, C, D, F},
     With[
      {mdls = Table[
         NonlinearModelFit[
          paErrQuartiles[[k, All, {1, dim}]],
          A + B * t + C * t^2 + D * t^3 + F * t^4,
          {A, B, C, D, F},
          t],
         {k, 1, Length@paErrQuartiles},
```

```
              {dim, {2, 3, 4}}]},
            MapThread[
             Function[{models, fillColor, qrtls},
              Plot[
               Evaluate[#[t] & /@ models],
               {t, 0, 180},
               PlotStyle → {{Thin, fillColor}, Black, {Thin, fillColor}},
               Filling → {1 → {{2}, fillColor}, 3 → {{2}, fillColor}},
               Epilog → {Gray, Line[qrtls[[All, {1, 3}]]]},
               PlotRange → {0, 50},
               BaseStyle → Directive[10, FontFamily → "Arial"],
               Axes → None,
               Frame → Table[{True, False}, {2}],
               FrameLabel → {{"Absolute Error [deg]", None},
                 {"Predicted Polar Angle [deg]", None}},
               FrameTicks → {{{0, 10, 20, 30, 40, 50}, None},
                 {{0, 45, 90, 135, 180}, None}},
               AspectRatio → 0.75,
               ImageSize → 2.25 * 72]],
             {mdls, fillColors, paErrQuartiles}]]]]]]},
    Join[ecplots, paplots]]];
Image[
 GraphicsGrid[
  Transpose[
   {{figVoronoiExtErrorLinesEcc1, figVoronoiExtErrorLinesEcc2,
     figVoronoiExtErrorLinesEcc3, figVoronoiExtErrorLinesEccAll},
    {figVoronoiExtErrorLinesPol1, figVoronoiExtErrorLinesPol2,
     figVoronoiExtErrorLinesPol3, figVoronoiExtErrorLinesPolAll}}]],
 ImageResolution → 150]
```

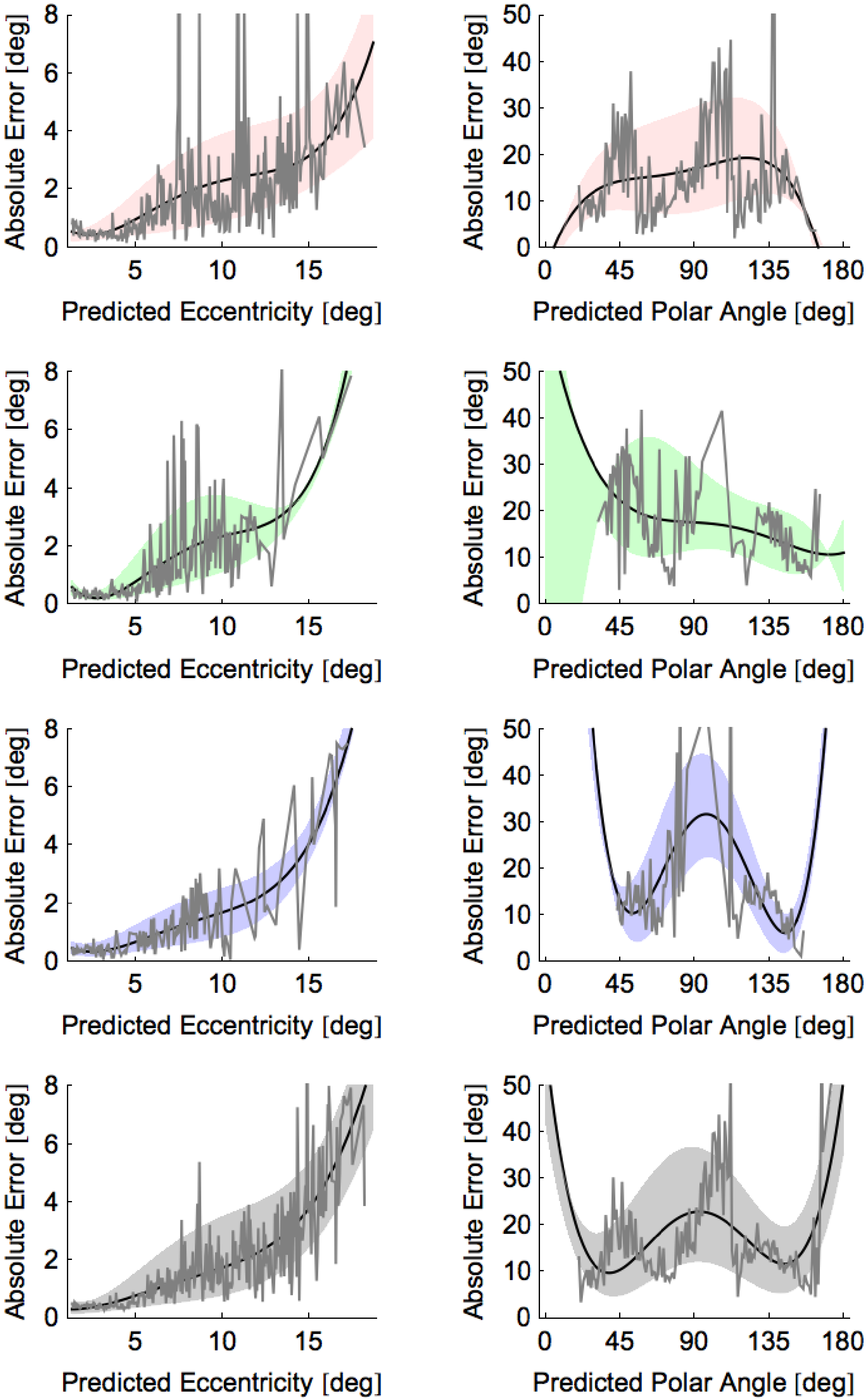
Absolute Error [deg]
8
6
4
2
0
5
10
15
Predicted Eccentricity [deg]
Absolute Error [deg]
50
40
30
20
10
0
0
45
90
135
180
Predicted Polar Angle [deg]
Absolute Error [deg]
8
6
4
2
0
5
10
15
Predicted Eccentricity [deg]
Absolute Error [deg]
50
40
30
20
10
0
0
45
90
135
180
Predicted Polar Angle [deg]
Absolute Error [deg]
8
6
4
2
0
5
10
15
Predicted Eccentricity [deg]
Absolute Error [deg]
50
40
30
20
10
0
0
45
90
135
180
Predicted Polar Angle [deg]
Absolute Error [deg]
8
6
4
2
0
5
10
15
Predicted Eccentricity [deg]
Absolute Error [deg]
50
40
30
20
10
0
0
45
90
135
180
Predicted Polar Angle [deg]

6.6.6. Voronoi Space Histograms

Here, we can see histograms of the leave-one-out error in the predictions of the polar angle and eccentricity.

```
figErr20VoronoiHistograms = GraphicsGrid[
   With[
    {err = AggregateErrors[
       data20,
       sim10[SmoothPrediction],
       {1.25, 8.75},
       12]},
    With[
     {ecerr = err[[{2, 3, 4, 1}, All, All, 4]] /. (Rule[a_, b_] :> {a, b}),
      polerr = err[[{2, 3, 4, 1}, All, All, 3]] /. (Rule[a_, b_] :> {a, b})},
     List[
      MapThread[
       SmoothDensityHistogram[
         #1,
         ImageSize → 3.25 * 72,
         AspectRatio → 1,
         BaseStyle → Directive[10, FontFamily → "Arial"],
         ColorFunction → (If[# > 0.2, Black, Blend[{White, Black}, # / 0.2]] &),
         Axes → None,
         PlotRange → {{1.25, 8.75}, {1.25, 8.75}},
         PlotLabel →
          Style[#2, FontFamily → "Arial", FontSize → 10, FontWeight → Bold],
         Frame → {{True, False}, {True, False}},
         FrameLabel → {
           {"Observed Eccentricity (°)", None},
           {"Predicted Eccentricity (°)", None}}
        ] &,
       {ecerr, {"All", "V1", "V2", "V3"}}],
      MapThread[
       SmoothDensityHistogram[
         #1,
         ImageSize → 3.25 * 72,
         AspectRatio → 1,
         BaseStyle → Directive[10, FontFamily → "Arial"],
         ColorFunction → (If[# > 0.2, Black, Blend[{White, Black}, # / 0.2]] &),
         Axes → None,
         PlotRange → {{0, 180}, {0, 180}},
         PlotLabel →
          Style[#2, FontFamily → "Arial", FontSize → 10, FontWeight → Bold],
         Frame → {{True, False}, {True, False}},
         FrameLabel → {{"Observed Polar Angle (°)", None},
           {"Predicted Polar Angle (°)", None}}
        ] &,
       {polerr, {"All", "V1", "V2", "V3"}}]]]]];
Image[figErr20VoronoiHistograms, ImageResolution → 150]
```

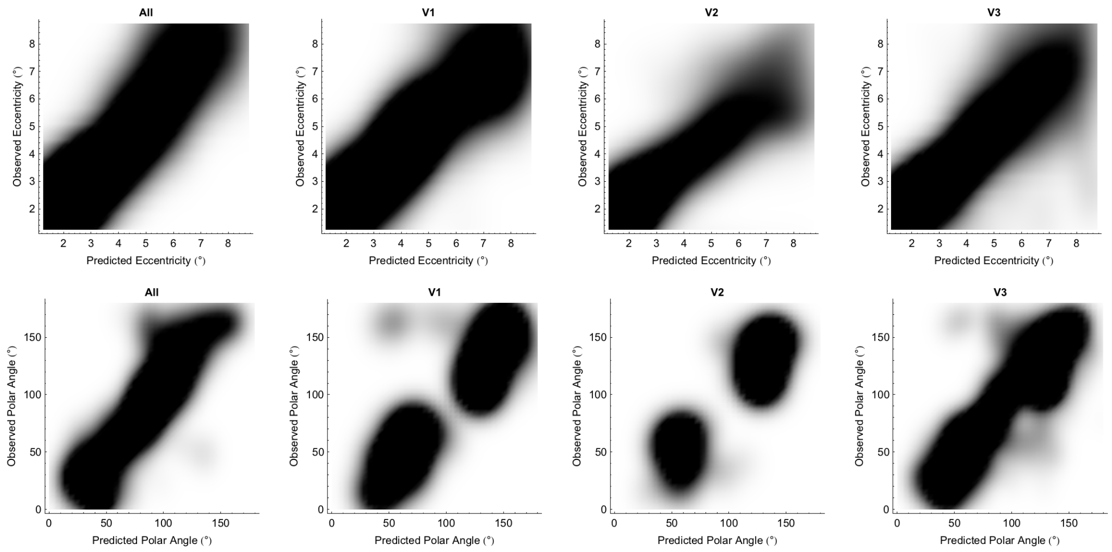

6.7. Test-Retest

Here we plot the test-retest error (ie, the median error between identical 20 minute half-scans across our 19 subjects) on the cortical surface.

```
{figRetestErrsEcc, figRetestErrsPol, figRetestErrsLegend, figRetestErrs} = With[
   {fMin = 0,
    ecMin = 1.25, ecMax = 8.75},
   With[
    {ecTicks = {0, 2, 4},
     paTicks = {0, 25, 50},
     err = Map[
       Join[#[[1, 1 ;; 2]], Transpose[#[[All, 3 ;; 4]]]] &,
       Split[
        Sort[
         Reap[
           Map[
            With[
              {lh1 = #[[1, 1]],
               lh2 = #[[2, 1]],
               rh1 = #[[1, 2]],
               rh2 = #[[2, 2]]},
              MapThread[
               If[#1[[5]] > fMin && #2[[5]] > fMin && 0 < #3[[5]] < 4 &&
                   #1[[3]] ≠ 0 && #2[[3]] ≠ 0 && ecMin ≤ #3[[4]] ≤ ecMax,
                 Sow[
                  Join[#1[[1 ;; 2]], {{#1[[3]], #2[[3]]}, {#1[[4]], #2[[4]]}}],
                  {0, Round[#3[[5]]]}]
                ] &,
               {Join[lh1, rh1],
                Join[lh2, rh2],
                With[{tmp = MatchOrder[sim10[SmoothPredictionFull] → lh1]},
                 Join[tmp, tmp]]}]
             ] &,
            Partition[dataRetest[Data], 2]],
```

```
            {0, 1, 2, 3},
            Apply[Sequence, #2] &
           ][[2, 1]],
          (VertexOrder[#1, #2] ≥ 0) &],
        VertexOrder[#1, #2] == 0 &]]},
   With[
    {paerr = CorticalPlot[
        Map[Append[#[[1 ;; 2]], Median[Abs[#[[3, All, 1]] - #[[3, All, 2]]]]] &, err],
        CortexOutline → $Data10FSAverageOutline,
        ColorFunction → (Which[
            # > Last@paTicks, Darker[Green],
            # > 0, Blend[{White, Darker@Green}, # / Last@paTicks],
            # < -Last@paTicks, Red,
            True, Blend[{White, Red}, Abs[# / Last@paTicks]]] &),
        ImageSize → 1.15 * 72],
      ecerr = CorticalPlot[
        Map[Append[#[[1 ;; 2]], Median[Abs[#[[4, All, 1]] - #[[4, All, 2]]]]] &, err],
        CortexOutline → $Data10FSAverageOutline,
        ColorFunction → (If[# > Last@ecTicks,
            Darker[Green],
            Blend[{White, Darker@Green}, # / Last@ecTicks]] &),
        ImageSize → 1.15 * 72],
      legend = With[
        {emx = Last@ecTicks, pmx = Last@paTicks},
        ArrayPlot[
         Reverse@Table[{k, k}, {k, 0, 1, 0.01}],
         DataRange → {{0, 1}, {0, 1}},
         ImageSize → 0.9 * 72,
         AspectRatio → 5,
         Frame → {{True, True}, {False, False}},
         ColorFunction → (Blend[{White, Darker@Green}, #] &),
         FrameTicks → {{Table[{k / emx, k}, {k, {0, 2, 4}}],
            Table[{k / pmx, k}, {k, {0, 25, 50}}]}, {None, None}},
         FrameLabel → {{None, None}, {"Eccentricity", "Polar Angle"}},
         PlotLabel → Style["Error (°)", FontFamily → "Arial", FontSize → 10],
         BaseStyle → Directive[10, FontFamily → "Arial"]]]},
    {ecerr, paerr, legend,
     Graphics[
      {Inset[legend, ImageScaled[{0.5, 0}], ImageScaled[{0.5, 0}]],
       Inset[ecerr, ImageScaled[{0, 0.55}], ImageScaled[{0, 0.5}]],
       Inset[paerr, ImageScaled[{1, 0.55}], ImageScaled[{1, 0.5}]]},
      ImageSize → {3.4, 1.25} * 72,
      ImagePadding → 2000]}]]];
Image[figRetestErrs, ImageResolution → 150]
```

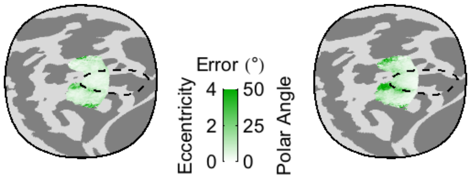

6.8. Schira Model Plots

Here we plot a version of the Schira model.

```
schiraImgWithGray = With[
  {mdl = OurSchiraModel},
  With[
   {fun = SchiraFunction[mdl],
    pr = First[
      FilterRules[
       FullOptions[SchiraLinePlotHash[mdl, PolarAngle]],
       PlotRange]]},
   Show[
    {ParametricPlot[
       {Re@#, Im@#} &[fun[θ, r, 2]],
       {θ, 0, 89.999},
       {r, 0, 90},
       Mesh → False,
       PlotRangeClipping → False,
       pr,
       PlotPoints → 200,
       MeshStyle → None,
       BoundaryStyle → None,
       PlotStyle → Gray,
       Frame → None,
       Axes → None] /. Line[l_List] :→ {Gray, Polygon[l]},
     ParametricPlot[
       {Re@#, Im@#} &[fun[θ, r, 2]],
       {θ, 90.0001, 180},
       {r, 0, 90},
       Mesh → False,
       PlotRangeClipping → False,
       pr,
       PlotPoints → 200,
       MeshStyle → None,
       BoundaryStyle → None,
       PlotStyle → Gray,
       Frame → None,
       Axes → None] /. Line[l_List] :→ {Gray, Polygon[l]},
     ParametricPlot[
       {Re@#, Im@#} &[fun[θ, r, 3]],
       {θ, 0, 89.99999},
       {r, 0, 90},
       Mesh → False,
       PlotRangeClipping → False,
       pr,
       PlotPoints → 200,
       MeshStyle → None,
       BoundaryStyle → None,
       PlotStyle → GrayLevel[0.2],
       Frame → None,
       Axes → None] /. Line[l_List] :→ {GrayLevel[0.2], Polygon[l]},
     ParametricPlot[
       {Re@#, Im@#} &[fun[θ, r, 3]],
       {θ, 90.000001, 180},
       {r, 0, 90},
       Mesh → False,
       PlotRangeClipping → False,
```

```
            pr,
            PlotPoints → 200,
            MeshStyle → None,
            BoundaryStyle → None,
            PlotStyle → GrayLevel[0.2],
            Frame → None,
            Axes → None] /. Line[l_List] :→ {GrayLevel[0.2], Polygon[l]},
         SchiraLinePlot[
          mdl,
          PolarAngleStyleFunction → ({Thick, Darker@$PolarAngleColorFn[#1]} &),
          EccentricityStyleFunction →
           ({Thick, Darker@$EccentricityColorFn[#1]} &)]}]]];
Image[schiraImgWithGray, ImageResolution → 150]
```

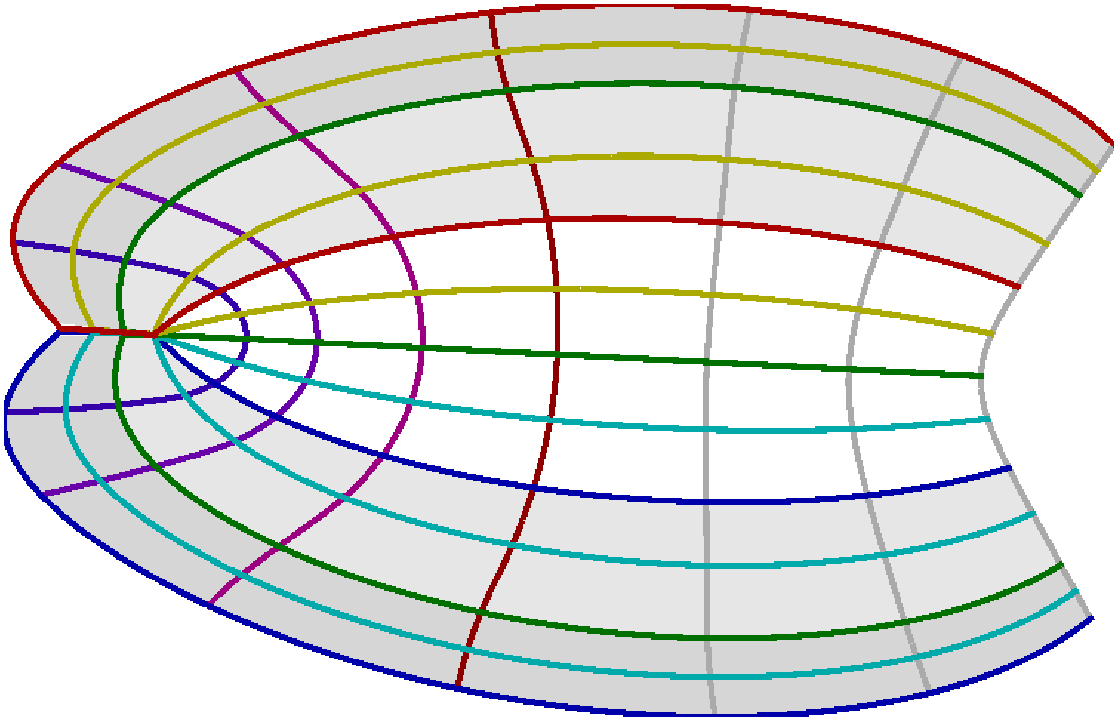

```
Export[
  $FiguresOutputDirectory <> "/schira-model-with-gray-regions.png",
  schiraImgWithGray, "PNG",
  ImageResolution → 600];
```

6.9. Variations in Sulcal Curvature

These show (supplemental) figures of the variation in cortical anatomy across the local cortical surface region. The first shows the mean and standard deviation of the sulcal curvature; the second shows plots of the retinotopic organization of the red-dashed region in part one for each of the 19 subjects.

6.9.1. Deviations of Anatomy in Spring Space

```
{figCurv, curvLegend, figCurvStd} = With[
   {curvMin = -0.4, curvMax = 0.25,
    stdMin = 0, stdMax = 0.65},
   With[
    {dat = Flatten[data10[Data], 1][[All, All, {1, 2, 6}]]},
    With[
     {xy = dat[[1, All, 1 ;; 2]],
      z = Transpose[dat[[All, All, 3]]]},
     {CorticalPlot[
       MapThread[
        Append[#1, Median[#2]] &,
        {xy, z}],
       CortexOutline → $Data10FSAverageOutline,
       ColorFunction →
        (Blend[{Black, White}, 1.0 - (# - curvMin) / (curvMax - curvMin)] &),
       ImageSize → 1.15 * 72,
       Epilog → {
          Red, Dashed,
          Line[{{-0.6, -0.1}, {-0.6, 0.5}, {0, 0.5}, {0, -0.1}, {-0.6, -0.1}}]}],
      ArrayPlot[
       Table[{k, k}, {k, -0.4, 0.25, 0.0125}],
       DataRange → {{0, 1}, {-0.4, 0.25}},
       ImageSize → 1.3 * 72,
       AspectRatio → 6,
       ColorFunctionScaling → False,
       ColorFunction →
        (Blend[{Black, White}, 1.0 - (# - curvMin) / (curvMax - curvMin)] &),
       Frame → {{True, True}, {False, False}},
       FrameLabel → {{None, None}, {"Std. Dev. (mm^-1)", "Curvature (mm^-1)"}},
       BaseStyle → Directive[10, FontFamily → "Arial"],
       FrameTicks → {
          {{{-0.4, "0.0"}, {-0.2, "0.2"}, {0, "0.4"}, {0.2, "0.6"}},
           {{-0.4, "-0.4"}, {-0.2, "-0.2"}, {0, "  0.0"}, {0.2, "  0.2"}}},
          {False, False}}],
      CorticalPlot[
       MapThread[
        Append[#1, StandardDeviation[#2]] &,
        {xy, z}],
       CortexOutline → $Data10FSAverageOutline,
       ColorFunction → (Blend[{Black, White}, (# - stdMin) / (stdMax - stdMin)] &),
       ImageSize → 1.15 * 72]}]]];
Image[
 Graphics[
  {Inset[figCurvStd, ImageScaled[{0, 0.5}], ImageScaled[{0, 0.5}]],
   Inset[curvLegend, ImageScaled[{0.5, 0.5}], ImageScaled[{0.5, 0.5}]],
   Inset[figCurv, ImageScaled[{1, 0.5}], ImageScaled[{1, 0.5}]]},
  ImageSize → {3.8, 1.25} * 72,
  ImagePadding → 10 000],
 ImageResolution → 150]
```

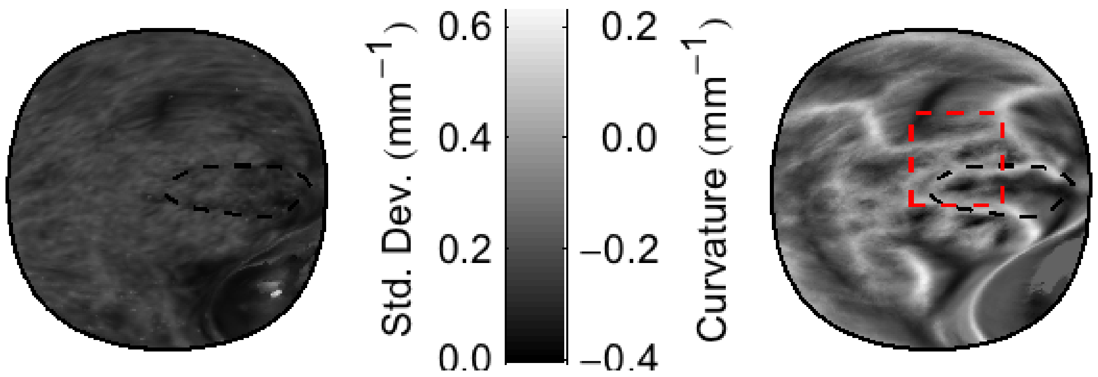

6.9.2. Differences at the V3/V3A border

```
figV3V3ADiff = With[
    {imgs = Apply[
        Append[Flatten@#2, #1] &,
        Reap[
         With[
          {idx = Flatten[
              Position[
               LocalAnatomy,
               {x_, y_, _} /; And[-0.6 ≤ x ≤ 0.0, -0.1 ≤ y ≤ 0.5]]]},
          Show[
           Map[
            With[
              {d = MeanJointMap[#[[All, idx]]]},
              Sow[
               ListContourPlot[
                d[[All, 1 ;; 3]],
                ImageSize → 72,
                AspectRatio → 1,
                ColorFunctionScaling → False,
                ColorFunction → (Hue[2. / 3. * (1 - # / 180.0)] &),
                Frame → False,
                Contours → {30, 60, 90, 120, 150}]];
              ListContourPlot[
               d[[All, 1 ;; 3]],
               ImageSize → 72,
               AspectRatio → 1,
               ContourShading → False,
               Contours → {0, 150, 180},
               Frame → False,
               Axes → False]
             ] &,
            data10[Data]]]]]]},
    GraphicsGrid[
     Partition[imgs, 5],
     ImageSize → 6.5 * 72]];
Image[figV3V3ADiff, ImageResolution → 150]
```

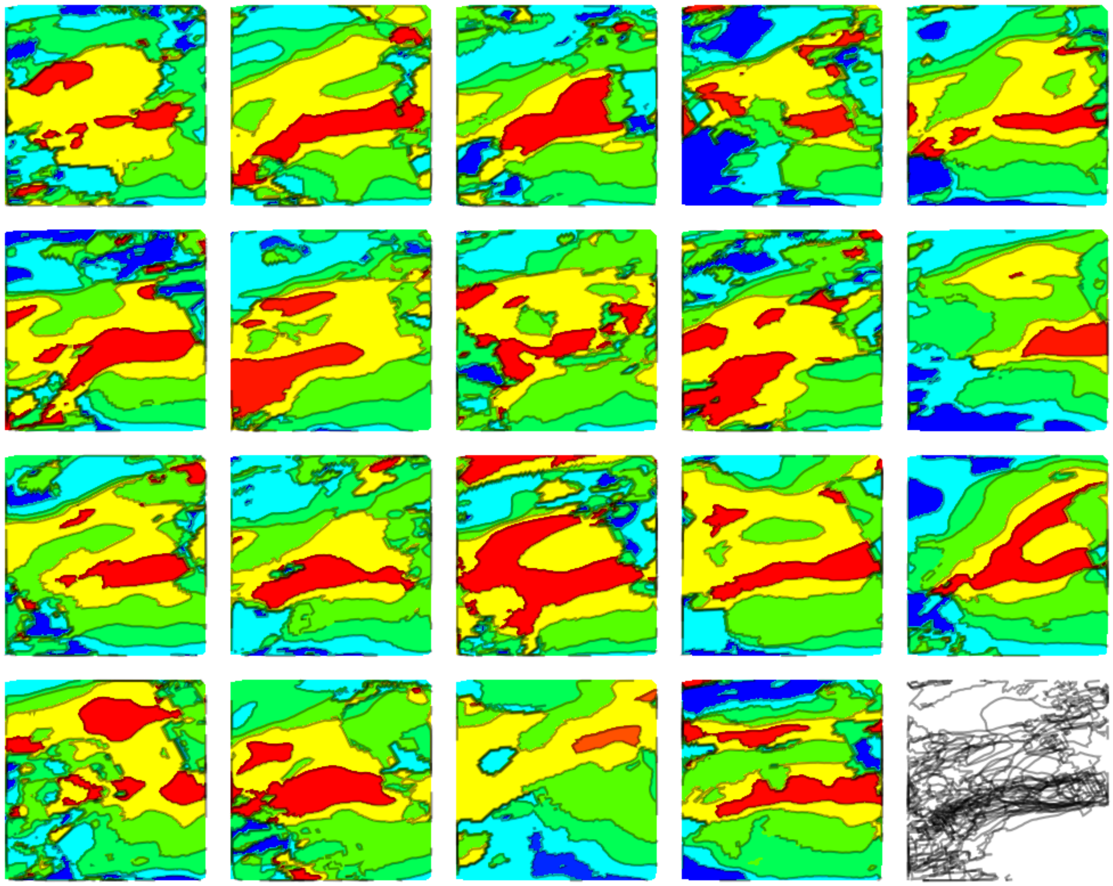

6.10. Export Figures

This section simply holds the code to export the figures generated above. They are exported in parts, and are stored here mostly so that, in case you don’t want to export the figures, it’s easier to run them in batch without picking through the code above.

```
Options[ExportFigure] = {
   Format → Automatic,
   ImageResolution → 600};
ExportFigure[Rule[fig_, filename_], OptionsPattern[]] := With[
   {fmt = Replace[
      OptionValue[Format],
      {Automatic :→ Switch[
         ToUpperCase[Last[StringSplit[filename, "."]]],
         "PNG", "PNG",
         "PDF", "PDF",
         "TIFF", "TIFF", "TIF", "TIFF",
         "POSTSCRIPT", "PS", "PS", "PS",
         "GIF", "GIF",
         "JPG", "JPEG",
         "JPEG", "JPEG",
         _, Die[
          "Unrecognized format (Note that ExportFigure does
            not recognize all Mathematica formats)"]],
       x_ :→ x}]},
   Export[
    $FiguresOutputDirectory <> "/" <> filename,
    fig,
    fmt,
    ImageResolution → OptionValue[ImageResolution]]];

Scan[
  If[ValueQ[#[[1]]], ExportFigure[#]] &,
  Unevaluated[
   {figRegionsFSAverage → "space=FS_dataset=10_plot=regions.png",
    figRegionsCorrected → "space=SS_dataset=10_plot=regions.png",
    figData10AggEcc → "space=FS_data=10_plot=eccen.png",
    figData10AggPol → "space=FS_data=10_plot=angle.png",
    figData10SSEcc → "space=SS_data=10_plot=eccen.png",
    figData10SSPol → "space=SS_data=10_plot=angle.png",
    figData10VoronoiEcc → "space=voronoi_data=10_plot=eccen.png",
    figData10VoronoiPol → "space=voronoi_data=10_plot=angle.png",
    figSSNormDist → "spring-distortion_data=10_plot=norm.png",
    figSSDirDist → "spring-distortion_data=10_plot=direction.png",
    figVoronoiNormDist → "voronoi-distortion_data=10_plot=norm.png",
    figVoronoiDirDist → "voronoi-distortion_data=10_plot=direction.png",
    figData20SSEcc → "space=SS_data=20_plot=eccen.png",
    figData20SSPol → "space=SS_data=20_plot=angle.png",
    figLOOErrsEcc → "space=FS_data=10_plot=eccenError.png",
    figLOOErrsPol → "space=FS_data=10_plot=angleError.png",
    figLOOErrsLegend → "space=FS_data=10_plot=errorLegend.png",
    figLOOErrorLinesEcc1 → "errors_data=10_plot=eccen_region=V1.pdf",
    figLOOErrorLinesEcc2 → "errors_data=10_plot=eccen_region=V2.pdf",
    figLOOErrorLinesEcc3 → "errors_data=10_plot=eccen_region=V3.pdf",
    figLOOErrorLinesEccAll → "errors_data=10_plot=eccen_region=All.pdf",
    figLOOErrorLinesPol1 → "errors_data=10_plot=angle_region=V1.pdf",
    figLOOErrorLinesPol2 → "errors_data=10_plot=angle_region=V2.pdf",
    figLOOErrorLinesPol3 → "errors_data=10_plot=angle_region=V3.pdf",
    figLOOErrorLinesPolAll → "errors_data=10_plot=angle_region=All.pdf",
```

```
figVoronoiLOOErrorLinesEcc1 →
 "voronoi-errors_data=10_plot=eccen_region=V1.pdf",
figVoronoiLOOErrorLinesEcc2 →
 "voronoi-errors_data=10_plot=eccen_region=V2.pdf",
figVoronoiLOOErrorLinesEcc3 →
 "voronoi-errors_data=10_plot=eccen_region=V3.pdf",
figVoronoiLOOErrorLinesEccAll →
 "voronoi-errors_data=10_plot=eccen_region=All.pdf",
figVoronoiLOOErrorLinesPol1 →
 "voronoi-errors_data=10_plot=angle_region=V1.pdf",
figVoronoiLOOErrorLinesPol2 →
 "voronoi-errors_data=10_plot=angle_region=V2.pdf",
figVoronoiLOOErrorLinesPol3 →
 "voronoi-errors_data=10_plot=angle_region=V3.pdf",
figVoronoiLOOErrorLinesPolAll →
 "voronoi-errors_data=10_plot=angle_region=All.pdf",
figRetestErrsEcc → "space=FS_data=retest_plot=eccenError.png",
figRetestErrsPol → "space=FS_data=retest_plot=angleError.png",
figRetestErrsLegend → "space=FS_data=retest_plot=errorLegend.png",
figCurv → "space=FS_data=10_plot=curv.png",
figCurvStd → "space=FS_data=10_plot=curvStd.png",
figV3V3ADiff → "V3-V3A-Border.png",
curvLegend → "curv-legend.png",
polLegend → "angle-legend.png",
eccLegend → "eccen-legend.png"}]];
```

7. Exporting VTK Files

7.1. Function for exporting VTK files

We can export a VTK file by reading in another VTK file, mapping each of its points to a value, and writing out the resulting data. This allows us to create a spherical brain VTK file despite the fact that our data has been transformed. In order to facilitate this, we require that the function that maps points to a value accept, as input arguments, 3 lists: the original (x,y,z) coordinates of the points in the VTK file, the converted (θ,ϕ) coordinates of the flattened points, and the values of the points in the vtk file. It must return a list of values to be written out.

```
VTKExport[flnm_String, convert_Function, outnm_String] := Module[
  {v = ReadVTK[flnm],
   vals,
   e = Import[flnm, "PolygonData"],
   f = OpenWrite[outnm]},
  vals = convert[
    v[[All, 1]],
    V1Transform[v[[All, 1]]],
    v[[All, 2, 1]]];
  WriteString[f, "# vtk DataFile Version 1.0\n"];
  WriteString[f, "vtk output\n"];
  WriteString[f, "ASCII\n"];
  WriteString[f, "DATASET POLYDATA\n"];
  WriteString[f, "POINTS ", Length@v, " float\n"];
  Scan[
   WriteString[f,
     NumberForm[#[[1]], {8, 5}, NumberPadding → {"", "0"}], "  ",
     NumberForm[#[[2]], {8, 5}, NumberPadding → {"", "0"}], "  ",
     NumberForm[#[[3]], {8, 5}, NumberPadding → {"", "0"}], "\n"] &,
   v[[All, 1]]];
  WriteString[f, "POLYGONS ",
   Length@e, " ", Length@e + Length[Flatten[e]], "\n"];
  Scan[
   WriteString[f, Length@#,
     Apply[StringJoin, Flatten[Map[{" ", ToString[#]} &, # - 1]]], "\n"] &,
   e];
  WriteString[f, "POINT_DATA ", Length@v, "\n"];
  WriteString[f, "FIELD FieldData 1\n"];
  WriteString[f, "curv 1 ", Length@v, " double\n"];
  Scan[
   WriteString[f, NumberForm[#, {8, 5},
      NumberPadding → {"", "0"}, ExponentFunction → (Null &)], "\n"] &,
   vals];
  Close[f]];
```

7.2. Export the Template Files

7.2.1. Export the areas file

```
With[{smooth = sim10[SmoothPrediction], tol = 0.0000001},
  VTKExport[
   $MetadataDirectory <> "/lh.mgh_index.vtk",
   Function[{pts3d, pts2d, vals},
    Block[{A, B},
     Module[
      {p = Select[smooth[[All, {1, 2, 5}]], NumberQ[#[[3]]] &],
       tmp = Table[0, {Length@vals}],
       pts = MapIndexed[Join, pts2d]},
      Scan[
       Function[{d},
        Which[
         Length@d == 2 && Head[d[[1]]] === A && Head[d[[2]]] === B,
         tmp[[d[[1, 3]]]] = d[[2, 3]],
         Length@d == 2 && Head[d[[1]]] === B && Head[d[[2]]] === A,
         tmp[[d[[2, 3]]]] == d[[1, 3]]]],
       Split[
        Sort[
         Join[
          Map[Apply[A, #] &, pts],
          Map[Apply[B, #] &, p]],
         Which[
           #1[[1]] - #2[[1]] < -tol, True,
           #1[[1]] - #2[[1]] > tol, False,
           #1[[2]] - #2[[2]] < -tol, True,
           #1[[2]] - #2[[2]] > tol, False,
           Head[#1] === A, True,
           True, False] &],
        Apply[List, #1[[1 ;; 2]]] == Apply[List, #2[[1 ;; 2]]] &]];
      tmp]]],
   $OutputDirectory <> "/areas-template.vtk"]];
```

7.2.2. Export the angle file

```
With[{smooth = sim10[SmoothPrediction], tol = 0.0000001},
  VTKExport[
   $MetadataDirectory <> "/lh.mgh_index.vtk",
   Function[{pts3d, pts2d, vals},
    Block[{A, B},
     Module[
      {p = Select[smooth[[All, {1, 2, 3}]], NumberQ[#[[3]]] &],
       tmp = Table[0, {Length@vals}],
       pts = MapIndexed[Join, pts2d]},
      Scan[
       Function[{d},
        Which[
         Length@d == 2 && Head[d[[1]]] === A && Head[d[[2]]] === B,
         tmp[[d[[1, 3]]]] = d[[2, 3]],
         Length@d == 2 && Head[d[[1]]] === B && Head[d[[2]]] === A,
         tmp[[d[[2, 3]]]] == d[[1, 3]]]],
       Split[
        Sort[
         Join[
          Map[Apply[A, #] &, pts],
          Map[Apply[B, #] &, p]],
         Which[
           #1[[1]] - #2[[1]] < -tol, True,
           #1[[1]] - #2[[1]] > tol, False,
           #1[[2]] - #2[[2]] < -tol, True,
           #1[[2]] - #2[[2]] > tol, False,
           Head[#1] === A, True,
           True, False] &],
        Apply[List, #1[[1 ;; 2]]] == Apply[List, #2[[1 ;; 2]]] &]];
      tmp]]],
   $OutputDirectory <> "/angle-template.vtk"]];
```

7.2.3. Export the eccen file

```
With[{smooth = sim10[SmoothPrediction], tol = 0.0000001},
  VTKExport[
   $MetadataDirectory <> "/lh.mgh_index.vtk",
   Function[{pts3d, pts2d, vals},
    Block[{A, B},
     Module[
      {p = Select[smooth[[All, {1, 2, 4}]], (NumberQ[#[[3]]]) &],
       tmp = Table[0, {Length@vals}],
       pts = MapIndexed[Join, pts2d]},
      Scan[
       Function[{d},
        Which[
         Length@d == 2 && Head[d[[1]]] === A && Head[d[[2]]] === B,
         tmp[[d[[1, 3]]]] = d[[2, 3]],
         Length@d == 2 && Head[d[[1]]] === B && Head[d[[2]]] === A,
         tmp[[d[[2, 3]]]] == d[[1, 3]]]],
       Split[
        Sort[
         Join[
          Map[Apply[A, #] &, pts],
          Map[Apply[B, #] &, p]],
         Which[
           #1[[1]] - #2[[1]] < -tol, True,
           #1[[1]] - #2[[1]] > tol, False,
           #1[[2]] - #2[[2]] < -tol, True,
           #1[[2]] - #2[[2]] > tol, False,
           Head[#1] === A, True,
           True, False] &],
        Apply[List, #1[[1 ;; 2]]] == Apply[List, #2[[1 ;; 2]]] &]];
      tmp]]],
   $OutputDirectory <> "/eccen-template.vtk"]];
```

7.2.4. Export the distortion file

```
With[
  {dat = MapThread[
     {#1[[1]], #1[[2]], Norm[#1[[1 ;; 2]] - #2[[1 ;; 2]]]} &,
     {LocalAnatomy, sim10[Result]}],
   tol = 0.0000001},
  VTKExport[
   $MetadataDirectory <> "/lh.mgh_index.vtk",
   Function[{pts3d, pts2d, vals},
    Block[{A, B},
     Module[
      {p = Select[dat, (NumberQ[#[[3]]]) &],
       tmp = Table[0, {Length@vals}],
       pts = MapIndexed[Join, pts2d]},
      Scan[
       Function[{d},
        Which[
         Length@d == 2 && Head[d[[1]]] === A && Head[d[[2]]] === B,
         tmp[[d[[1, 3]]]] = d[[2, 3]],
         Length@d == 2 && Head[d[[1]]] === B && Head[d[[2]]] === A,
         tmp[[d[[2, 3]]]] == d[[1, 3]]]],
       Split[
        Sort[
         Join[
          Map[Apply[A, #] &, pts],
          Map[Apply[B, #] &, p]],
         Which[
           #1[[1]] - #2[[1]] < -tol, True,
           #1[[1]] - #2[[1]] > tol, False,
           #1[[2]] - #2[[2]] < -tol, True,
           #1[[2]] - #2[[2]] > tol, False,
           Head[#1] === A, True,
           True, False] &],
        Norm[Apply[List, #1[[1 ;; 2]]] - Apply[List, #2[[1 ;; 2]]]] < tol &]];
      tmp]]],
   $OutputDirectory <> "/distortion-template.vtk"]];
```
