# Supplementary material for: Correction of Distortion in Flattened Representations of the Cortical Surface Allows Prediction of V1-V3 Functional Organization from Anatomy
Source: PLoS Comput Biol. 2014 Mar 27;10(3):e1003538. doi: 10.1371/journal.pcbi.1003538 (PMC3967932; doi:10.1371/journal.pcbi.1003538)
Supplement: Text S1 — Supplemental notes regarding the parameterization of the simulation and Schira model. This document provides a brief description of our simulation and model parameterization as well as information about where to find additional materials such as source code. (DOC) [file pcbi.1003538.s006.doc]

**Correction of distortion in flattened representations of the cortical surface allows prediction of V1-V3 functional organization from anatomy:**

**Supplemental Text S1**

Noah C. Benson^1,2^, Omar H. Butt^1^, David H. Brainard^2^, Geoffrey K. Aguirre1,[[1]](#footnote-2)*

Departments of ^1^Neurology and ^2^Psychology

University of Pennsylvania

Philadelphia, PA 19104

**Parameterization of the Schira Model**

For use in this paper, we extended the standard parameters of the Schira model to include additional terms: rotation, horizontal stretch, vertical stretch, and translation. Because a horizontal shear of 0.65 was applied to all vertices during preprocessing, we did not find that we needed additional shear parameters in the Schira model itself. The full parameterization of the Schira model used in this paper, along with a small library of functions for plotting, analyzing, and inverting the Schira model, is provided in the Supplemental *Mathematica* Notebook (§1.6); for all parameters, the default values defined in the notebook are the ones used here.

Prior to the mass-spring-damper (MSD) simulations used to register the functional data to the Schira model, we attempted to find a fit of the Schira model parameters to our functional polar angle and eccentricity data. This goal of this fit was not to optimize the error in the Schira model, but rather to optimize the registration procedure that followed. The primary concerns were thus that the initial potential gradients of each of the vertices in the simulation were such that as many vertices as possible would be immediately pulled toward their correct location in the Schira model (*i.e.*, toward the predicted position of the vertices in correct retinotopic area: V1, V2, or V3). While we found that some parameterizations of the Schira model produced superior fits to the one we ultimately used, most of these also caused unintended effects early in the simulation due to some points falling on the wrong side of a V1/V2 or V2/V3 boundary. Such initial “bad starts” can require extremely long simulations to correct. The parameterization used here was reached by examining the potential gradients of the simulation at time 0. Parameterizations that encouraged distant vertices with similar polar angle and eccentricity measurements to converge at a single location were discarded.

**Simulation Parameterization**

The MSD simulations used to register the functional retinotopy data to the Schira model contain several parameters whose values were chosen manually. These parameters are detailed in the main text of the paper and can also be examined in the simulation code provided on gitHub (<https://github.com/noahbenson/SpringRegister/>); they include the length of the timestep used in numerical integration, the strength of both anatomical and model springs, the duration of the simulations, the amount of kinetic energy added to the simulation prior to each simulated annealing step, and several others. While the values chosen for each of these parameters are “magic numbers” in that we do not have a theoretical framework from which they were chosen, they do nonetheless result in a stable simulation. Additionally, we performed tests in which we ran several additional simulations with small deviations in each of the parameters individually. While these simulations did not always give identical answers, they were all stable. Unstable parameterizations are not difficult to find, but neither are they difficult to identify, as the potential energy in such parameterizations tends to grow quickly. This occurs because a steep potential gradient combined with a large timestep can push a configuration farther past its local potential minimum than it started during each simulation step.

All code used to run the simulations in this paper, as well as the parameters used to run them, are available on our website. This code is written in C++ and is fast, lightweight, and highly parallelizable. All simulations used in this paper required less than a day of computing time on a 2009 27” iMac. Although this code is designed for use with the Schira model, it is written in a general way that allows one to easily construct novel force fields for use in simulation. It is our hope that it can be of use to other researchers interested in applying 2D models of functional organization to the cortical surface.

1. ^*^ To whom correspondences should be addressed. Email: [**aguirreg@mail.med.upenn.edu**](mailto:aguirreg@mail.med.upenn.edu) [↑](#footnote-ref-2)
